# Taxonomy assignment approach determines the efficiency of identification of OTUs in marine nematodes

**DOI:** 10.1098/rsos.170315

**Published:** 2017-08-16

**Authors:** Oleksandr Holovachov, Quiterie Haenel, Sarah J. Bourlat, Ulf Jondelius

**Affiliations:** 1 Department of Zoology, Swedish Museum of Natural History, Stockholm, Sweden; 2 Zoological Institute, University of Basel, Basel, Switzerland; 3 Department of Marine Sciences, University of Gothenburg, Gothenburg, Sweden

**Keywords:** biodiversity, identification, barcode, nematodes, metabarcoding, meiobenthos

## Abstract

Precision and reliability of barcode-based biodiversity assessment can be affected at several steps during acquisition and analysis of data. Identification of operational taxonomic units (OTUs) is one of the crucial steps in the process and can be accomplished using several different approaches, namely, alignment-based, probabilistic, tree-based and phylogeny-based. The number of identified sequences in the reference databases affects the precision of identification. This paper compares the identification of marine nematode OTUs using alignment-based, tree-based and phylogeny-based approaches. Because the nematode reference dataset is limited in its taxonomic scope, OTUs can only be assigned to higher taxonomic categories, families. The phylogeny-based approach using the evolutionary placement algorithm provided the largest number of positively assigned OTUs and was least affected by erroneous sequences and limitations of reference data, compared to alignment-based and tree-based approaches.

## 1. Introduction

Metabarcoding studies based on high-throughput sequencing of amplicons from marine samples have reshaped our understanding of the biodiversity of marine microscopic eukaryotes, revealing a much higher diversity than previously known [1]. Early metabarcoding of the slightly larger sediment-dwelling meiofauna has mainly focused on scoring the relative diversity of taxonomic groups [1–3]. The next step in metabarcoding, identification of species, is limited by the available reference database, which is sparse for most marine taxa, and by the matching algorithms. In this paper, we are evaluating to what extent sequences of unidentified putative species (operational taxonomic units, OTUs) of marine nematodes can be assigned to family-level taxa using publicly available reference sequences, and which of three matching strategies, alignment-based, tree-based or phylogeny-based, provides the highest number of identified OTUs.

The reference datasets for marine nematodes are sparsely populated, as correctly pointed out in Dell'Anno *et al*. [4]. The most recent check of NCBI GenBank (February 2017) reveals that less than 180 genera and about 170 identified species of marine nematodes are included, compared to over 530 described genera and almost 4750 described species (based on [5] with updates). This summarized number of records in GenBank does not take into consideration which genes are represented (mostly near complete or partial 18S and partial 28S rDNA), but gives the total number of entries. Not all of these entries include sequences suitable to be used as references for metabarcoding. As completeness of the reference databases for marine nematodes is insufficient to assign all OTUs to species level [6], one has to consider if they can be assigned to taxonomic categories above species level, and if this type of data can be used in research.

Assignment of OTUs to nematode genera faces the same problem as the assignment of OTUs to species—limited representation of identified taxa in reference databases (see above). Identification to the family level of those OTUs that cannot be assigned to any particular species or genus is the next best option. It provides enough information to group nematode OTUs into trophic [7,8] and functional [9] groups and apply ecological metrics, such as Maturity Index [10], used to evaluate the complexity and functioning of nematode communities [11]. This approach has already been applied in metabarcoding studies of terrestrial nematode communities from the Arctic and the tropics [12,13].

Although it would be possible to generate new barcodes for marine nematodes from our study sites to supplement existing reference datasets, the purpose of this paper is to follow the typical scenario when metabarcoding projects rely on existing databases and do not publish new reference sequences.

Identification of OTUs can be done using a number of currently available approaches and applications, several of which will be tested and compared below. In general, all taxonomy assignment methods can be grouped into four categories: alignment-based, probabilistic, tree-based and phylogeny-based.

*Alignment-based* approaches use various measures of similarity between query and reference sequences based solely on their alignment. They are implemented in VAMPS [14], Taxonerator [15] and CREST [16], or can be performed directly through BLASTN [17] function of the NCBI server (https://blast.ncbi.nlm.nih.gov/Blast.cgi). The performances of CREST and BLASTN are evaluated in detail in this publication. On the other hand, because VAMPS is specifically designed for prokaryotic organisms, while Taxonerator uses the same routine as BLASTN, neither one is included in this comparison.

*Probabilistic* approaches rely on likelihood estimates of OTU placement and include the UTAX algorithm of the USEARCH software package [18] and Statistical Assignment Package (SAP) [19]. For technical reasons, none of these tools are included in this comparison: (i) exact details of the UTAX algorithm have not been published, and thus the results produced by this approach are difficult to evaluate; (ii) a standalone version of SAP could not be successfully installed, while the web server (http://services.birc.au.dk/sap/server) was not stable in use and consistently returned error messages.

The *tree-based* approach evaluates the similarity between query and reference sequences by analysing the position of each individual OTU relative to the reference sequence on the phylogram and the bootstrap support that it receives. This approach includes the following bioinformatic steps: multiple sequence alignment of short query reads with reference sequences is done de novo using any available multiple sequence alignment tool; the dataset is usually trimmed to the barcode size; the phylogram is built using one of the phylogeny inference algorithms, most commonly Neighbour Joining, followed by bootstrapping [20–25].

*Phylogeny-based* identification of query sequences is performed in three stages. During the preparation stage, a manually curated reference alignment is created using full-length sequences of the gene that includes the barcoding region. A reference phylogeny is estimated based on this alignment. Taxonomic assignment of the query barcodes is then done by using the reference tree as a constraint and testing placement of query reads across all nodes in the reference topology, with the placement likelihood calculated for every combination. The highest scoring placements are retained for evaluation. This approach is implemented in MLTreeMap [26], pplacer [27] and Evolutionary Placement Algorithm (EPA) [28]. Of the three, only the EPA is used in this paper, because ‘there was no clear difference in accuracy between EPA and pplacer’ (cited from [27]) in comparative tests performed [28]. MLTreeMap is designed for taxonomy assignment of barcodes into higher-level taxonomic categories (phylum and above) and was not suitable for our purpose.

## 2. Material and methods

### 2.1. Sampling sites, sampling, extraction and fixation

Samples used in this study were collected in two ecologically distinct locations along the west coast of Sweden. Coarse shell sand was sampled at 7–8 m depth with a bottom dredge along the northeastern side of the Hållö island near Smögen (N 58° 20.32–20.38′ E 11° 12.73–12.68′). Soft mud was collected using a Warén dredge at 53 m depth in the Gullmarn Fjord near Lysekil (N 58° 15.73′ E 11° 26.10′), in the so-called ‘Telekabeln’ site. Samples from both sites were extracted using two different techniques each. Material for metabarcoding was preserved in 96% ethanol and stored at −20°C; material for morphology-based identification was preserved in 4% formaldehyde.

The meiofauna from the coarse sand from Hållö was extracted using two variations of the flotation (decanting and sieving) technique. In the first case, fresh water was used to induce osmotic shock in meiofaunal organisms and force them to detach from the substrate. A volume of 200 ml of sediment was placed in a large volume of fresh water, and thoroughly mixed to suspend meiofauna and sediment. The supernatant was sieved through a 1000 µm sieve in order to separate and discard the macrofaunal fraction. The filtered sample was then sieved through a 45 µm sieve to collect the meiofauna, which was preserved either for sequencing or morphological identification. The sieving step was repeated three times. Ten replicates were preserved for molecular studies and two replicates were preserved for morphology-based observations. In the second case, a 7.2% solution of MgCl_2_ was used to anaesthetize nematodes and other organisms to detach them from the substrate. The meiofauna was decanted through a 125 µm sieve. Similarly, 10 replicates were preserved for molecular studies and two replicates were preserved for morphology-based observations.

The meiofauna from the mud samples was also extracted using two different methods: floatation and siphoning. For the floatation, fresh water was used to induce osmotic shock in meiofaunal organisms. A volume of 2.4 l of sediment was placed in a large volume of fresh water, and thoroughly mixed to suspend the meiofauna and sediment. The supernatant was sieved through a 1000 µm sieve in order to separate and discard the macrofaunal fraction. The filtered sample was then sieved through a 70 µm sieve to collect the meiofauna. The last procedure was repeated three times. The meiofauna was collected, divided into 12 subsamples and preserved: six subsamples were preserved for molecular studies and six subsamples were preserved for morphology-based observations. For siphoning, a total volume of 12 l of sediment was transferred to a plastic container, covered with 20 cm of seawater and left to settle overnight. The meiofauna was then collected through siphoning off the top layer of sediment and passing it through a 125 µm sieve from which samples were taken. One sample was fixed in 96% ethanol, and split into six equal subsamples for molecular studies. The second sample was also split into six subsamples and preserved for morphology-based observations.

### 2.2. Morphology-based analysis of samples

To estimate nematode diversity, it is usually recommended to count and identify all nematode individuals either in the entire sample or in a subsample of a predetermined volume. The alternative, least time-consuming and most commonly used option is to count a predetermined number (usually 100 or 200) of randomly picked nematodes from the sample. Unfortunately, this latter approach can be imprecise for samples with high species diversity. Moreover, because nematodes are affected by Stokes law, which causes uneven distribution of specimens of different size along the bottom of the counting dish, it is difficult to obtain randomized data with this approach. Therefore, we opted to count and identify all nematodes for all samples (or subsamples). The amount of time required for this task limited the effort to two replicates for each site and extraction method, eight in total. We appreciate that counting nematodes in only two replicates per sample is not enough to quantitatively evaluate the composition of nematode communities; it is nevertheless satisfactory to provide the list of species and genera for each sampling site and extraction method for the purpose of this publication.

All nematode specimens were identified and counted for two replicates each from Hållö floatation with MgCl_2_, Hållö floatation with fresh water and Telekabeln siphoning. Telekabeln floatation with freshwater was subsampled by taking 1/10 of the entire sample. Specimens from formaldehyde-preserved samples were transferred to pure glycerine using a modified Seinhorst rapid method [29] and mounted on glass slides using the paraffin wax ring method. All nematode specimens were identified to genus and, when possible, to species level and placed in the classification system published in Schmidt-Rhaesa [5] and accepted in WoRMS [30] and NeMys [31] reference databases. Note that this classification is in many cases different from the nematode classification used in GenBank [32], SILVA [33] and GBIF (www.gbif.org).

### 2.3. Sequencing procedures

Several different markers are used in barcoding and metabarcoding of biota, including mitochondrial cytochrome c oxidase subunit 1 (COI) [34], ITS rRNA [35], multiple regions of 18S rRNA [1] and 28S rRNA [24,36]. Nematode sequences used in this publication were generated as part of a larger NGS-based meiofauna survey [6], which included sequencing and comparative analysis of both standard animal barcode COI [34] and a marker encompassing a V1--V2 variable region of the 18S rRNA gene originally proposed for barcoding of nematodes [37]. The 18S rRNA sequence was chosen for subsequent analysis for the following reasons: (i) the 18S rRNA (V1--V2) region had a higher sequencing success rate in nematodes with 139 OTUs versus only 22 COI OTUs generated using two different sets of primers [6]; (ii) the reference dataset for marine nematodes includes over 300 high-quality 18S sequences obtainable from GenBank, whereas only about 60 COI barcodes of marine nematodes are available in BOLD; (iii) this particular genetic marker is commonly used in metabarcoding studies of marine meiofauna [2,3,6,38] and plankton [39].

DNA extractions from the samples preserved in 96% ethanol were performed on about 10 g of sediment using the PowerMax® Soil DNA Isolation Kit, (MO BIO Laboratories), according to the manufacturer's instructions. Primers were designed for the 18S rRNA gene including Illumina MiSeq overhang adapter sequences for compatibility with Illumina index and sequencing adapters. The 18S rRNA marker was amplified using PCR primers modified from Fonseca *et al.* [2] yielding an approximately 370 bp fragment that includes the V1--V2 hypervariable domains of 18S rRNA (electronic supplementary material, figure S1). Illumina MiSeq library preparation was done using the dual PCR amplification method [40]. All subsequent sequencing and bioinformatic analysis steps are fully described in Haenel *et al.* [6].

### 2.4. Preliminary taxonomic assignment using QIIME

Preliminary taxonomic assignment was done using the QIIME [41] script *assign_taxonomy.py* against the SILVA database [33] release 111 in order to identify and separate nematode OTUs from the total of 1472 18S OTUs of meiofauna generated during a previous step [6]. Default settings in QIIME used for preliminary sorting of OTUs grouped query sequences into two groups based on similarity level: to phyla at 80% similarity and to species at 97% similarity. The output for each query sequence included the closest match but did not give the similarity level, making it impossible to evaluate these assignments. Only two OTUs were positively identified using QIIME to species level: *Viscosia viscosa* (TS6.SSU58722) and *Chromadora nudicapitata* (HF2.SSU192072). Six more OTUs were identified to the genus level: *Enoplus* sp. (HE3.SSU110275), *Enoploides* sp. (HE3.SSU124287), *Symplocostoma* sp. (HE5.SSU188855), *Calomicrolaimus* sp. (HF9.SSU20251), *Odontophora* sp. (HF1.SSU779114) and *Sabatieria* sp. (TF6.SSU48167).

The original output from the QIIME analysis included 145 OTUs assigned to the phylum Nematoda. Four of them were incorrectly placed among nematodes due to errors in the reference database derived from SILVA—they group with Arthropoda (HE1.SSU866120, HE6.SSU382930, HF6.SSU331569) and Phoronida (TS6.SSU559982) in all other analyses and were excluded. Two more sequences cluster with nematodes but appear to have long insertions within conserved regions (HE6.SSU358113 and TF5.SSU411806). Both of them were found only in one sample each, further supporting the idea that they are derived from an erroneous amplification product, and were removed from any further analysis. The final list of nematode OTUs includes 139 query sequences.

### 2.5. Taxonomy assignment of nematode OTUs using alignment-based methods

All 139 nematode OTUs were manually analysed using BLASTN 2.5.0+ [17] against the nucleotide collection of the NCBI database (http://blast.ncbi.nlm.nih.gov/Blast.cgi) on 22 August 2016 with the following settings: *optimize for highly similar sequences* (megablast), *exclude uncultured/environmental sample sequences*, *max target sequences—100*, sorted by max score. Closest matches were evaluated. If the top match sequence was still labelled as ‘uncultured’, ‘unidentified’ or ‘environmental’, the next best match was evaluated. Assignment to the family level was based on the top hit with at least a 90% identity score, with 100% sequence cover, as well as assignment consistency (e.g. top hits assigned to the same family). It is based on a study [42] which defines 99% identity of the 18S rRNA gene equal to species, 96.5% to genera, 90% to families and 84% as equivalent to orders (or 1%, 3.5%, 10% and 16% difference per position) using single linkage clustering. The chosen threshold was further confirmed by Holovachov [43], who found that a 90% identity score is usually sufficient to assign OTUs (based on V1--V2 region of 18S rRNA) of marine nematodes to families.

The LCAClassifier function of the CREST web server (http://apps.cbu.uib.no/crest) was used to assign taxonomy to 139 OTUs using the built-in silvamod database [16] on 25 August 2016. Three different scores of the LCA relative range were tested separately: 2%, 5% and 10%. The results based on the LCA range of 2% provided the highest number of identified OTUs and were retained for further analysis and comparison.

### 2.6. Taxonomy assignment of nematode OTUs using tree-based approach

According to published tests [44], the tree-based approach does not allow grouping of sequences into well-supported monophyletic clades equivalent in their taxonomic composition to nematode orders, but most of the marine nematode families are well resolved and supported. The reference sequence dataset was based on the ‘filtered’ alignment from Holovachov [44] that was updated with newly published sequences of marine nematodes. The final reference dataset is composed of 305 sequences representing the majority of marine nematode families as well as selected freshwater and terrestrial families, some species of which are known to inhabit the marine environment, plus three outgroup taxa (electronic supplementary material, table S1). The same set of sequences was used for the taxonomy placement using a phylogeny-based approach (§2.7).

The reference dataset was trimmed to the barcoding region and aligned with query sequences using the ClustalW [45] algorithm at default settings implemented in MEGA v. 7 [46]. The final alignment was 433 bases long. A phylogenetic tree was built using maximum-likelihood phylogeny inference with RAxML v. HPC2 [47] at default settings (GTR substitution model) with 1000 bootstrap replicates via the CIPRES portal [48]. Two independent analyses were performed: in the first case, all 139 query sequences (cumulative reference dataset) were aligned with the reference dataset and analysed at once; in the second case, 139 query sequences were split into 14 groups of 10 or nine (partitioned query dataset); each group was separately aligned with the complete reference dataset and analysed. This was done to verify if the number and composition of query sequences have any impact on the effectiveness of the tree-based taxonomy assignment approach. OTUs were assigned to the families when they are placed within monophyletic and highly supported clades (bootstrap support of 70% or higher [49,50]), equivalent in their composition to the family-level taxonomic category or below (subfamily, genus), following the same principles that are used when species are classified in supraspecific taxa using the results of phylogenetic analysis [51].

### 2.7. Taxonomy assignment of nematode OTUs using phylogeny-based approach

Alignments from Holovachov *et al.* [52,53] were combined together and supplemented with other sequences of marine nematodes available in GenBank. To minimize any potential errors and inconsistencies, at the tree-building stage, alignment stage and placement stage, all sequences used for generating reference alignment and the reference tree were selected to be as complete as possible, with the exception of taxa for which no alternative option was available. Secondary structure annotation was manually added to all non-annotated sequences using the JAVA-based editor 4SALE [54], and all sequences were manually aligned to maximize the apparent positional homology of nucleotides. The resulting alignment includes representatives of all families of marine nematodes for which sequence data are available, as well as selected freshwater, terrestrial and animal parasitic taxa (electronic supplementary material, table S1). The reference tree was built using RAxML ver. HPC2 [47] via the CIPRES portal [48] with maximum-likelihood inference of the partitioned dataset. The GTR nucleotide substitution model was used for non-paired sites, whereas the RNA7A [55] substitution model was used for paired sites. Bootstrap maximum-likelihood analysis was performed using the rapid bootstrapping option with 1000 iterations.

Query sequences were aligned to a fixed reference alignment (created in the previous step) using either mothur v. 1.36.1 [56] or PaPaRa [57] under default settings. Taxonomy predictions for query sequences were than generated with the EPA [28] implemented in RAxML [47] using the following command: *raxmlHPC-PTHREADS -T 2 -f v -s alignment_file -t reference_tree -m GTRCAT -n output.* Taxonomic assignments to family-level taxonomic categories were based either on high likelihood (above the 95% threshold) of a single placement, or on high cumulative likelihood (above the 95% threshold) of multiple placements, all of which are within a single strongly supported monophyletic clade equal to a family (see §4.4 for explanation). The 95% similarity threshold is the default used by the EPA.

### 2.8. Image processing

Trees were visualized using FigTree [58] and iTOL [59]. All clades with bootstrap support lower than 70% were collapsed in the final illustrations. Secondary structure of the barcoding region of 18S rRNA (electronic supplementary material, figure S1) was visualized using VARNA [60].

## 3. Results

### 3.1. Morphology-based analysis of samples

The nematode fauna in the coarse sand from the Hållö site included 107 different nematode species belonging to 86 genera and 33 families (electronic supplementary material, table S2). Of these, floatation using MgCl_2_ recovered 88 species from 73 genera and 26 families, while floatation using H_2_O recovered 101 species from 83 genera and 33 families. The differences in nematode fauna extracted using two variations of the same method are limited to rare species of different size classes (from less than 0.5 mm to over 2 mm). Relative abundance of these rare species does not exceed 0.14% (0.01–0.14%, with an average of 0.03%). The list of nematodes from the Hållö site includes four species new to the fauna of Sweden (*Bolbonema brevicolle, Bradylaimus pellita, Desmodora granulata* and *Odontophora villoti*) and five species new to science (from the genera *Adelphos, Paramesonchium, Leptolaimus* and *Diplopeltoides*).

Mud sediments from the Telekabeln site were inhabited by 113 different nematode species, belonging to 77 genera and 33 families (electronic supplementary material, table S3). Of these, siphoning recovered 81 species from 62 genera and 29 families, while floatation using H_2_O recovered 102 species from 70 genera and 32 families. The differences in nematode fauna extracted using two different methods include both rare and uncommon species of various size classes (from less than 0.5 mm to over 2 mm). The relative abundance of these rare species does not exceed 2.02% (0.01–2.02%, with an average of 0.29%). The list of nematodes from the Telekabeln samples includes seven species new to the fauna of Sweden (*Campylaimus rimatus, C. amphidialis, C. tkatchevi, C. orientalis, Diplopeltoides asetosus, D. linkei* and *D. nudus*) and one species new to science (from the genus *Diplopeltoides*).

### 3.2. Taxonomy placement of OTUs using alignment-based approaches

#### 3.2.1. BLASTN

Out of 139 queried OTUs, 52 could be assigned to family-level categories based on the following criteria: 90% or more identity score and 100% sequence cover, as well as assignment consistency (electronic supplementary material, table S4). In one case, BLASTN search produced conflicting results—two top hits with the same identity score and sequence cover that belonged to different families, but still falling within the threshold limit. This is the barcode TF1.SSU676746 that showed 90% identity and 100% sequence cover to *Haliplectus* sp. (family Haliplectidae) and *Prodesmodora* sp. (family Desmodoridae). It was considered unassigned. Similar examples were seen in BLASTN results of other OTUs that did not reach the threshold. These examples show that considering only one top hit when assigning taxonomy to query OTUs using alignment-based approaches may sometimes lead to questionable or dubious identification.

#### 3.2.2. CREST

Only 26 out of 139 queried OTUs were assigned to families using LCAClassifier of CREST under default parameters (electronic supplementary material, table S5) and following built-in classification. In two cases, OTUs were placed outside Nematoda: HE3.SSU118424 was placed within Copepoda (phylum Arthropoda) and TS1.SSU284163 was placed in Scolecida (phylum Annelida). The first OTU was positively assigned to the family Oxystominidae (phylum Nematoda) using tree-based and phylogeny-based approaches (see §3.3 and 3.4); the second OTU was unassigned in all other analyses.

### 3.3. Taxonomy placement of OTUs using tree-based approaches

#### 3.3.1. Cumulative query dataset

**Figure 1.**
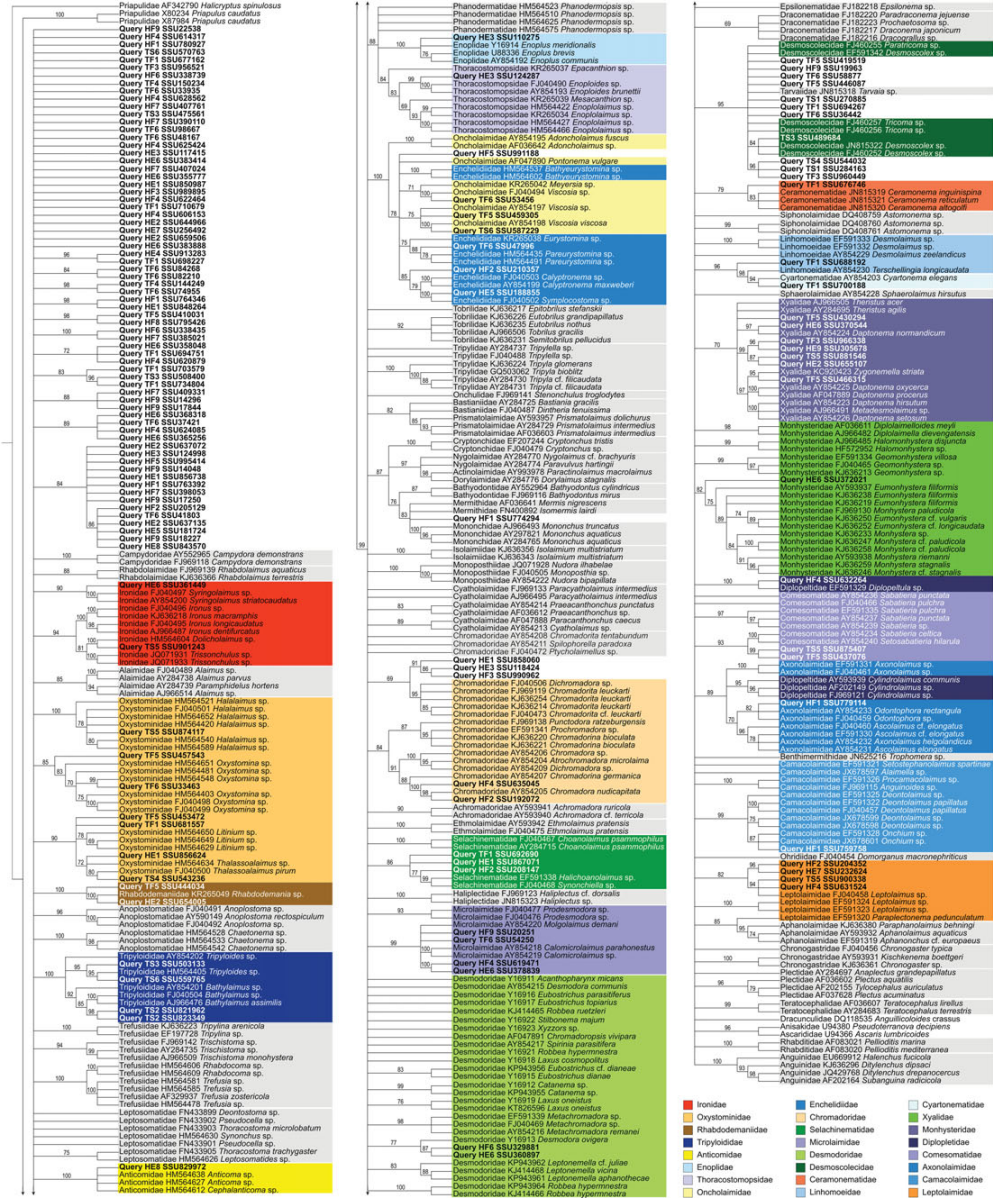
Phylogram based on tree-based taxonomy assignment approach using a complete query dataset. Families that include positively assigned OTUs are colour-coded; remaining reference taxa are shaded in grey.

Tree-based taxonomy assignment of the cumulative query dataset produced 54 well-supported placements (figure 1; electronic supplementary material, table S6) that fulfilled the following criteria: OTU must cluster within the monophyletic clade that has high bootstrap support (greater than or equal to 70%) and is at or below family level. The remaining 85 OTUs could not be placed in clades satisfying these criteria, and are thus treated as unidentified.

#### 3.3.2. Partitioned query dataset

The results of taxonomic assignment using a tree-based approach of the partitioned query dataset produced somewhat different results compared to the cumulative query dataset—67 OTUs were placed in monophyletic clades equivalent to family-level categories with sufficient support (electronic supplementary material, table S6). Of these, taxonomic placement of only 47 OTUs matched the identification produced using the cumulative query dataset, and identifications of 20 OTUs were new. Seven OTUs were not assigned using a partitioned query dataset but were positively identified using a cumulative query dataset.

### 3.4. Taxonomy placement of OTUs using phylogeny-based approaches

#### 3.4.1. EPA/mothur

**Figure 2.**
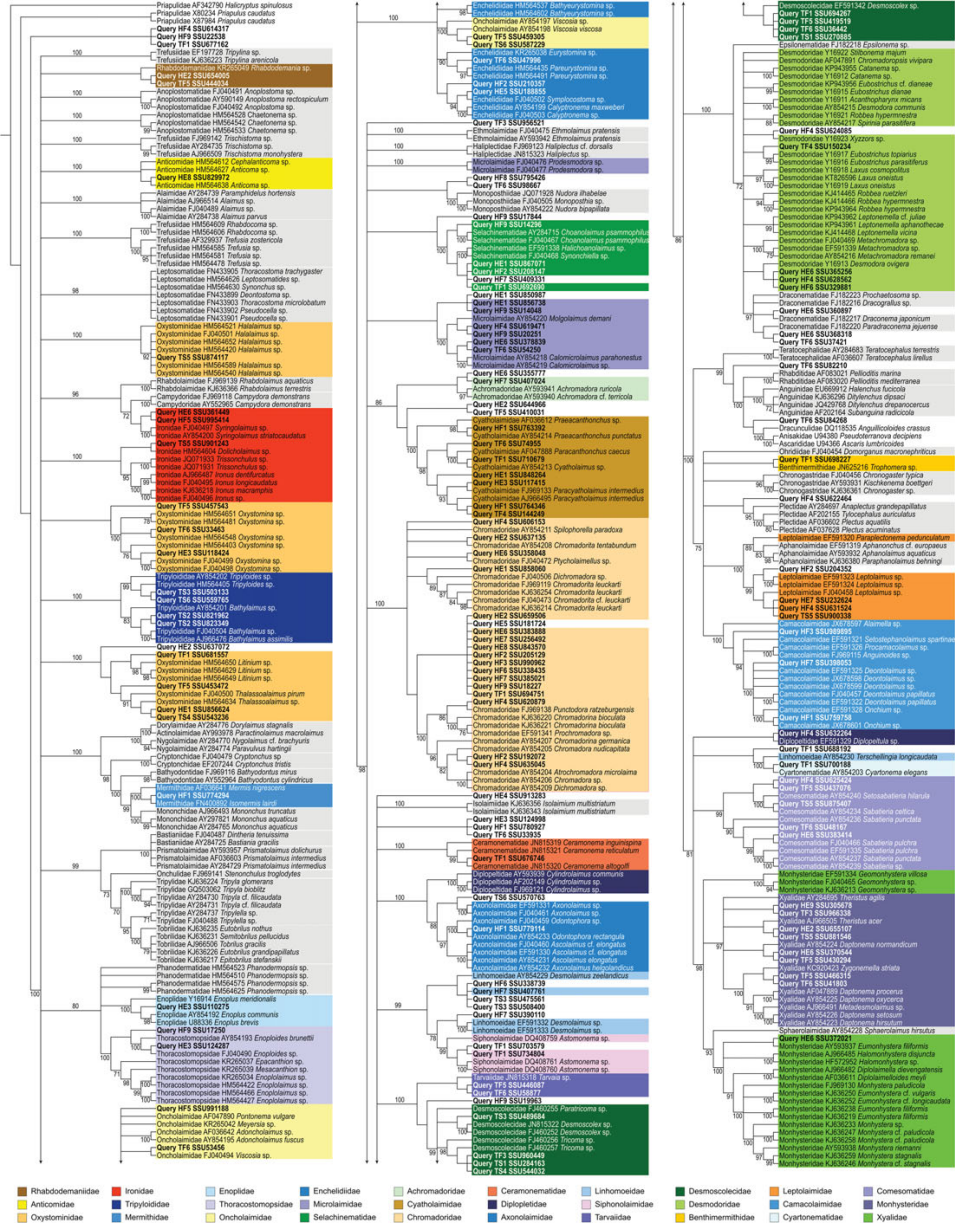
Phylogram based on phylogeny-based taxonomy assignment approach. Families that include positively assigned OTUs are colour-coded; remaining reference taxa are shaded in grey.

Phylogeny-based taxonomy assignment using mothur-based alignment and the EPA produced 105 well-supported placements with single or accumulated likelihood of 0.95 or more (figure 2; electronic supplementary material, table S7). There are ten additional cases when the positive identity cannot be attained because OTUs are placed either within a paraphyletic assemblage (family Desmodoridae or Linhomoeidae) or closely related monophyletic clade (Draconematidae or Siphonolaimidae, respectively).

#### 3.4.2. EPA/PaPaRa

The results produced using PaPaRa-based alignment and the EPA are exactly the same as those obtained using mothur-based alignment and described in §3.4.1 (electronic supplementary material, table S7), even though visual comparison of alignments produced by mothur and by PaPaRa revealed some differences.

### 3.5. Comparison of different taxonomy assignment approaches

Among the three different taxonomy assignment approaches tested (each with two variations), the EPA (both variations) placed the largest number of query OTUs into family-level taxonomic categories (105 out of 139), while CREST implementation of the alignment-based assignment approach was the least efficient (26 out of 139). Despite such a broad success rate, the family identifications were in most cases congruent among different approaches—most of the identified OTUs were assigned to the same families (electronic supplementary material, table S8), with the following exceptions:

(i) HF1.SSU759758 was placed in the family Camacolaimidae using tree-based and phylogeny-based approaches, in the family Leptolaimidae using CREST and unassigned using BLASTN;
(ii) HF5.SSU995414 was placed in the family Rhabdolaimidae using BLASTN, in the family Ironidae using CREST and both variations of the EPA, and unassigned using the tree-based approach;
(iii) TF1.SSU698227 was placed in the family Teratocephalidae using BLASTN and in the family Benthimermithidae using both variations of the EPA, and unassigned in other cases;
(iv) TF1.SSU700188 was placed in the family Linhomoeidae using BLASTN, in the family Cyartonematidae using tree-based and phylogeny-based approaches, and unassigned using CREST;
(v) TF6.SSU47996 was placed in the family Oncholaimidae using BLASTN and in the family Enchelidiidae in all other cases.

### 3.6. Comparison between barcode-based and morphology-based identification

**Figure 3.**
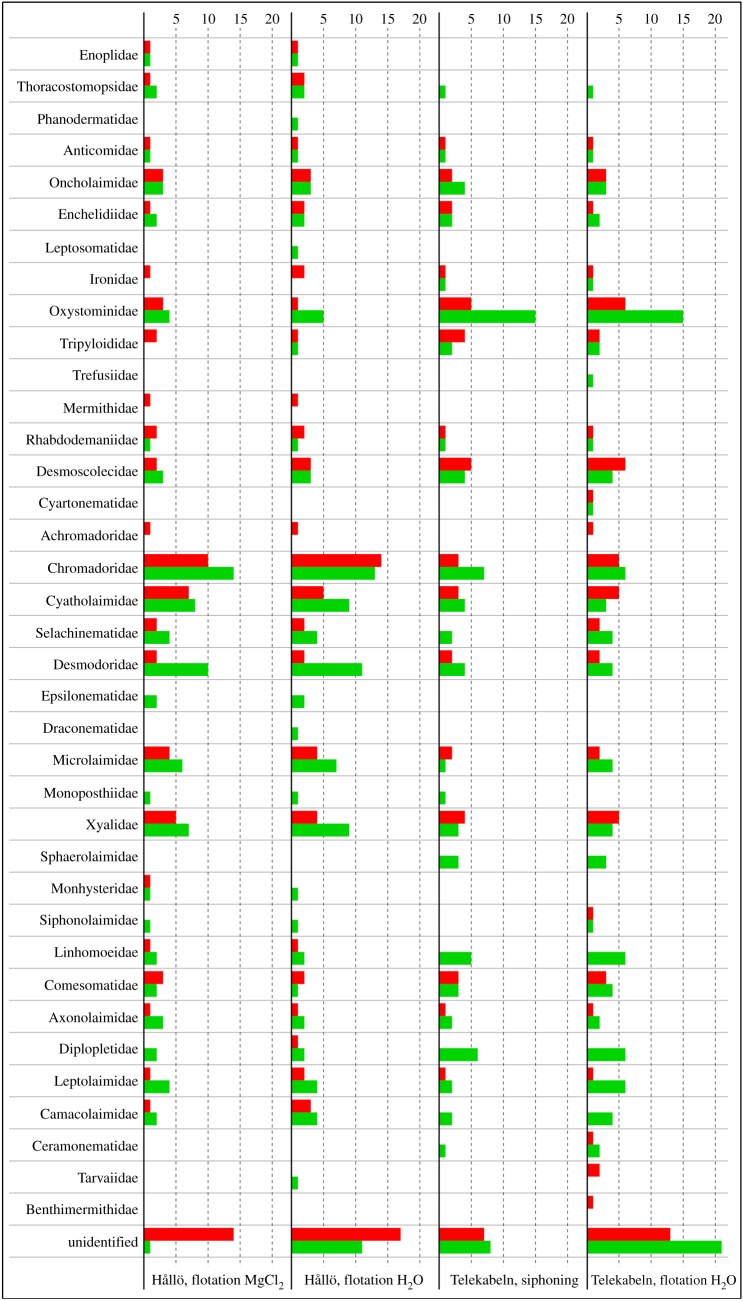
Comparison of the total number of taxa identified using phylogeny-based taxonomy assignment approach (OTUs, red) and morphology-based identification (morphospecies, green) for each nematode family in each sample (sampling site/extraction method) based on table S9 in the electronic supplementary material (excluding families without reference sequence data).

The EPA (phylogeny-based approach) provided the largest number of positively identified OTUs and will be compared with the faunistic lists created by identifying nematode specimens using morphological characters. As species-level identification cannot be achieved for most of the OTUs, the results of barcode-based and morphology-based identifications can only be compared as the number of identified OTUs/morphospecies per family (figure 3; electronic supplementary material, table S9). Among families with available reference sequences, barcode-based identification failed to identify the families Phanodermatidae, Leptosomatidae, Trefusiidae, Epsilonematidae, Draconematidae, Monoposthiidae and Sphaerolaimidae. One of the likely explanations is that nematodes from these families failed to amplify or that barcode sequences produced during sequencing failed quality control.

On the other hand, barcode-based identification also uncovered several taxa that were overlooked during morphology-based identification, such as the families Achromadoridae, Mermithidae and Benthimermithidae—the last two are internal parasites of invertebrates during part of their life cycle and were most probably overlooked, because examination of the meiofauna for internal parasites was not attempted. In all other cases, the efficiency of either barcode-based or morphology-based identification varied considerably, even within the same taxon across different samples (figure 3). Nevertheless, the Pearson correlation coefficient revealed moderate positive correlation (*ρ *=* *0.7296967138) between the number of assigned OTUs and identified morphospecies in each family/extraction/sample (electronic supplementary material, figure S2).

## 4. Discussion

### 4.1. General notes

Three different taxonomy assignment approaches (with two modifications each) tested in this project provide some variation in the number of positively identified OTUs; however, the assigned identities of those OTUs that were identified were consistent with very few exceptions (§3.5). These discrepancies can possibly be caused by several different factors. Placement of one of the OTUs (HF1.SSU759758) either in the family Camacolaimidae (tree-based and phylogeny-based approaches) or in the family Leptolaimidae (CREST) is probably a result of outdated classification of the phylum Nematoda used in the SILVA-derived reference database implemented in CREST, compared to the nematode classification used in WoRMS and in this publication (§4.6). Conflicting results of the assignment of TF1.SSU698227 either in the family Teratocephalidae (BLASTN) or in the family Benthimermithidae (EPA) can be due to poor representation of the reference dataset in this part of the nematode tree. The remaining conflicting placements of HF5.SSU995414 (Rhabdolaimidae versus Ironidae), TF1.SSU700188 (Linhomoeidae versus Cyartonematidae) and TF6.SSU47996 (Oncholaimidae versus Enchelidiidae) are possibly caused by the fact that the overall sequence similarity used by BLASTN does not necessarily reflect common phylogenetic history, which is the basis of the tree-based and phylogeny-based assignment approaches. Differences in the individual success rates of each taxonomy assignment approach will be discussed in §4.2–4.4.

### 4.2. Alignment-based approach

Alignment-based approaches tested in this publication include manual analysis using BLASTN 2.5.0+ [17] against the nucleotide collection of the NCBI database and the LCAClassifier function of the CREST against the built-in silvamod database [16]. Both tested approaches have their own advantages and disadvantages. NCBI implementation of BLASTN allows visual examination of multiple top hits in the output and individual evaluation of these top hits, manual application of the variable similarity threshold if it has been predetermined empirically and, if necessary, correction of classification. Taxonomy assignment using CREST is less flexible and has the following limitations: (i) similarity thresholds used in CREST are based on the prokaryotic 16S rRNA analysis and do not account for the differences in the variability of rRNA within and between different taxa [43]; (ii) classification of the phylum Nematoda that is used in the CREST database is different from the most recent and widely accepted classification scheme published in WoRMS; and (iii) results of the taxonomy assignment in the output files cannot be verified and, if necessary, updated.

Strictly speaking, alignment-based assignment approaches should not be used to place OTUs to supraspecific taxa without critical evaluation of the results. First of all, similarity scores used in BLASTN search results do not reflect phylogenetic affinities of analysed taxa, and do not account for the fact that the level of variability of the 5′ barcoding region of 18S rRNA (electronic supplementary material, figure S1) is different in various nematode taxa [43]. Too narrow similarity thresholds can exclude potentially identifiable sequences, while too broad thresholds can lead to misidentifications. Dell'Anno *et al*. [4] is an example where broad similarity threshold resulted in incorrect assignment of several nematode OTUs from deep-sea samples to nematode species known to inhabit freshwater and soil and never found in the marine environment (e. g. *Anaplectus porosus, Anaplectus* sp., *Pakira orae* and *Tylolaimophorus* sp.).

### 4.3. Tree-based approach

Phylogenetic hypotheses used to infer relationships of taxa are usually thoroughly described and rigorously evaluated, and undergo comparison and testing using different alignment and tree-building algorithms. Phylogenetic trees used to identify unknown barcodes are less so [20,21]. Barcodes are by definition relatively short in length, hypervariable sites flanked by conserved regions. Hypervariable domains V1 and V2, which are part of the barcoding region of the 18S rRNA used in this publication, are the culprit that causes poor alignment and hence has negative effect on the quality of the resulting phylogeny. Different alignment and phylogeny-inference algorithms may provide competing phylogenetic hypotheses [44] and, as a result, different placements of OTUs in the phylogram. Taxon composition and sequence quality (exclusion of incorrectly identified species, low quality and short sequences) of the reference dataset is also crucial [44], as it determines which taxa can be identified and which taxa cannot. Even the number and composition of OTUs have strong effect on the final phylogenetic tree and, as a result, on the outcome of the taxonomy assignment, as shown in §3.3. The latter is caused by the need to align de novo the combined datasets that include reference and query sequences—the presence of unidentified sequencing errors among query OTUs can have a negative effect on the alignment and phylogeny inference, even if all reference sequences are of high quality. This effect is global, i.e. by affecting the entire alignment and tree topology and bootstrap, erroneous sequences can potentially cause other OTUs to be misidentified or unidentified. In conclusion, successful use of tree-based approaches to assign taxonomy to OTUs is highly dependent not only on the quality and completeness of the reference dataset and alignment and phylogeny inference algorithms, but also on the quality and diversity of query sequences.

### 4.4. Phylogeny-based approach

Phylogeny-based approaches allow the estimation of the most likely position of each OTU within the constrained phylogenetic tree, estimation of the rank of its taxonomic placement in supraspecific categories if these are well resolved and supported in the reference phylogeny, and can even work with paraphyletic taxa. Moreover, because the reference alignment and reference phylogeny are constrained during phylogeny-based taxonomy assignment procedures, the quality of query sequences has no impact on the result, i.e. the presence of erroneous sequences among query OTUs (chimaeras) has no effect on the identification of other query OTUs. The outcome of the analysis solely depends on the quality of the reference alignment and reference phylogeny. Even minor differences in the alignment of OTUs against the reference alignment noted above (§3.4.2) had no effect on the results. An additional advantage of the phylogeny-based taxonomy assignment approach implemented in the EPA is the possibility to use cumulative likelihood scores when assigning taxa to clades equivalent to supraspecific taxonomic categories (electronic supplementary material, figure S3).

### 4.5. Metabarcoding versus morphology-based identification

Morphology-based identification procedures are strongly biased by the expertise and experience of the researcher performing the identification, as well as the state of the knowledge on the diversity of particular groups of nematodes. Metabarcoding, on the other hand, should be able to better estimate the diversity of poorly known groups of nematodes, or groups for which taxonomic expertise is not available at the moment, as well as unidentifiable specimens (eggs, juveniles, damaged specimens, etc.). Moreover, metabarcoding can reveal taxa that are physically hidden and cannot be observed by the researcher during sorting and identification, such as internal parasites—similarly to the results obtained by Lindeque *et al*. [39], barcode-based identification revealed the presence of endoparasitic nematodes from the families Mermithidae and Benthimermithidae in our samples. They had been overlooked during morphology-based identification, probably being juveniles within bodies of other invertebrates.

The number of OTUs identified by metabarcoding is strongly influenced by the clustering procedures of the raw sequence data and, depending on the threshold used, will give different results. Assuming that the OTUs produced through metabarcoding are equivalent to currently recognized morphospecies, the only reason it would not be able to correctly estimate the number of species in the sample is if there are issues with amplification of the barcoding gene. The genus *Halalaimus* is a good example of a problematic taxon in this case—only one *Halalaimus* OTU (TS5.SSU874117) was recovered with metabarcoding, and only from the Telekabeln site. Morphology-based identification recovered at least two different *Halalaimus* species in the Hållö site and more than eight species in the Telekabeln site, some of which were relatively common. GenBank hosts a number of *Halalaimus* sequences, confirming that the genus is sufficiently diverse genetically, and that our single *Halalaimus* OTU is unlikely to encompass multiple morphospecies, but is rather a result of amplification problems.

### 4.6. Reference databases

Taxonomy assignment procedures described in the literature [16,41] often rely on various releases of the SILVA database [33], which in turn is based on the sequence data published in GenBank or EMBL. These databases can be ‘built-in’ (CREST), and completely inaccessible for the user, or ‘pre-made’ and hard to modify (QIIME). The presence of erroneously identified sequences of nematodes and other organisms in GenBank and SILVA databases has been mentioned multiple times [43,44,61,62]. If the reference database is not checked for errors prior to the analysis, the results produced by any taxonomy assignment algorithm should be evaluated using available data on geographical or ecological distribution of species, in order to avoid mistakes.

As mentioned earlier, the SILVA database in itself does not always follow the most recent accepted classification for certain groups of organisms. As a result, placing some of the OTUs into nematode families based on the SILVA classification turned out to be incorrect. For example, genera *Paracyatholaimus* and *Preacanthonchus* were placed in the family Chromadoridae using QIIME, while they do belong to the family Cyatholaimidae. Similar examples are: *Enoploides* placed in Enoplidae instead of Thoracostomopsidae, *Calyptronema* in Oncholaimidae instead of Enchelidiidae, *Achromadora* in Chromadoridae instead of Achromadoridae, *Camacolaimus* in Leptolaimidae instead of Camacolaimidae, and some others. Output from CREST [162] only gives the name of the supraspecific taxon for those cases where a query OTU cannot be identified to species level. This prevents proper evaluation of the assignment results and correction of assignments derived from an erroneous reference sequence or incorrect classification. We do not expect any database to be able to quickly reflect changes in nematode classification, but we expect end users of these databases to be aware of the need to verify and, if necessary, to update the output of any taxonomy assignment procedure that they may use.

Another disadvantage of taxonomy assignment software that uses built-in databases and offers only top-pick assignments in the output files (QIIME, CREST) is that a substantial number of OTUs are matched with environmental samples, labelled in such databases with the words ‘environmental’ (e.g. ‘environmental sample’), ‘uncultured’ (e.g. ‘uncultured eukaryote’) and ‘unidentified’ (‘unidentified nematode’). They themselves are OTUs generated during previous metabarcoding projects and identified not by looking at actual morphological vouchers but by using one of the multiple taxonomy assignment methods. Moreover, by giving only one top ‘hit’ assignment, such software eliminates the possibility to verify if the ‘second best’ hit is based on sequence data from the physically observed and identified morphological voucher, and its similarity score, preventing the researcher from making educated decisions on the taxonomic identity of an OTU.

## 5. Conclusion and future prospects

The identification of OTUs is obviously a key step in metabarcoding and it is essential that the most effective method is used (as opposed to the fastest or simplest). Ideally, the barcode sequences should be assigned taxonomic names that provide a link to all biological knowledge that may exist in relation to the organism. Misidentification will compromise the results, for example, in studies of biogeography, community structure, habitat state or the presence of certain important species (invasive, rare, indicators, etc.).

Identification of OTUs should be at the appropriate taxonomic level, which is determined by the available reference sequences and the purpose of the study. In the case of marine nematodes, we were able to assign our barcode sequences to family-level taxa to a high degree despite the very incomplete reference database. The relevance of family-level metabarcoding data in ecological studies remains poorly tested and requires extensive comparison with data obtained using classical approaches.

The full potential of metabarcoding is realized when sequences are identified to species level. This conveys the most information and permits more robust inferences. A prerequisite for this is taxonomic groundwork in the form of complete curated reference databases with sequences of reliably identified specimens.

We found the phylogeny-based taxonomy assignment approach to be the most efficient and the least error-prone. The alignment-based approach is less reliable because the similarity thresholds it depends on do not account for inter- and intra-taxon variations in barcode sequence, while tree-based approaches can be affected by the quality of the input OTU data. If phylogeny-based taxonomy assignment methods become widely used in nematode metabarcoding, it is imperative to create and maintain high-quality reference alignments and reference phylogenetic trees to be used by researchers worldwide.

## Supplementary Material

Supplementary Figure 1

## Supplementary Material

Supplementary Figure 2

## Supplementary Material

Supplementary Figure 3

## Supplementary Material

Supplementary Table 1

## Supplementary Material

Supplementary Table 2

## Supplementary Material

Supplementary Table 3

## Supplementary Material

Supplementary Table 4

## Supplementary Material

Supplementary Table 5

## Supplementary Material

Supplementary Table 6

## Supplementary Material

Supplementary Table 7

## Supplementary Material

Supplementary Table 8

## Supplementary Material

Supplementary Table 9

## Supplementary Material

Supplementary Data 1

## Data accessibility

The data supporting this article are available in the electronic supplementary material.
