# Supplementary material for: Taxonomy assignment approach determines the efficiency of identification of OTUs in marine nematodes
Source: R Soc Open Sci. 2017 Aug 16;4(8):170315. doi: 10.1098/rsos.170315 (PMC5579096; doi:10.1098/rsos.170315)
Supplement: Supplementary Figure 1 [file rsos170315supp1.pdf]

Supplementary file for the article:

Holovachov O, Haenel Q, Bourlat SJ, Jondelius U. Taxonomy assignment approach determines the efficiency of identification of OTUs in marine nematodes. *Royal Society Open Science.*

Supplementary Figure 1. Barcoding region used in this study – generalized secondary structure model of the 90% consensus sequence (excluding rare insertions) based on combined data from secondary-structure-based alignment of reference sequences and mothur-based alignment of OTU sequences (444 sequences in total). Helices (6-10, 10/e1, 11-14) are according to [63]. Variable regions V1 and V2 are labelled according to [64].

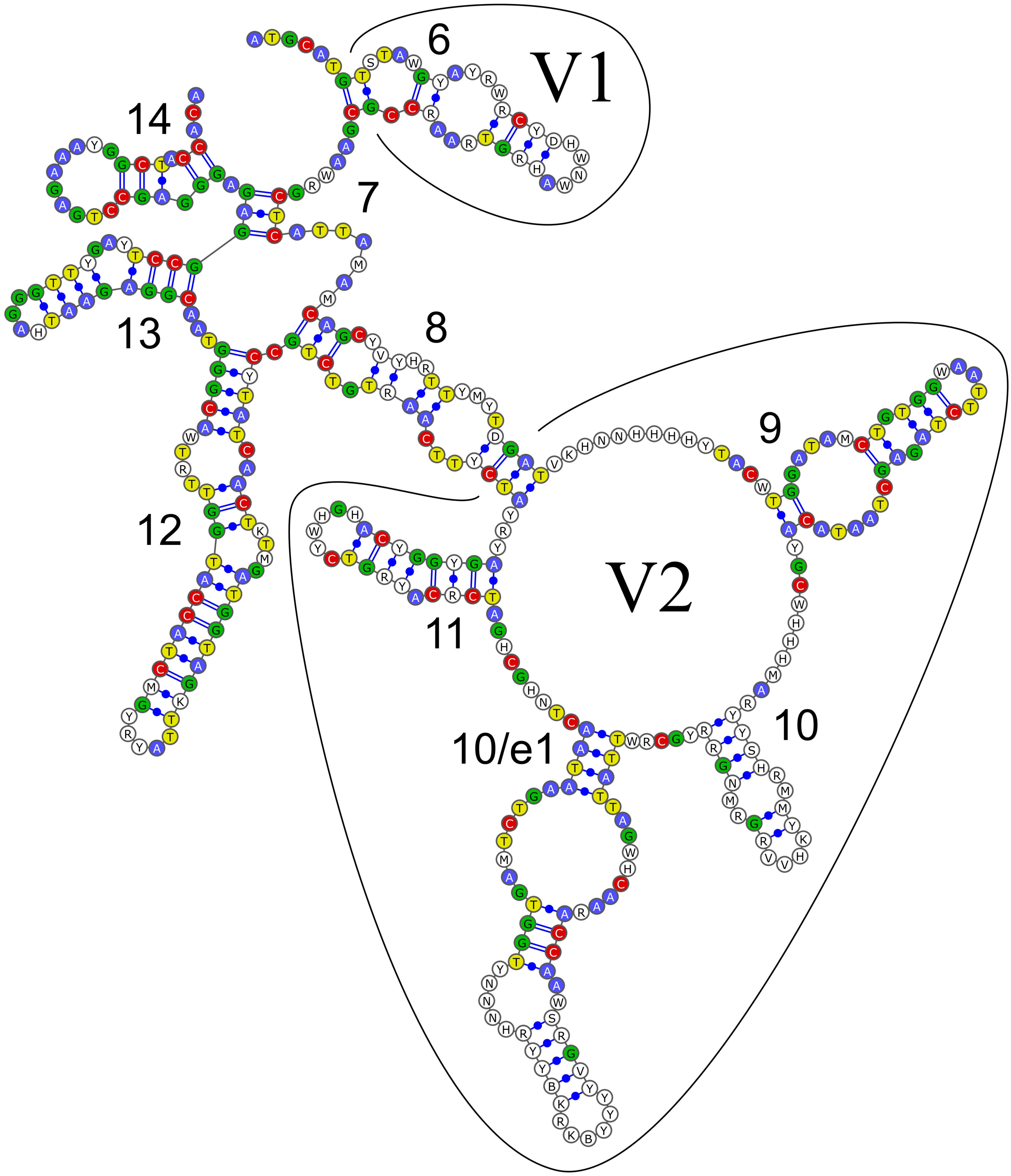

References

63. Wuyts J, Van de Peer Y,Winkelmans T, DeWachter R. 2002 The European database on small subunit ribosomal RNA. *Nucleic Acids Res.* **30**, 183–185. (doi:10.1093/nar/30.1.183)

64. Neefs JM, Van de Peer Y, Hendriks L, DeWachter R. 1990 Compilation of small ribosomal subunit RNA sequences. *Nucleic Acids Res.* **18**, 2237–2317. (doi:10.1093/nar/18.suppl.2237)
