# Supplementary material for: Taxonomy assignment approach determines the efficiency of identification of OTUs in marine nematodes
Source: R Soc Open Sci. 2017 Aug 16;4(8):170315. doi: 10.1098/rsos.170315 (PMC5579096; doi:10.1098/rsos.170315)

Supplementary file for the article:

Holovachov O, Haenel Q, Bourlat SJ, Jondelius U. Taxonomy assignment approach determines the efficiency of identification of OTUs in marine nematodes. *Royal Society Open Science.*

Supplementary Figure 2. Graphical comparison of the number of taxa identified using morphology-based identification (X axis) *versus* phylogeny-based taxonomy assignment approach (Y axis) for each nematode family in each sample (sampling site/extraction method) based on Supplementary Table 9 (excluding families without reference sequence data). Size of "bubbles" corresponds to the total number of families that show same result (for all sites and extraction methods).

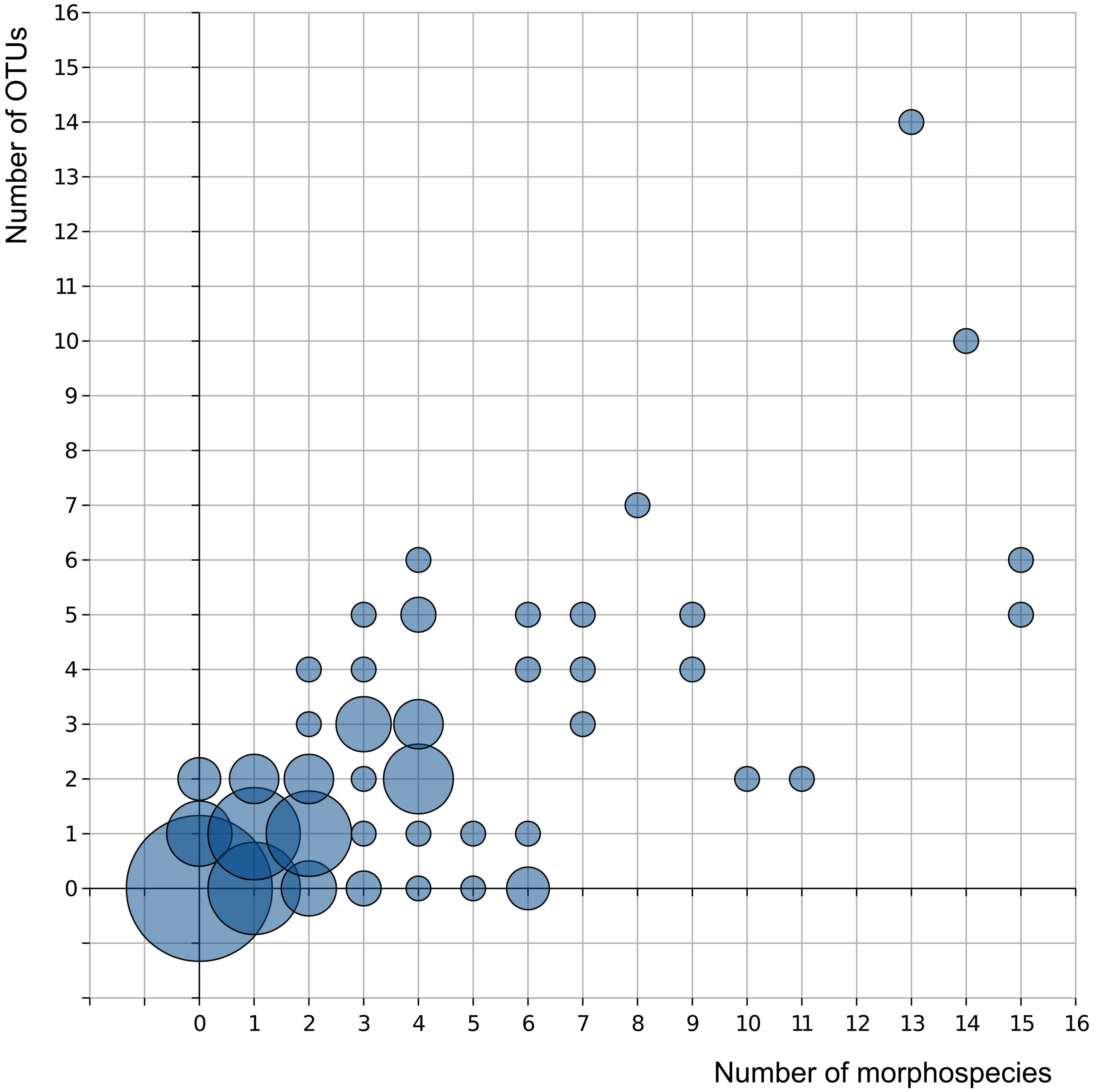

Supplement: Supplementary Figure 2 [file rsos170315supp2.pdf]
