# Supplementary material for: Taxonomy assignment approach determines the efficiency of identification of OTUs in marine nematodes
Source: R Soc Open Sci. 2017 Aug 16;4(8):170315. doi: 10.1098/rsos.170315 (PMC5579096; doi:10.1098/rsos.170315)

Supplementary file for the article:

Holovachov O, Haenel Q, Bourlat SJ, Jondelius U. Taxonomy assignment approach determines the efficiency of identification of OTUs in marine nematodes. *Royal Society Open Science.*

Supplementary Figure 3. Examples of cumulative placement of two different OTUs within the family Thoracostomopsidae that provide reliable identification of OTUs to the family-level taxonomic categories (branch length not representative). A: Part of the reference phylogeny with labelled clades (*i463-i477*) and bootstrap support. B: Placements of HE3.SSU124287 in clades *i464* (probability 0.333433), *i465* (probability 0.333132) and *i466* (probability 0.333435) gives cumulative probability higher than the required threshold of 0.95. C: Placements of HF9.SSU17250 in clades *i464* (probability 0.924697) and *i466* (probability 0.037675) gives cumulative probability higher than the required threshold of 0.95.

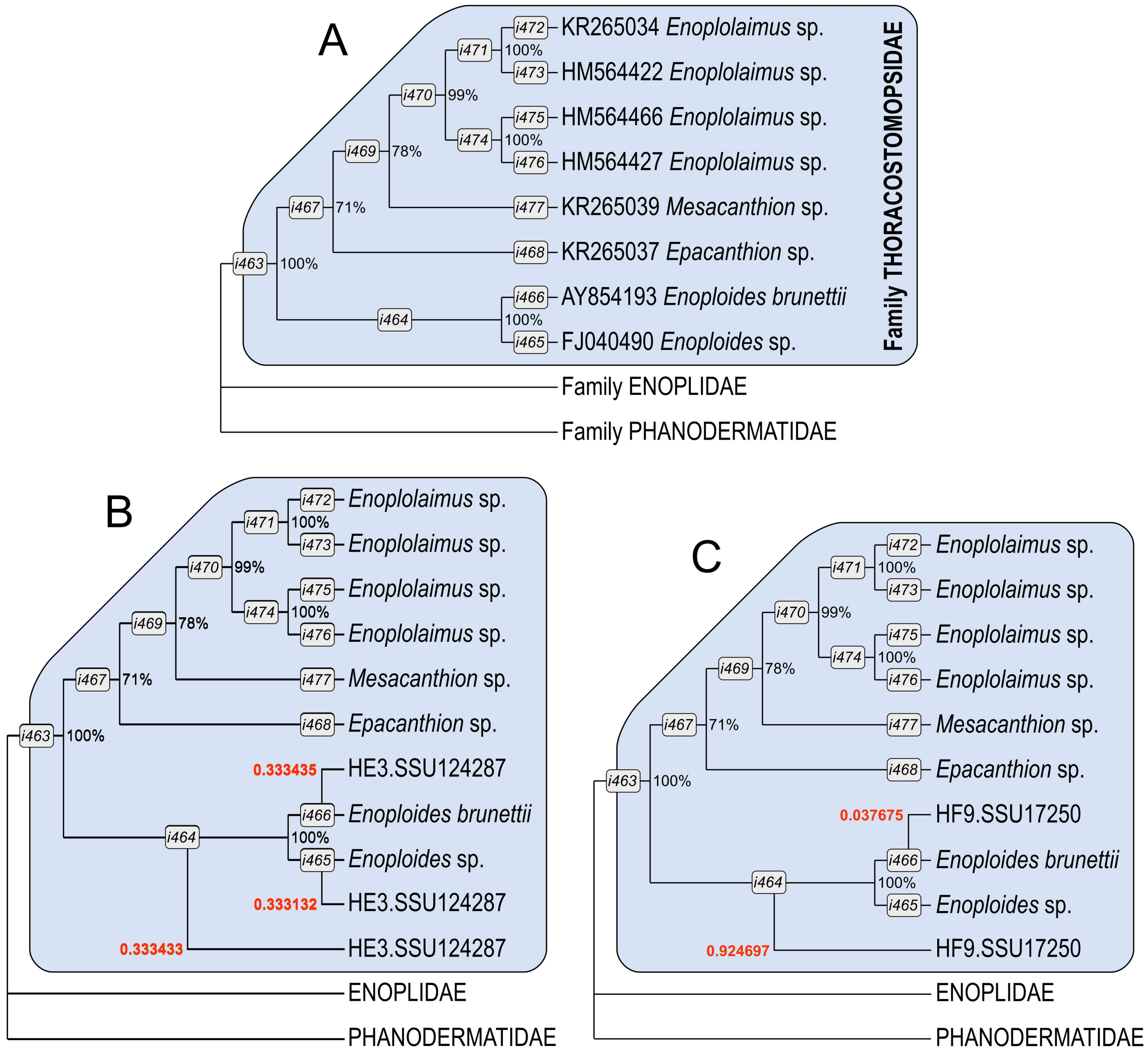

Supplement: Supplementary Figure 3 [file rsos170315supp3.pdf]
