# Supplementary material for: Taxonomy assignment approach determines the efficiency of identification of OTUs in marine nematodes
Source: R Soc Open Sci. 2017 Aug 16;4(8):170315. doi: 10.1098/rsos.170315 (PMC5579096; doi:10.1098/rsos.170315)
Supplement: Supplementary Table 1 [file rsos170315supp4.pdf]

Supplementary file for the article:

Holovachov O, Haenel Q, Bourlat SJ, Jondelius U. Taxonomy assignment approach determines the efficiency of identification of OTUs in marine nematodes. *Royal Society Open Science.*

Supplementary Table 1. GenBank accession numbers and classification of sequences used in reference datasets for the tree-based and phylogeny-based taxonomy assignment algorithms.

	**Acc. number**	**Family**	**Genus**	**Species**
1.	AF202164	Anguinidae	*Subanguina*	*radicicola*
2.	EU669912	Anguinidae	*Halenchus*	*fucicola*
3.	JQ429768	Anguinidae	*Ditylenchus*	*drepanocercus*
4.	KJ636296	Anguinidae	*Ditylenchus*	*dipsaci*
5.	AF083020	Rhabditidae	*Pellioditis*	*mediterranea*
6.	AF083021	Rhabditidae	*Pellioditis*	*marina*
7.	U94366	Ascarididae	*Ascaris*	*lumbricoides*
8.	DQ118535	Dracunculidae	*Anguillicoloides*	*crassus*
9.	U94380	Anisakidae	*Pseudoterranova*	*decipiens*
10.	AY284683	Teratocephalidae	*Teratocephalus*	*terrestris*
11.	AF036607	Teratocephalidae	*Teratocephalus*	*lirellus*
12.	AF202155	Plectidae	*Tylocephalus*	*auriculatus*
13.	AF037628	Plectidae	*Plectus*	*acuminatus*
14.	AF036602	Plectidae	*Plectus*	*aquatilis*
15.	AY284697	Plectidae	*Anaplectus*	*grandepapillatus*
16.	AY593931	Chronogastridae	*Kischkenema*	*boettgeri*
17.	FJ040456	Chronogastridae	*Chronogaster*	*typica*
18.	KJ636361	Chronogastridae	*Chronogaster*	sp.
19.	EF591319	Aphanolaimidae	*Aphanonchus*	cf. *europaeus*
20.	AY593932	Aphanolaimidae	*Aphanolaimus*	*aquaticus*
21.	KJ636380	Aphanolaimidae	*Paraphanolaimus*	*behningi*
22.	EF591320	Leptolaimidae	*Paraplectonema*	*pedunculatum*
23.	EF591323	Leptolaimidae	*Leptolaimus*	sp.
24.	EF591324	Leptolaimidae	*Leptolaimus*	sp.
25.	FJ040458	Leptolaimidae	*Leptolaimus*	sp.
26.	FJ040454	Ohridiidae	*Domorganus*	*macronephriticus*
27.	EF591321	Camacolaimidae	*Setostephanolaimus*	*spartinae*
28.	JX678597	Camacolaimidae	*Alaimella*	sp.
29.	FJ969115	Camacolaimidae	*Anguinoides*	sp.
30.	JX678601	Camacolaimidae	*Onchium*	sp.
31.	EF591328	Camacolaimidae	*Onchium*	sp.
32.	EF591322	Camacolaimidae	*Deontolaimus*	*papillatus*
33.	FJ040457	Camacolaimidae	*Deontolaimus*	*papillatus*

	Acc. number	Family	Genus	Species
34.	JX678599	Camacolaimidae	*Deontolaimus*	sp.
35.	JX678598	Camacolaimidae	*Deontolaimus*	sp.
36.	EF591325	Camacolaimidae	*Deontolaimus*	sp.
37.	EF591326	Camacolaimidae	*Procamacolaimus*	sp.
38.	JN625216	Benthimermithidae	*Trophomera*	sp.
39.	FJ040460	Axonolaimidae	*Ascolaimus*	cf. *elongatus*
40.	EF591330	Axonolaimidae	*Ascolaimus*	cf. *elongatus*
41.	AY854231	Axonolaimidae	*Ascolaimus*	*elongatus*
42.	AY854232	Axonolaimidae	*Axonolaimus*	*helgolandicus*
43.	EF591331	Axonolaimidae	*Axonolaimus*	sp.
44.	FJ040461	Axonolaimidae	*Axonolaimus*	sp.
45.	AY854233	Axonolaimidae	*Odontophora*	*rectangula*
46.	FJ040459	Axonolaimidae	*Odontophora*	sp.
47.	EF591335	Comesomatidae	*Sabatieria*	*pulchra*
48.	FJ040466	Comesomatidae	*Sabatieria*	*pulchra*
49.	AY854240	Comesomatidae	*Setosabatieria*	*hilarula*
50.	AY854237	Comesomatidae	*Sabatieria*	*punctata*
51.	AY854236	Comesomatidae	*Sabatieria*	*punctata*
52.	AY854234	Comesomatidae	*Sabatieria*	*celtica*
53.	AY854239	Comesomatidae	*Sabatieria*	sp.
54.	AY593939	Diplopeltidae	*Cylindrolaimus*	*communis*
55.	FJ969121	Diplopeltidae	*Cylindrolaimus*	sp.
56.	AF202149	Diplopeltidae	*Cylindrolaimus*	sp.
57.	EF591329	Diplopeltidae	*Diplopeltula*	sp.
58.	EF591334	Monhysteridae	*Geomonhystera*	*villosa*
59.	FJ040465	Monhysteridae	*Geomonhystera*	sp.
60.	KJ636213	Monhysteridae	*Geomonhystera*	sp.
61.	HF572952	Monhysteridae	*Halomonhystera*	sp.
62.	AJ966485	Monhysteridae	*Halomonhystera*	*disjuncta*
63.	AY593938	Monhysteridae	*Monhystera*	*riemanni*
64.	FJ969130	Monhysteridae	*Monhystera*	*paludicola*
65.	KJ636247	Monhysteridae	*Monhystera*	cf. *paludicola*
66.	KJ636258	Monhysteridae	*Monhystera*	cf. *paludicola*
67.	KJ636246	Monhysteridae	*Monhystera*	cf. *stagnalis*
68.	KJ636259	Monhysteridae	*Monhystera*	*stagnalis*
69.	KJ636233	Monhysteridae	*Monhystera*	sp.
70.	KJ636250	Monhysteridae	*Eumonhystera*	cf. *vulgaris*
71.	KJ636238	Monhysteridae	*Eumonhystera*	*filiformis*
72.	AY593937	Monhysteridae	*Eumonhystera*	*filiformis*
73.	KJ636219	Monhysteridae	*Eumonhystera*	*filiformis*
74.	KJ636252	Monhysteridae	*Eumonhystera*	cf. *longicaudata*

	Acc. number	Family	Genus	Species
75.	AJ966482	Monhysteridae	*Diplolaimella*	*dievengatensis*
76.	AF036611	Monhysteridae	*Diplolaimelloides*	*meyli*
77.	AJ966505	Xyalidae	*Theristus*	*acer*
78.	AY284695	Xyalidae	*Theristus*	*agilis*
79.	AF047889	Xyalidae	*Daptonema*	*procerus*
80.	AY854226	Xyalidae	*Daptonema*	*setosum*
81.	AY854225	Xyalidae	*Daptonema*	*oxycerca*
82.	AY854223	Xyalidae	*Daptonema*	*hirsutum*
83.	AY854224	Xyalidae	*Daptonema*	*normandicum*
84.	KC920423	Xyalidae	*Zygonemella*	*striata*
85.	AJ966491	Xyalidae	*Metadesmolaimus*	sp.
86.	AY854228	Sphaerolaimidae	*Sphaerolaimus*	*hirsutus*
87.	EF591333	Linhomoeidae	*Desmolaimus*	sp.
88.	EF591332	Linhomoeidae	*Desmolaimus*	sp.
89.	AY854229	Linhomoeidae	*Desmolaimus*	*zeelandicus*
90.	AY854230	Linhomoeidae	*Terschellingia*	*longicaudata*
91.	DQ408760	Siphonolaimidae	*Astomonema*	sp.
92.	DQ408761	Siphonolaimidae	*Astomonema*	sp.
93.	DQ408759	Siphonolaimidae	*Astomonema*	sp.
94.	JN815318	Tarvaiidae	*Tarvaia*	sp.
95.	JN815319	Ceramonematidae	*Ceramonema*	*inguinispina*
96.	JN815320	Ceramonematidae	*Ceramonema*	*altogolfi*
97.	JN815321	Ceramonematidae	*Ceramonema*	*reticulatum*
98.	FJ460256	Desmoscolecidae	*Tricoma*	sp.
99.	FJ460257	Desmoscolecidae	*Tricoma*	sp.
100.	FJ460255	Desmoscolecidae	*Paratricoma*	sp.
101.	FJ460252	Desmoscolecidae	*Desmoscolex*	sp.
102.	EF591342	Desmoscolecidae	*Desmoscolex*	sp.
103.	JN815322	Desmoscolecidae	*Desmoscolex*	sp.
104.	AY854203	Cyartonematidae	*Cyartonema*	*elegans*
105.	FJ182217	Draconematidae	*Draconema*	*japonicum*
106.	FJ182220	Draconematidae	*Paradraconema*	*jejuense*
107.	FJ182216	Draconematidae	*Dracograllus*	sp.
108.	FJ182223	Draconematidae	*Prochaetosoma*	sp.
109.	FJ182218	Epsilonematidae	*Epsilonema*	sp.
110.	AY854217	Desmodoridae	*Spirinia*	*parasitifera*
111.	EF591339	Desmodoridae	*Metachromadora*	sp.
112.	AY854216	Desmodoridae	*Metachromadora*	*remanei*
113.	FJ040469	Desmodoridae	*Metachromadora*	sp.
114.	Y16911	Desmodoridae	*Acanthopharynx*	*micans*
115.	AF047891	Desmodoridae	*Chromadoropsis*	*vivipara*

	Acc. number	Family	Genus	Species
116.	AY854215	Desmodoridae	*Desmodora*	*communis*
117.	Y16913	Desmodoridae	*Desmodora*	*ovigera*
118.	Y16923	Desmodoridae	*Xyzzors*	sp.
119.	KJ414468	Desmodoridae	*Leptonemella*	*vicina*
120.	KP943962	Desmodoridae	*Leptonemella*	cf. *juliae*
121.	KP943961	Desmodoridae	*Leptonemella*	*aphanothecae*
122.	Y16915	Desmodoridae	*Eubostrichus*	*dianae*
123.	Y16917	Desmodoridae	*Eubostrichus*	*topiarius*
124.	Y16916	Desmodoridae	*Eubostrichus*	*parasitiferus*
125.	KP943956	Desmodoridae	*Eubostrichus*	cf. *dianeae*
126.	Y16918	Desmodoridae	*Laxus*	*cosmopolitus*
127.	KT826596	Desmodoridae	*Laxus*	*oneistus*
128.	Y16919	Desmodoridae	*Laxus*	*oneistus*
129.	Y16922	Desmodoridae	*Stilbonema*	*majum*
130.	KJ414465	Desmodoridae	*Robbea*	*ruetzleri*
131.	KJ414466	Desmodoridae	*Robbea*	*hypermnestra*
132.	Y16921	Desmodoridae	*Robbea*	*hypermnestra*
133.	KP943964	Desmodoridae	*Robbea*	*hypermnestra*
134.	KP943955	Desmodoridae	*Catanema*	sp.
135.	Y16912	Desmodoridae	*Catanema*	sp.
136.	AY854218	Microlaimidae	*Calomicrolaimus*	*parahonestus*
137.	AY854219	Microlaimidae	*Calomicrolaimus*	sp.
138.	AY854220	Microlaimidae	*Molgolaimus*	*demani*
139.	FJ040477	Microlaimidae	*Prodesmodora*	sp.
140.	FJ040476	Microlaimidae	*Prodesmodora*	sp.
141.	JN815323	Haliplectidae	*Haliplectus*	sp.
142.	FJ969123	Haliplectidae	*Haliplectus*	cf. *dorsalis*
143.	AY284715	Selachinematidae	*Choanolaimus*	*psammophilus*
144.	FJ040467	Selachinematidae	*Choanolaimus*	*psammophilus*
145.	FJ040468	Selachinematidae	*Synonchiella*	sp.
146.	EF591338	Selachinematidae	*Halichoanolaimus*	sp.
147.	AY593942	Ethmolaimidae	*Ethmolaimus*	*pratensis*
148.	FJ040475	Ethmolaimidae	*Ethmolaimus*	*pratensis*
149.	AY593941	Achromadoridae	*Achromadora*	*ruricola*
150.	AY593940	Achromadoridae	*Achromadora*	cf. *terricola*
151.	AY854205	Chromadoridae	*Chromadora*	*nudicapitata*
152.	AY854206	Chromadoridae	*Chromadora*	sp.
153.	AY854209	Chromadoridae	*Dichromadora*	sp.
154.	FJ040506	Chromadoridae	*Dichromadora*	sp.
155.	FJ969119	Chromadoridae	*Chromadorita*	*leuckarti*
156.	FJ969138	Chromadoridae	*Punctodora*	*ratzeburgensis*

	Acc. number	Family	Genus	Species
157.	EF591341	Chromadoridae	*Prochromadora*	sp.
158.	FJ040473	Chromadoridae	*Chromadorita*	cf. *leuckarti*
159.	KJ636254	Chromadoridae	*Chromadorita*	*leuckarti*
160.	KJ636214	Chromadoridae	*Chromadorita*	*leuckarti*
161.	AY854208	Chromadoridae	*Chromadorita*	*tentabundum*
162.	AY854207	Chromadoridae	*Chromadorina*	*germanica*
163.	KJ636220	Chromadoridae	*Chromadorina*	*bioculata*
164.	KJ636221	Chromadoridae	*Chromadorina*	*bioculata*
165.	AY854204	Chromadoridae	*Atrochromadora*	*microlaima*
166.	AY854211	Chromadoridae	*Spilophorella*	*paradoxa*
167.	FJ040472	Chromadoridae	*Ptycholaimellus*	sp.
168.	AJ966495	Cyatholaimidae	*Paracyatholaimus*	*intermedius*
169.	FJ969133	Cyatholaimidae	*Paracyatholaimus*	*intermedius*
170.	AF036612	Cyatholaimidae	*Praeacanthonchus*	sp.
171.	AF047888	Cyatholaimidae	*Paracanthonchus*	*caecus*
172.	AY854214	Cyatholaimidae	*Praeacanthonchus*	*punctatus*
173.	AY854213	Cyatholaimidae	*Cyatholaimus*	sp.
174.	JQ071928	Monoposthiidae	*Nudora*	*ilhabelae*
175.	FJ040505	Monoposthiidae	*Monoposthia*	sp.
176.	AY854222	Monoposthiidae	*Nudora*	*bipapillata*
177.	AY284776	Dorylaimidae	*Dorylaimus*	*stagnalis*
178.	AY993978	Actinolaimidae	*Paractinolaimus*	*macrolaimus*
179.	AY284774	Nygolaimidae	*Paravulvus*	*hartingii*
180.	AY284770	Nygolaimidae	*Nygolaimus*	cf. *brachyuris*
181.	KJ636343	Isolaimiidae	*Isolaimium*	*multistriatum*
182.	KJ636356	Isolaimiidae	*Isolaimium*	*multistriatum*
183.	EF207244	Cryptonchidae	*Cryptonchus*	*tristis*
184.	FJ040479	Cryptonchidae	*Cryptonchus*	sp.
185.	FJ969116	Bathyodontidae	*Bathyodontus*	*mirus*
186.	AY552964	Bathyodontidae	*Bathyodontus*	*cylindricus*
187.	AY284765	Mononchidae	*Mononchus*	*aquaticus*
188.	AY297821	Mononchidae	*Mononchus*	*aquaticus*
189.	AJ966493	Mononchidae	*Mononchus*	*truncatus*
190.	FN400892	Mermithidae	*Isomermis*	*lairdi*
191.	AF036641	Mermithidae	*Mermis*	*nigrescens*
192.	AY284729	Prismatolaimidae	*Prismatolaimus*	*intermedius*
193.	AF036603	Prismatolaimidae	*Prismatolaimus*	*intermedius*
194.	AY593957	Prismatolaimidae	*Prismatolaimus*	*dolichurus*
195.	AY284725	Bastianiidae	*Bastiania*	*gracilis*
196.	FJ040487	Bastianiidae	*Dintheria*	*tenuissima*
197.	FJ969141	Onchulidae	*Stenonchulus*	*troglodytes*

	Acc. number	Family	Genus	Species
198.	AY284731	Tripylidae	*Tripyla*	cf. *filicaudata*
199.	AY284730	Tripylidae	*Tripyla*	cf. *filicaudata*
200.	KJ636224	Tripylidae	*Tripyla*	*glomerans*
201.	GQ503062	Tripylidae	*Tripyla*	*bioblitz*
202.	AY284737	Tripylidae	*Tripylella*	sp.
203.	FJ040488	Tripylidae	*Tripylella*	sp.
204.	AJ966506	Tobrilidae	*Tobrilus*	*gracilis*
205.	KJ636235	Tobrilidae	*Eutobrilus*	*nothus*
206.	KJ636226	Tobrilidae	*Eutobrilus*	*grandipapillatus*
207.	KJ636217	Tobrilidae	*Epitobrilus*	*stefanskii*
208.	KJ636231	Tobrilidae	*Semitobrilus*	*pellucidus*
209.	AF047890	Oncholaimidae	*Pontonema*	*vulgare*
210.	AF036642	Oncholaimidae	*Adoncholaimus*	sp.
211.	AY854195	Oncholaimidae	*Adoncholaimus*	*fuscus*
212.	AY854198	Oncholaimidae	*Viscosia*	*viscosa*
213.	AY854197	Oncholaimidae	*Viscosia*	sp.
214.	FJ040494	Oncholaimidae	*Viscosia*	sp.
215.	KR265042	Oncholaimidae	*Meyersia*	sp.
216.	FJ040502	Enchelidiidae	*Symplocostoma*	sp.
217.	AY854199	Enchelidiidae	*Calyptronema*	*maxweberi*
218.	FJ040503	Enchelidiidae	*Calyptronema*	sp.
219.	KR265038	Enchelidiidae	*Eurystomina*	sp.
220.	HM564491	Enchelidiidae	*Pareurystomina*	sp.
221.	HM564435	Enchelidiidae	*Pareurystomina*	sp.
222.	HM564537	Enchelidiidae	*Bathyeurystomina*	sp.
223.	HM564602	Enchelidiidae	*Bathyeurystomina*	sp.
224.	U88336	Enoplidae	*Enoplus*	*brevis*
225.	Y16914	Enoplidae	*Enoplus*	*meridionalis*
226.	AY854192	Enoplidae	*Enoplus*	*communis*
227.	AY854193	Thoracostomopsidae	*Enoploides*	*brunettii*
228.	FJ040490	Thoracostomopsidae	*Enoploides*	sp.
229.	KR265034	Thoracostomopsidae	*Enoplolaimus*	sp.
230.	HM564466	Thoracostomopsidae	*Enoplolaimus*	sp.
231.	HM564427	Thoracostomopsidae	*Enoplolaimus*	sp.
232.	HM564422	Thoracostomopsidae	*Enoplolaimus*	sp.
233.	KR265037	Thoracostomopsidae	*Epacanthion*	sp.
234.	KR265039	Thoracostomopsidae	*Mesacanthion*	sp.
235.	HM564625	Phanodermatidae	*Phanodermopsis*	sp.
236.	HM564510	Phanodermatidae	*Phanodermopsis*	sp.
237.	HM564575	Phanodermatidae	*Phanodermopsis*	sp.
238.	HM564523	Phanodermatidae	*Phanodermopsis*	sp.

	Acc. number	Family	Genus	Species
239.	HM564638	Anticomidae	*Anticoma*	sp.
240.	HM564627	Anticomidae	*Anticoma*	sp.
241.	HM564612	Anticomidae	*Cephalanticoma*	sp.
242.	FN433905	Leptosomatidae	*Thoracostoma*	*trachygaster*
243.	FN433903	Leptosomatidae	*Thoracostoma*	*microlobatum*
244.	FN433902	Leptosomatidae	*Pseudocella*	sp.
245.	FN433901	Leptosomatidae	*Pseudocella*	sp.
246.	FN433899	Leptosomatidae	*Deontostoma*	sp.
247.	HM564626	Leptosomatidae	*Leptosomatides*	sp.
248.	HM564630	Leptosomatidae	*Synonchus*	sp.
249.	HM564581	Trefusiidae	*Trefusia*	sp.
250.	AF329937	Trefusiidae	*Trefusia*	*zostericola*
251.	HM564585	Trefusiidae	*Trefusia*	sp.
252.	HM564478	Trefusiidae	*Trefusia*	sp.
253.	HM564606	Trefusiidae	*Rhabdocoma*	sp.
254.	HM564609	Trefusiidae	*Rhabdocoma*	sp.
255.	AJ966509	Trefusiidae	*Trischistoma*	*monohystera*
256.	AY284735	Trefusiidae	*Trischistoma*	sp.
257.	FJ969142	Trefusiidae	*Trischistoma*	sp.
258.	KJ636223	Trefusiidae	*Tripylina*	*arenicola*
259.	EF197728	Trefusiidae	*Tripylina*	sp.
260.	AJ966476	Tripyloididae	*Bathylaimus*	*assimilis*
261.	FJ040504	Tripyloididae	*Bathylaimus*	sp.
262.	AY854201	Tripyloididae	*Bathylaimus*	sp.
263.	HM564405	Tripyloididae	*Tripyloides*	sp.
264.	AY854202	Tripyloididae	*Tripyloides*	sp.
265.	FJ040491	Anoplostomatidae	*Anoplostoma*	sp.
266.	FJ040492	Anoplostomatidae	*Anoplostoma*	sp.
267.	AY590149	Anoplostomatidae	*Anoplostoma*	*rectospiculum*
268.	HM564528	Anoplostomatidae	*Chaetonema*	sp.
269.	HM564542	Anoplostomatidae	*Chaetonema*	sp.
270.	HM564533	Anoplostomatidae	*Chaetonema*	sp.
271.	KR265049	Rhabdodemaniidae	*Rhabdodemania*	sp.
272.	FJ040501	Oxystominidae	*Halalaimus*	sp.
273.	HM564589	Oxystominidae	*Halalaimus*	sp.
274.	HM564540	Oxystominidae	*Halalaimus*	sp.
275.	HM564652	Oxystominidae	*Halalaimus*	sp.
276.	HM564521	Oxystominidae	*Halalaimus*	sp.
277.	HM564420	Oxystominidae	*Halalaimus*	sp.
278.	HM564481	Oxystominidae	*Oxystomina*	sp.
279.	HM564548	Oxystominidae	*Oxystomina*	sp.

	Acc. number	Family	Genus	Species
280.	HM564403	Oxystominidae	*Oxystomina*	sp.
281.	HM564651	Oxystominidae	*Oxystomina*	sp.
282.	FJ040499	Oxystominidae	*Oxystomina*	sp.
283.	FJ040498	Oxystominidae	*Oxystomina*	sp.
284.	FJ040500	Oxystominidae	*Thalassoalaimus*	*pirum*
285.	HM564634	Oxystominidae	*Thalassoalaimus*	sp.
286.	HM564650	Oxystominidae	*Litinium*	sp.
287.	HM564649	Oxystominidae	*Litinium*	sp.
288.	HM564629	Oxystominidae	*Litinium*	sp.
289.	AY284738	Alaimidae	*Alaimus*	*parvus*
290.	AJ966514	Alaimidae	*Alaimus*	sp.
291.	FJ040489	Alaimidae	*Alaimus*	sp.
292.	AY284739	Alaimidae	*Paramphidelus*	*hortens*
293.	FJ040496	Ironidae	*Ironus*	sp.
294.	AJ966487	Ironidae	*Ironus*	*dentifurcatus*
295.	FJ040495	Ironidae	*Ironus*	*longicaudatus*
296.	KJ636218	Ironidae	*Ironus*	*macramphis*
297.	HM564604	Ironidae	*Dolicholaimus*	sp.
298.	JQ071931	Ironidae	*Trissonchulus*	sp.
299.	JQ071933	Ironidae	*Trissonchulus*	sp.
300.	AY854200	Ironidae	*Syringolaimus*	*striatocaudatus*
301.	FJ040497	Ironidae	*Syringolaimus*	sp.
302.	KJ636366	Rhabdolaimidae	*Rhabdolaimus*	*terrestris*
303.	FJ969139	Rhabdolaimidae	*Rhabdolaimus*	*aquaticus*
304.	AY552965	Campydoridae	*Campydora*	*demonstrans*
305.	FJ969118	Campydoridae	*Campydora*	*demonstrans*
306.	X87984	Priapulidae	*Priapulus*	*caudatus*
307.	X80234	Priapulidae	*Priapulus*	*caudatus*
308.	AF342790	Priapulidae	*Halicryptus*	*spinulosus*
