# Supplementary material for: Taxonomy assignment approach determines the efficiency of identification of OTUs in marine nematodes
Source: R Soc Open Sci. 2017 Aug 16;4(8):170315. doi: 10.1098/rsos.170315 (PMC5579096; doi:10.1098/rsos.170315)
Supplement: Supplementary Table 2 [file rsos170315supp5.pdf]

Supplementary file for the article:

Holovachov O, Haenel Q, Bourlat SJ, Jondelius U. Taxonomy assignment approach determines the efficiency of identification of OTUs in marine nematodes. *Royal Society Open Science.*

Supplementary Table 2. Taxonomic composition and relative abundance (% of the total number of specimens) of nematode species in Hållö site.

	Classification and identity	Flotation with $MgCl_2$	Flotation with H_2O
	**ORDER ENOPLIDA**		
	**Family Enoplidae**		
1	*Enoplus* sp.	0.46	0.43
	**Family Thoracostomopsidae**		
2	Enoplolaiminae gen. sp. 1	0.33	0.29
3	Enoplolaiminae gen. sp. 2	0.30	0.25
	**Family Phanodermatidae**		
4	Phanodermatidae gen. sp.	–	0.03
	**Family Anticomidae**		
5	*Anticoma* sp.	1.07	0.66
	**Family Oncholaimidae**		
6	*Viscosia* sp.	3.73	1.96
7	*Pontonema* sp.	0.15	0.03
8	Oncholaimidae gen. sp.	0.41	0.31
	**Family Enchelidiidae**		
9	*Symplocostoma* sp.	0.78	0.22
10	*Pareurystomina* sp.	0.08	0.03
	**Family Leptosomatidae**		
11	Leptosomatidae gen. sp.	–	0.01
	**Family Oxystominidae**		
12	*Nemanema* sp.	–	0.04
13	*Oxystomina* sp.	0.04	0.08
14	*Thalassoalaimus* sp.	0.08	0.16
15	*Halalaimus* sp. 1	0.23	0.66
16	*Halalaimus* sp. 2	–	0.01
17	*Maldivea* sp.	0.08	–
	**Family Tripyloididae**		
18	*Bathylaimus* sp.	–	0.01
	**Family Xennellidae**		
19	*Xennella* sp.	0.28	0.05
	**ORDER TRIPLONCHIDA**		
	**Family Rhabdodemaniidae**		
20	*Rhabdodemania* sp.	2.36	0.83

	Classification and identity	Flotation with $MgCl_2$	Flotation with H_2O
	**ORDER DESMOSCOLECIDA**		
	**Family Desmoscolecidae**		
21	*Desmoscolex* spp.	0.32	0.46
22	*Tricoma* (*Tricoma*) spp.	0.25	0.39
23	*Tricoma* (*Quadricoma*) spp.	3.05	3.49
	**Family Meyliidae**		
24	*Gerlachius* sp.	–	0.01
25	*Meylia* sp.	–	0.01
	**ORDER CHROMADORIDA**		
	**Family Chromadoridae**		
26	*Chromadora* sp.	0.04	0.07
27	*Chromadorita* sp.	1.53	1.39
28	*Chromadorella* cf. *filiformis*	0.68	0.46
29	*Neochromadora* sp. 1	2.33	1.81
30	*Neochromadora* sp. 2	0.24	0.04
31	*Neochromadora* sp. 3	0.16	0.10
32	*Dichromadora* sp. 1	0.33	0.87
33	*Dichromadora* sp. 2	0.04	–
34	*Euchromadora* sp.	1.03	0.36
35	*Innocuanema tentabundum*	1.19	1.30
36	*Ptycholaimellus* sp.	0.40	0.31
37	*Spilophorella* sp.	0.17	0.25
38	*Actinonema* sp.	0.45	0.14
39	Chromadoridae gen. spp.	0.28	0.16
	**Family Cyatholaimidae**		
40	*Pomponema* sp.	0.16	0.05
41	*Paracanthonchus* sp. 1	0.80	0.23
42	*Paracanthonchus* cf. *spectabilis*	0.04	0.16
43	*Paracanthonchus* cf. *longus*	1.89	2.26
44	*Preacanthonchus* cf. *inglisi*	0.80	0.55
45	*Paracyatholaimoides* sp.	0.23	1.98
46	*Marylinnia* sp	0.04	0.16
47	*Longicyatholaimus* sp.	–	0.14
48	Cyatholaimidae gen. sp.	0.17	0.04
	**Family Selachinematidae**		
49	*Halichoanolaimus* sp.	1.92	1.17
50	*Latronema* sp.	0.12	0.09
51	*Gammanema* sp.	0.21	0.13
52	*Choniolaimus* sp.	0.04	0.01
	**ORDER DESMODORIDA**		
	**Family Desmodoridae**		

	Classification and identity	Flotation with $MgCl_2$	Flotation with H_2O
53	*Desmodora* cf. *communis*	1.14	0.59
54	*Desmodora pontica*	15.53	14.26
55	*Desmodora granulata*	–	0.01
56	*Desmodorella schulzi*	10.49	10.22
57	*Bradylaimus pellita*	2.74	2.09
58	*Bolbonema brevicolle*	0.28	0.38
59	*Chromaspirina parapontica*	10.42	20.34
60	*Spirinia* sp. 1	0.35	0.27
61	*Spirinia* sp. 2	0.27	0.27
62	*Leptonemella* sp.	0.04	0.03
63	*Adelphos* sp.	0.04	0.01
	**Family Epsilonematidae**		
64	*Epsilonema*/*Metepsilonema* sp.	0.11	0.35
65	*Perepsilonema* sp.	1.08	4.97
	**Family Draconematidae**		
66	*Draconema cephalatum*	–	0.01
	**Family Microlaimidae**		
67	*Ixonema powelli*	0.21	0.95
68	*Microlaimus* sp. 1	8.24	5.81
69	*Microlaimus* sp. 2	1.97	2.03
70	*Microlaimus* sp. 3	1.42	2.00
71	*Microlaimus* sp. 4	0.04	–
72	*Microlaimus* sp. 5	–	0.04
73	*Microlaimus acanthus*	–	0.01
74	*Pseudoncholaimus dentatus*	0.13	0.08
	**Family Monoposthiidae**		
75	*Monoposthia costata*	0.31	0.23
	**ORDER MONHYSTERIDA**		
	**Family Xyalidae**		
76	*Daptonema* sp. 1	1.45	0.83
77	*Daptonema* sp. 2	–	0.03
78	*Theristus* sp. 1	1.39	1.43
79	*Theristus* sp. 2	0.04	0.03
80	*Gonionchus* sp.	0.11	0.07
81	*Echinotheristus* sp.	0.20	0.14
82	*Amphimonhystera* sp.	0.04	0.09
83	*Sphaerotheristus* sp.	–	0.01
84	Xyalidae gen. sp.	0.04	0.07
	**Family Monhysteridae**		
85	Monhysteridae gen. sp.	0.04	0.01
	**Family Siphonolaimidae**		

	Classification and identity	Flotation with $MgCl_2$	Flotation with H_2O
86	*Siphonolaimus* sp.	0.12	0.17
	**Family Linhomoeidae**		
87	*Disconema suecicum*	0.12	0.07
88	Linchomoeidae gen. spp.	0.37	0.20
	**ORDER ARAEOLAIMIDA**		
	**Family Comesomatidae**		
89	*Sabatieria* sp.	9.21	3.56
90	*Paramesonchium* sp. n.	0.12	–
	**Family Axonolaimidae**		
91	*Axonolaimus helgolandicus*	0.27	0.10
92	*Ascolaimus elongatus*	0.04	–
93	*Odontophora villoti*	1.35	2.85
	**Family Diplopeltidae**		
94	*Southerniella* sp.	0.08	0.07
95	*Diplopeltula* sp.	0.12	0.05
	**ORDER PLECTIDA**		
	**Family Leptolaimidae**		
96	*Leptolaimus pellucidus*	0.08	0.05
97	*Leptolaimus* sp.	0.04	0.03
98	*Leptolaimus* sp. n.	0.40	0.23
99	*Manunema* sp.	0.04	0.01
	**Family Camacolaimidae**		
100	*Stephanolaimus elegans*	0.12	0.03
101	*Dagda bipapillata*	–	0.01
102	*Deontolaimus* sp.	0.04	0.04
103	*Onchium* sp.	–	0.01
	**Family Diplopeltoididae**		
104	*Diplopeltoides* sp. n. 1	0.04	–
105	*Diplopeltoides* sp. n. 2	–	0.01
	**Family Tarvaiidae**		
106	*Tarvaia* sp.	–	0.03
	**Family Tubolaimoididae**		
107	*Tubolaimoides* sp.	–	0.01
108	Nematoda indet.	0.04	0.14
