# Supplementary material for: Taxonomy assignment approach determines the efficiency of identification of OTUs in marine nematodes
Source: R Soc Open Sci. 2017 Aug 16;4(8):170315. doi: 10.1098/rsos.170315 (PMC5579096; doi:10.1098/rsos.170315)
Supplement: Supplementary Table 3 [file rsos170315supp6.pdf]

Supplementary file for the article:

Holovachov O, Haenel Q, Bourlat SJ, Jondelius U. Taxonomy assignment approach determines the efficiency of identification of OTUs in marine nematodes. *Royal Society Open Science.*

Supplementary Table 3. Taxonomic composition and relative abundance (% of the total number of specimens) of nematode species in Telekabeln site.

	Classification and identity	Siphoning	Flotation with H_2O
	**ORDER ENOPLIDA**		
	**Family Thoracostomopsidae**		
1	Enoplolaiminae gen. sp.	3.45	0.08
	**Family Anticomidae**		
2	*Anticoma* sp.	0.29	0.04
	**Family Oncholaimidae**		
3	*Viscosia* sp. 1	3.16	0.97
4	*Viscosia* sp. 2	6.66	0.93
5	*Viscosia* sp. 3	0.19	0.04
6	Oncholaimidae gen. sp.	0.05	–
	**Family Enchelidiidae**		
7	*Symplocostoma* sp.	0.10	0.04
8	*Polygastrophora* sp.	1.05	0.04
	**Family Ironidae**		
9	*Thalassironus* sp.	0.34	0.43
	**Family Oxystominidae**		
10	*Oxystomina* sp. 1	0.24	0.23
11	*Oxystomina* sp. 2	1.34	0.12
12	*Oxystomina* sp. 3	0.05	0.04
13	*Lithinium* sp.	0.05	–
14	*Thalassoalaimus* sp. 1	0.19	0.66
15	*Thalassoalaimus* sp. 2	0.05	0.16
16	*Thalassoalaimus* sp. 3	–	0.04
17	*Halalaimus* sp. 1	2.59	1.48
18	*Halalaimus* sp. 2	1.15	0.74
19	*Halalaimus* sp. 3	0.43	0.97
20	*Halalaimus* sp. 4	1.49	1.13
21	*Halalaimus* sp. 5	0.10	0.16
22	*Halalaimus* sp. 6	0.10	–
23	*Halalaimus* sp. 7	–	0.08
24	*Halalaimus* sp. 8	0.86	1.13
25	*Wieseria* sp.	0.29	2.95
26	Oxystominidae gen. sp.	0.81	0.93

	Classification and identity	Siphoning	Flotation with H_2O
	**Family Tripyloididae**		
27	*Tripyloides* sp.	0.43	0.04
28	*Bathylaimus* sp.	1.49	0.23
	**Family Trefusiidae**		
29	*Trefusia* sp.	–	1.44
	**ORDER TRIPLONCHIDA**		
	**Family Pandolaimidae**		
30	*Pandolaimus* sp.	0.48	1.63
	**Family Rhabdodemaniidae**		
31	*Rhabdodemania* sp.	0.72	0.19
	**ORDER DESMOSCOLECIDA**		
	**Family Desmoscolecidae**		
32	*Desmoscolex* sp.	0.67	1.71
33	*Tricoma (Quadricoma)* sp. 1	2.83	16.11
34	*Tricoma (Quadricoma)* sp. 2	5.56	1.28
35	*Tricoma (Quadricoma)* sp. 3	0.19	0.39
	**Family Cyartonematidae**		
36	*Cyartonema* sp.	–	1.05
	**ORDER CHROMADORIDA**		
	**Family Chromadoridae**		
37	*Chromadorita* sp. 1	0.58	0.47
38	*Chromadorita* sp. 2	0.14	–
39	*Neochromadora* sp. 1	0.05	–
40	*Neochromadora* sp. 2	0.38	0.04
41	*Actinonema* sp.	2.25	0.74
42	*Trochamus* sp.	1.49	0.78
43	*Chromadoridae* gen. spp.	0.14	0.74
44	*Acantholaimus* sp.	–	0.08
	**Family Cyatholaimidae**		
45	*Paracanthonchus* cf. *longicaudatus*	0.05	2.06
46	*Marylinnia* sp	6.28	6.29
47	*Paralongicyatholaimus* sp.	0.10	1.01
48	*Craspodema* sp.	0.19	–
	**Family Selachinematidae**		
49	*Halichoanolaimus* sp.	0.10	–
50	*Cheironchus* sp.	–	0.12
51	*Synonchiella* sp.	–	0.19
52	*Choniolaimus* sp. 1	0.24	1.55
53	*Choniolaimus* sp. 2	–	0.04
	**ORDER DESMODORIDA**		
	**Family Desmodoridae**		

	Classification and identity	Siphoning	Flotation with H_2O
54	*Desmodora* cf. *communis*	0.05	–
55	*Desmodora pontica*	0.62	0.19
56	*Desmodorella tenuispiculum*	3.60	3.18
57	*Chromaspirina* sp.	–	0.12
58	*Spirinia* sp.	0.10	0.39
	**Family Microlaimidae**		
59	*Microlaimus* sp. 1	0.24	0.85
60	*Microlaimus* sp. 2	–	2.02
61	*Microlaimus* sp. 3	–	0.74
62	*Bolbolaimus* sp	–	0.04
	**Family Monoposthiidae**		
63	Monoposthiidae gen. sp.	0.05	–
	**Family Richtersiidae**		
64	*Richtersia* sp.	2.01	5.90
	**ORDER MONHYSTERIDA**		
	**Family Xyalidae**		
65	*Daptonema* sp.	1.58	0.08
66	*Metadesmolaimus* sp.	3.07	4.62
67	*Gnomoxyala* sp.	–	0.19
68	Xyalidae gen. sp.	0.14	0.97
	**Family Sphaerolaimidae**		
69	*Sphaerolaimus* sp. 1	1.77	1.82
70	*Sphaerolaimus* sp. 2	0.58	0.12
71	*Parasphaerolaimus* sp.	0.38	0.47
	**Family Siphonolaimidae**		
72	*Siphonolaimus* sp.	–	0.08
	**Family Linhomoeidae**		
73	*Disconema* sp.	0.10	–
74	*Eleutherolaimus* sp.	0.58	1.32
75	*Linchomoeidae* gen. sp.	2.11	0.16
76	*Monhysteroides* sp.	–	0.97
77	*Terschellingia* sp. 1	–	1.71
78	*Terschellingia* sp. 2	0.05	0.08
	**ORDER ARAEOLAIMIDA**		
	**Family Comesomatidae**		
79	*Sabatieria* spp.	18.31	8.46
80	*Setosabatieria* sp.	5.61	2.80
81	*Dorylaimopsis* sp.	0.86	3.69
82	*Laimella* sp.	–	0.04
	**Family Axonolaimidae**		
83	*Axonolaimus* sp.	5.42	0.85

	Classification and identity	Siphoning	Flotation with H_2O
84	*Odontophora* sp.	0.05	0.08
	**Family Diplopeltidae**		
85	*Araeolaimus spinosus*	0.96	0.08
86	*Campylaimus rimatus*	0.14	0.04
87	*Campylaimus amphidialis*	–	0.31
88	*Campylaimus tkatchevi*	0.05	0.39
89	*Campylaimus orientalis*	–	0.31
90	*Campylaimus* sp.	0.05	0.19
91	*Pararaeolaimus* sp.	0.19	0.04
92	*Diplopeltula* sp.	0.10	0.04
	**ORDER PLECTIDA**		
	**Family Leptolaimidae**		
93	*Leptolaimus danicus*	0.05	0.43
94	*Leptolaimus septempapillatus*	–	0.19
95	*Leptolaimus venustus*	–	0.04
96	*Leptolaimus* sp.	–	0.08
97	*Antomicron lorenzeni*	–	0.08
98	*Antomicron quindecimpapillatus*	–	0.04
99	*Leptolaimoides* sp.	0.05	–
	**Family Camacolaimidae**		
100	*Alaimella* sp.	0.34	0.19
101	Camacolaimidae gen. sp.	0.05	0.12
102	*Deontolaimus catalinae*	–	0.04
103	*Deontolaimus* sp.	–	0.08
	**Family Rhadinematidae**		
104	*Rhadinema timmi*	0.05	0.04
	**Family Ceramonematidae**		
105	*Pselionema* sp.	0.62	0.82
106	*Dasynemoides* sp.	–	0.04
	**Family Diplopeltoididae**		
107	*Diplopeltoides ornatus*	0.05	0.04
108	*Diplopeltoides linkei*	–	0.04
109	*Diplopeltoides nudus*	–	0.08
110	*Diplopeltoides asetosus*	–	0.04
111	*Diplopeltoides* sp. n.	–	1.67
	**Family Aegialoalaimidae**		
112	*Aegialoalaimus* sp.	0.29	0.16
	**Family Paramicrolaimidae**		
113	*Paramicrolaimus* sp.	–	0.04
114	Nematoda indet.	0.38	0.89
