# Supplementary material for: Taxonomy assignment approach determines the efficiency of identification of OTUs in marine nematodes
Source: R Soc Open Sci. 2017 Aug 16;4(8):170315. doi: 10.1098/rsos.170315 (PMC5579096; doi:10.1098/rsos.170315)
Supplement: Supplementary Table 4 [file rsos170315supp7.pdf]

Supplementary file for the article:

Holovachov O, Haenel Q, Bourlat SJ, Jondelius U. Taxonomy assignment approach determines the efficiency of identification of OTUs in marine nematodes. *Royal Society Open Science.*

Supplementary Table 4. Results of alignment-based taxonomy assignment using BLASTN 2.5.0+ against the nucleotide collection of the NCBI database.

OTU ID	Best hit	identity %	cover %	Family identification
HE1.SSU848264	*Paracyatholaimus intermedius*	92	100	Cyatholaimidae
HE1.SSU850987	*Eubostrichus topiarius*	89	100	unassigned
HE1.SSU856624	*Thalassoalaimus* sp.	94	88	unassigned
HE1.SSU856738	*Calomicrolaimus parahonestus*	92	100	Microlaimidae
HE1.SSU858060	*Dichromadora* sp.	94	98	unassigned
HE1.SSU867071	*Synonchiella* sp.	95	93	unassigned
HE2.SSU637072	*Campydora* sp.	90	99	unassigned
HE2.SSU637135	*Neochromadora* sp.	90	99	unassigned
HE2.SSU644966	*Paracanthonchus* sp.	91	100	Cyatholaimidae
HE2.SSU654005	*Rhabdodemania* sp.	93	84	unassigned
HE2.SSU655107	*Theristus* sp.	88	99	unassigned
HE2.SSU659506	*Neochromadora* sp.	97	100	Chromadoridae
HE3.SSU110275	*Enoplus communis* sp.	98	100	Enoplidae
HE3.SSU117415	*Paracyatholaimus* sp.	91	100	Cyatholaimidae
HE3.SSU118424	*Oxystomina* sp.	89	98	unassigned
HE3.SSU124287	*Enoploides brunettii*	98	100	Thoracostomopsidae
HE3.SSU124998	*Astomonema* sp.	85	100	unassigned
HE4.SSU913283	*Anaplectus* sp.	89	100	unassigned
HE5.SSU181724	*Neochromadora* sp.	87	100	unassigned
HE5.SSU188855	*Symplocostoma* sp.	99	98	unassigned
HE6.SSU355777	*Achromadora terricola*	93	100	Achromadoridae
HE6.SSU358048	*Neochromadora* sp.	92	100	Chromadoridae
HE6.SSU360897	*Desmodora ovigera*	93	92	unassigned
HE6.SSU361449	*Syringolaimus* sp.	94	100	Ironidae
HE6.SSU365256	*Desmodora ovigera*	97	76	unassigned
HE6.SSU368318	*Prochaetosoma* sp.	93	92	unassigned
HE6.SSU370544	*Daptonema normandicum*	97	99	unassigned
HE6.SSU378839	*Calomicrolaimus parahonestus*	82	99	unassigned
HE6.SSU383414	*Sabatieria* sp.	94	100	Comesomatidae
HE6.SSU383888	*Neochromadora* sp.	90	100	Chromadoridae
HE7.SSU232624	*Leptolaimus* sp.	91	98	unassigned
HE7.SSU256492	*Neochromadora* sp.	95	100	Chromadoridae
HE8.SSU829972	*Anticoma* sp.	98	88	unassigned

OTU ID	Best hit	identity %	cover %	Family identification
HE8.SSU843570	*Neochromadora* sp.	91	100	Chromadoridae
HE9.SSU305678	*Theristus* sp.	88	100	unassigned
HF1.SSU759758	*Camacolaimus* sp.	98	98	unassigned
HF1.SSU763392	*Praeacanthonchus punctatus*	94	100	Cyatholaimidae
HF1.SSU764346	*Paracanthonchus* sp.	93	100	Cyatholaimidae
HF1.SSU774294	*Mermis nigrescens*	95	100	Mermithidae
HF1.SSU779114	*Odontophora rectangula*	99	98	unassigned
HF1.SSU780927	*Desmolaimus* sp.	95	76	unassigned
HF2.SSU192072	*Chromadorina* sp.	100	98	unassigned
HF2.SSU204352	*Anaplectus* sp.	87	100	unassigned
HF2.SSU205129	*Neochromadora*	89	100	unassigned
HF2.SSU208147	*Synonchiella* or *Halichoanolaimus*	98	98	unassigned
HF2.SSU210357	*Calyptronema* sp.	92	100	Enchelidiidae
HF3.SSU989895	*Setostephanolaimus* sp.	92	98	unassigned
HF3.SSU990962	*Neochromadora* sp.	90	100	Chromadoridae
HF4.SSU606153	*Neochromadora* sp.	99	100	Chromadoridae
HF4.SSU614317	*Sabatieria* sp.	92	100	Comesomatidae
HF4.SSU619471	*Molgolaimus demani*	90	100	Microlaimidae
HF4.SSU620879	*Punctodora ratzeburgensis*	92	100	Chromadoridae
HF4.SSU622464	*Plectus aquatilis*	95	100	Plectidae
HF4.SSU624085	*Prochaetosoma* sp.	90	93	unassigned
HF4.SSU625424	*Sabatieria* sp.	87	100	unassigned
HF4.SSU628562	*Stilbonema majum*	82	100	unassigned
HF4.SSU631524	*Leptolaimus* sp.	97	92	unassigned
HF4.SSU632264	*Diplopeltula* sp.	95	98	unassigned
HF4.SSU635045	*Chromadorina* sp.	96	98	unassigned
HF5.SSU991188	*Oncholaimidae indet.*	93	88	unassigned
HF5.SSU995414	*Rhabdolaimus aquaticus*	90	100	Rhabdolaimidae
HF6.SSU329881	*Desmodora ovigera*	99	93	unassigned
HF6.SSU338435	*Neochromadora* sp.	89	91	unassigned
HF6.SSU338739	*Astomonema* sp.	89	100	unassigned
HF7.SSU385021	*Neochromadora* sp.	90	100	Chromadoridae
HF7.SSU390110	*Desmolaimus* sp.	97	98	unassigned
HF7.SSU398053	*Camacolaimus* sp.	94	77	unassigned
HF7.SSU407024	*Achromadora ruricola*	95	95	unassigned
HF7.SSU407761	*Desmolaimus* sp.	94	98	unassigned
HF7.SSU409331	*Pomponema* sp.	94	76	unassigned
HF8.SSU795426	*Cyatholaimus* sp.	90	100	Cyatholaimidae
HF9.SSU14048	*Calomicrolaimus* sp.	93	76	unassigned
HF9.SSU14296	*Pomponema* sp.	85	90	unassigned
HF9.SSU17250	*Enoploides brunettii*	91	100	Thoracostomopsidae

OTU ID	Best hit	identity %	cover %	Family identification
HF9.SSU17844	*Pomponema* sp.	94	89	unassigned
HF9.SSU18227	*Neochromadora* sp.	91	100	Chromadoridae
HF9.SSU19963	*Desmoscolex* sp.	87	100	unassigned
HF9.SSU20251	*Calomicrolaimus parahonestus*	98	100	Microlaimidae
HF9.SSU22538	*Mermis nigrescens*	90	100	Mermithidae
TF1.SSU676746	*Haliplectus* or *Prodesmodora*	90	100	unassigned
TF1.SSU677162	*Anaplectus* sp.	89	100	unassigned
TF1.SSU681557	*Pseudocella* sp.	87	100	unassigned
TF1.SSU688192	*Terschellingia longicaudata*	90	100	Linhomoeidae
TF1.SSU692690	*Synonchiella* or *Halichoanolaimus*	91	100	Selachinematidae
TF1.SSU694267	*Desmoscolex* sp.	89	98	unassigned
TF1.SSU694751	*Neochromadora* sp.	90	100	Chromadoridae
TF1.SSU698227	*Teratocephalus lirellus*	91	100	Teratocephalidae
TF1.SSU700188	*Terschellingia longicaudata*	96	100	Linhomoeidae
TF1.SSU703579	*Astomonema* sp.	90	100	Siphonolaimidae
TF1.SSU710679	*Cyatholaimus* sp.	99	100	Cyatholaimidae
TF1.SSU734804	*Astomonema* sp.	90	100	Siphonolaimidae
TF3.SSU956521	*Sabatieria* sp.	90	100	Comesomatidae
TF3.SSU960449	*Desmoscolex* sp.	88	98	unassigned
TF3.SSU966338	*Theristus* sp.	91	99	unassigned
TF4.SSU144249	*Paracanthonchus* sp.	97	99	unassigned
TF4.SSU150234	*Metachromadora* sp.	91	100	Desmodoridae
TF5.SSU410031	*Praeacanthonchus* sp.	88	100	unassigned
TF5.SSU419519	*Desmoscolex* sp.	92	98	unassigned
TF5.SSU430294	*Theristus* sp.	97	99	unassigned
TF5.SSU437076	*Setosabatieria hilarula*	92	100	Comesomatidae
TF5.SSU444034	*Tripylella* sp.	93	98	unassigned
TF5.SSU446087	*Tarvaia* sp.	91	97	unassigned
TF5.SSU453472	*Tripylina* sp.	89	100	unassigned
TF5.SSU457543	*Oxystomina* sp.	92	98	unassigned
TF5.SSU459305	*Viscosia viscosa*	92	100	Oncholaimidae
TF5.SSU466315	*Daptonema* sp.	96	99	unassigned
TF6.SSU33463	*Oxystomina* sp.	96	98	unassigned
TF6.SSU33935	*Desmolaimus* sp.	93	98	unassigned
TF6.SSU36442	*Desmoscolex* sp.	89	98	unassigned
TF6.SSU37421	*Prochaetosoma* sp.	93	92	unassigned
TF6.SSU41803	*Daptonema* sp.	88	98	unassigned
TF6.SSU47996	*Viscosia* sp.	92	100	Oncholaimidae
TF6.SSU48167	*Sabatieria* sp.	99	100	Comesomatidae
TF6.SSU53456	*Viscosia* sp.	99	98	unassigned
TF6.SSU54250	*Calomicrolaimus parahonestus*	93	100	Microlaimidae

OTU ID	Best hit	identity %	cover %	Family identification
TF6.SSU58877	*Tarvaia* or *Desmoscolex*	91	97	unassigned
TF6.SSU74955	*Paracanthonchus* sp.	93	100	Cyatholaimidae
TF6.SSU82210	*Stilbonema* sp.	85	100	unassigned
TF6.SSU84268	*Spectatus spectatus*	82	90	unassigned
TF6.SSU98667	*Campydora* sp.	86	92	unassigned
TS1.SSU270885	*Tarvaia* sp.	88	97	unassigned
TS1.SSU284163	*Desmoscolex* sp.	87	98	unassigned
TS2.SSU821962	*Bathylaimus* sp.	91	100	Tripyloididae
TS2.SSU823349	*Bathylaimus* sp.	90	88	unassigned
TS3.SSU475561	*Astomonema* sp.	91	100	Siphonolaimidae
TS3.SSU489684	*Desmoscolex* sp.	95	97	unassigned
TS3.SSU503133	*Tripyloides* sp.	94	100	Tripyloididae
TS3.SSU508400	*Desmolaimus* sp.	90	87	unassigned
TS4.SSU543236	*Thalassoalaimus* sp.	90	88	unassigned
TS4.SSU544032	*Desmoscolex* sp.	92	98	unassigned
TS5.SSU874117	*Halalaimus* sp.	91	98	unassigned
TS5.SSU875407	*Sabatieria celtica*	93	100	Comesomatidae
TS5.SSU881546	*Theristus* sp.	90	99	unassigned
TS5.SSU900338	*Leptolaimus* sp.	94	98	unassigned
TS5.SSU901243	*Dolicholaimus* sp.	96	88	unassigned
TS6.SSU559765	*Tripyloides* sp.	95	89	unassigned
TS6.SSU570763	*Axonolaimus* sp.	88	98	unassigned
TS6.SSU587229	*Viscosia viscosa*	99	100	Oncholaimidae
HE6.SSU372021	*Eumonhystera filiformis*	91	99	unassigned
