# Supplementary material for: Taxonomy assignment approach determines the efficiency of identification of OTUs in marine nematodes
Source: R Soc Open Sci. 2017 Aug 16;4(8):170315. doi: 10.1098/rsos.170315 (PMC5579096; doi:10.1098/rsos.170315)
Supplement: Supplementary Table 5 [file rsos170315supp8.pdf]

Supplementary file for the article:

Holovachov O, Haenel Q, Bourlat SJ, Jondelius U. Taxonomy assignment approach determines the efficiency of identification of OTUs in marine nematodes. *Royal Society Open Science.*

Supplementary Table 5. Results of alignment-based taxonomy assignment using LCAClassifier of CREST against built-in reference database.

OTU ID	Best hit	Family identification
HE1.SSU848264	Chromadorea; Chromadorida	unassigned
HE1.SSU850987	Chromadorea	unassigned
HE1.SSU856624	Enoplea; Enoplida; Oxystominoidea	unassigned
HE1.SSU856738	Chromadorea; Desmodorida; Richtersioidea; Microlaimidae	Microlaimidae
HE1.SSU858060	Chromadorea; Chromadorida	unassigned
HE1.SSU867071	Chromadorea	unassigned
HE2.SSU637072	Enoplea; Enoplida; Enoploidea	unassigned
HE2.SSU637135	Chromadorea; Chromadorida; Chromadoridae	Chromadoridae
HE2.SSU644966	Chromadorea; Chromadorida	unassigned
HE2.SSU654005	Enoplea	unassigned
HE2.SSU655107	Chromadorea; Unknown Chromadorea	unassigned
HE2.SSU659506	Chromadorea; Chromadorida	unassigned
HE3.SSU110275	Enoplea; Enoplida; Enoploidea; Enoplidae; Enoplus	Enoplidae
HE3.SSU117415	Chromadorea; Chromadorida	unassigned
HE3.SSU118424	Arthropoda; Crustacea; Maxillopoda; Copepoda	unassigned
HE3.SSU124287	Enoplea; Enoplida; Enoploidea; Thoracostomopsidae; Enoploides	Thoracostomopsidae
HE3.SSU124998	Chromadorea	unassigned
HE4.SSU913283	Chromadorea	unassigned
HE5.SSU181724	Chromadorea; Chromadorida	unassigned
HE5.SSU188855	Enoplea; Enoplida; Oncholaimoidea	unassigned
HE6.SSU355777	Chromadorea; Chromadorida	unassigned
HE6.SSU358048	Chromadorea; Chromadorida	unassigned
HE6.SSU360897	Chromadorea; Desmodorida	unassigned
HE6.SSU361449	Enoplea; Enoplida; Ironoidea	unassigned
HE6.SSU365256	Chromadorea; Desmodorida	unassigned
HE6.SSU368318	Chromadorea; Desmodorida; Desmodoridae	Desmodoridae
HE6.SSU370544	Chromadorea; Monhysterida; Xyalidae; Daptonema	Xyalidae
HE6.SSU378839	Chromadorea	unassigned
HE6.SSU383414	Chromadorea; Monhysterida	unassigned
HE6.SSU383888	Chromadorea	unassigned
HE7.SSU232624	Chromadorea; Araeolaimida; Leptolaimoidea	unassigned
HE7.SSU256492	Chromadorea; Chromadorida	unassigned
HE8.SSU829972	Enoplea	unassigned

OTU ID	Best hit	Family identification
HE8.SSU843570	Chromadorea; Chromadorida	unassigned
HE9.SSU305678	Chromadorea	unassigned
HF1.SSU759758	Chromadorea; Araeolaimida; Leptolaimoidea; Leptolaimidae	Leptolaimidae
HF1.SSU763392	Chromadorea; Chromadorida	unassigned
HF1.SSU764346	Chromadorea; Chromadorida	unassigned
HF1.SSU774294	Enoplea; Mononchida; Mononchina; Anatonchoidea	unassigned
HF1.SSU779114	Chromadorea; Araeolaimida; Axonolaimoidea; Axonolaimidae; *Odontophora rectangula*	Axonolaimidae
HF1.SSU780927	Chromadorea; Araeolaimida	unassigned
HF2.SSU192072	Chromadorea; Chromadorida; Chromadoridae; *Chromadora nudicapitata*	Chromadoridae
HF2.SSU204352	Chromadorea; Araeolaimida; Leptolaimoidea	unassigned
HF2.SSU205129	Chromadorea; Chromadorida	unassigned
HF2.SSU208147	Nematoda	unassigned
HF2.SSU210357	Enoplea; Enoplida; Oncholaimoidea	unassigned
HF3.SSU989895	Chromadorea; Araeolaimida; Plectoidea	unassigned
HF3.SSU990962	Chromadorea	unassigned
HF4.SSU606153	Chromadorea; Chromadorida; Chromadoridae	Chromadoridae
HF4.SSU614317	Chromadorea; Monhysterida; Comesomatidae; *Sabatieria* sp.	Comesomatidae
HF4.SSU619471	Chromadorea; Desmodorida; Richtersioidea	unassigned
HF4.SSU620879	Chromadorea; Chromadorida	unassigned
HF4.SSU622464	Nematoda	unassigned
HF4.SSU624085	Chromadorea; Desmodorida	unassigned
HF4.SSU625424	Chromadorea	unassigned
HF4.SSU628562	Nematoda	unassigned
HF4.SSU631524	Chromadorea; Araeolaimida; Leptolaimoidea; Leptolaimidae; *Leptolaimus* sp.	Leptolaimidae
HF4.SSU632264	Chromadorea	unassigned
HF4.SSU635045	Chromadorea; Chromadorida	unassigned
HF5.SSU991188	Enoplea; Enoplida; Oncholaimoidea	unassigned
HF5.SSU995414	Enoplea; Enoplida; Ironoidea; Ironidae	Ironidae
HF6.SSU329881	Chromadorea; Desmodorida; Desmodoridae; Desmodorinae	Desmodoridae
HF6.SSU338435	Chromadorea	unassigned
HF6.SSU338739	Chromadorea	unassigned
HF7.SSU385021	Chromadorea	unassigned
HF7.SSU390110	Chromadorea; Araeolaimida	unassigned
HF7.SSU398053	Chromadorea; Araeolaimida; Leptolaimoidea	unassigned
HF7.SSU407024	Chromadorea; Chromadorida	unassigned
HF7.SSU407761	Chromadorea; Araeolaimida	unassigned
HF7.SSU409331	Enoplea	unassigned
HF8.SSU795426	Chromadorea	unassigned
HF9.SSU14048	Chromadorea; Desmodorida; Richtersioidea	unassigned
HF9.SSU14296	Nematoda	unassigned

OTU ID	Best hit	Family identification
HF9.SSU17250	Enoplea; Enoplida; Enoploidea; Thoracostomopsidae	Thoracostomopsidae
HF9.SSU17844	Chromadorea	unassigned
HF9.SSU18227	Chromadorea; Chromadorida	unassigned
HF9.SSU19963	Chromadorea	unassigned
HF9.SSU20251	Chromadorea; Desmodorida; Richtersioidea; Microlaimidae; *Calomicrolaimus* sp.	Microlaimidae
HF9.SSU22538	Enoplea; Mononchida; Mononchina; Anatonchoidea	unassigned
TF1.SSU676746	Chromadorea	unassigned
TF1.SSU677162	Chromadorea; Araeolaimida	unassigned
TF1.SSU681557	Enoplea	unassigned
TF1.SSU688192	Chromadorea; Monhysterida	unassigned
TF1.SSU692690	Chromadorea	unassigned
TF1.SSU694267	Chromadorea	unassigned
TF1.SSU694751	Chromadorea	unassigned
TF1.SSU698227	Chromadorea; Rhabditida; Teratocephaloidea	unassigned
TF1.SSU700188	Chromadorea; Monhysterida; Monhysterida incertae sedis	unassigned
TF1.SSU703579	Chromadorea	unassigned
TF1.SSU710679	Chromadorea; Chromadorida; Cyatholaimidae; *Paracanthonchus* sp.	Cyatholaimidae
TF1.SSU734804	Chromadorea	unassigned
TF3.SSU956521	Chromadorea; Monhysterida	unassigned
TF3.SSU960449	Chromadorea	unassigned
TF3.SSU966338	Chromadorea	unassigned
TF4.SSU144249	Chromadorea; Chromadorida	unassigned
TF4.SSU150234	Chromadorea; Desmodorida	unassigned
TF5.SSU410031	Nematoda	unassigned
TF5.SSU419519	Chromadorea	unassigned
TF5.SSU430294	Chromadorea; Monhysterida	unassigned
TF5.SSU437076	Chromadorea; Monhysterida; Comesomatidae; *Setosabatieria hilarula*	Comesomatidae
TF5.SSU444034	Enoplea; Enoplida; Tripyloidea	unassigned
TF5.SSU446087	Nematoda	unassigned
TF5.SSU453472	Enoplea	unassigned
TF5.SSU457543	Chromadorea	unassigned
TF5.SSU459305	Enoplea; Enoplida; Oncholaimoidea; Oncholaimidae; *Oncholaimus* sp.	Oncholaimidae
TF5.SSU466315	Chromadorea; Monhysterida	unassigned
TF6.SSU33463	Enoplea	unassigned
TF6.SSU33935	Chromadorea; Araeolaimida	unassigned
TF6.SSU36442	Chromadorea	unassigned
TF6.SSU37421	Chromadorea; Desmodorida; Desmodoridae; Spiriniinae	Desmodoridae
TF6.SSU41803	Chromadorea; Monhysterida; Xyalidae	Xyalidae
TF6.SSU47996	Enoplea; Enoplida; Oncholaimoidea; Enchelidiidae; *Pareurystomina* sp.	Enchelidiidae
TF6.SSU48167	Chromadorea; Monhysterida; Comesomatidae; *Sabatieria* sp.	Comesomatidae

OTU ID	Best hit	Family identification
TF6.SSU53456	Enoplea; Enoplida; Oncholaimoidea; Oncholaimidae; *Viscosia* sp.	Oncholaimidae
TF6.SSU54250	Chromadorea; Desmodorida; Richtersioidea	unassigned
TF6.SSU58877	Chromadorea	unassigned
TF6.SSU74955	Chromadorea	unassigned
TF6.SSU82210	Chromadorea	unassigned
TF6.SSU84268	Chromadorea	unassigned
TF6.SSU98667	Nematoda	unassigned
TS1.SSU270885	Chromadorea	unassigned
TS1.SSU284163	Annelida; Polychaeta; Scolecida	unassigned
TS2.SSU821962	Enoplea; Enoplida; Tripyloidoidea	unassigned
TS2.SSU823349	Enoplea; Enoplida; Tripyloidoidea	unassigned
TS3.SSU475561	Chromadorea	unassigned
TS3.SSU489684	Chromadorea	unassigned
TS3.SSU503133	Enoplea; Enoplida; Tripyloidoidea	unassigned
TS3.SSU508400	Chromadorea	unassigned
TS4.SSU543236	Enoplea	unassigned
TS4.SSU544032	Chromadorea	unassigned
TS5.SSU874117	Enoplea	unassigned
TS5.SSU875407	Chromadorea; Monhysterida; Comesomatidae; *Setosabatieria hilarula*	Comesomatidae
TS5.SSU881546	Chromadorea	unassigned
TS5.SSU900338	Chromadorea; Araeolaimida; Leptolaimoidea	unassigned
TS5.SSU901243	Enoplea	unassigned
TS6.SSU559765	Enoplea; Enoplida; Tripyloidoidea	unassigned
TS6.SSU570763	Chromadorea; Araeolaimida	unassigned
TS6.SSU587229	Enoplea; Enoplida; Oncholaimoidea; Oncholaimidae	Oncholaimidae
HE6.SSU372021	Chromadorea; Monhysterida	unassigned
