# Supplementary material for: Taxonomy assignment approach determines the efficiency of identification of OTUs in marine nematodes
Source: R Soc Open Sci. 2017 Aug 16;4(8):170315. doi: 10.1098/rsos.170315 (PMC5579096; doi:10.1098/rsos.170315)
Supplement: Supplementary Table 6 [file rsos170315supp9.pdf]

Supplementary file for the article:

Holovachov O, Haenel Q, Bourlat SJ, Jondelius U. Taxonomy assignment approach determines the efficiency of identification of OTUs in marine nematodes. *Royal Society Open Science.*

Supplementary Table 6. Results of tree-based taxonomy assignment using complete and partitioned query dataset.

OTU ID	Family identification (complete)	bootstrap %	Family identification (partitioned)	bootstrap %
HE1.SSU848264	unassigned	–	unassigned	–
HE1.SSU850987	unassigned	–	unassigned	–
HE1.SSU856624	Oxystominidae	90	Oxystominidae	93
HE1.SSU856738	unassigned	–	unassigned	–
HE1.SSU858060	unassigned	–	Chromadoridae	92
HE1.SSU867071	Selachinematidae	86	unassigned	–
HE2.SSU637072	unassigned	–	unassigned	–
HE2.SSU637135	unassigned	–	unassigned	–
HE2.SSU644966	unassigned	–	unassigned	–
HE2.SSU654005	Rhabdodemaniidae	100	Rhabdodemaniidae	100
HE2.SSU655107	Xyalidae	70	Xyalidae	76
HE2.SSU659506	unassigned	–	Chromadoridae	91
HE3.SSU110275	Enoplidae	100	Enoplidae	100
HE3.SSU117415	unassigned	–	unassigned	–
HE3.SSU118424	unassigned	–	Oxystominidae	72
HE3.SSU124287	Thoracostomopsidae	84	Thoracostomopsidae	100
HE3.SSU124998	unassigned	–	unassigned	–
HE4.SSU913283	unassigned	–	unassigned	–
HE5.SSU181724	unassigned	–	unassigned	–
HE5.SSU188855	Enchelidiidae	100	Enchelidiidae	100
HE6.SSU355777	unassigned	–	unassigned	–
HE6.SSU358048	unassigned	–	unassigned	–
HE6.SSU360897	Desmodoridae	77	unassigned	–
HE6.SSU361449	Ironidae	90	Ironidae	93
HE6.SSU365256	unassigned	–	unassigned	–
HE6.SSU368318	unassigned	–	unassigned	–
HE6.SSU370544	Xyalidae	70	Xyalidae	99
HE6.SSU378839	Microlaimidae	99	Microlaimidae	98
HE6.SSU383414	unassigned	–	Comesomatidae	90
HE6.SSU383888	unassigned	–	Chromadoridae	86
HE7.SSU232624	Leptolaimidae	82	Leptolaimidae	92
HE7.SSU256492	unassigned	–	unassigned	–
HE8.SSU829972	Anticomidae	100	Anticomidae	100

OTU ID	Family identification (complete)	bootstrap %	Family identification (partitioned)	bootstrap %
HE8.SSU843570	unassigned	–	unassigned	–
HE9.SSU305678	Xyalidae	70	unassigned	–
HF1.SSU759758	Camacolaimidae	99	Camacolaimidae	100
HF1.SSU763392	unassigned	–	unassigned	–
HF1.SSU764346	unassigned	–	unassigned	–
HF1.SSU774294	unassigned	–	unassigned	–
HF1.SSU779114	Axonolaimidae	82	Axonolaimidae	75
HF1.SSU780927	unassigned	–	unassigned	–
HF2.SSU192072	Chromadoridae	91	Chromadoridae	100
HF2.SSU204352	Leptolaimidae	82	unassigned	–
HF2.SSU205129	unassigned	–	unassigned	–
HF2.SSU208147	Selachinematidae	86	Selachinematidae	88
HF2.SSU210357	Enchelidiidae	85	Enchelidiidae	85
HF3.SSU989895	unassigned	–	Camacolaimidae	77
HF3.SSU990962	unassigned	–	unassigned	–
HF4.SSU606153	unassigned	–	unassigned	–
HF4.SSU614317	unassigned	–	unassigned	–
HF4.SSU619471	Microlaimidae	99	Microlaimidae	100
HF4.SSU620879	unassigned	–	Chromadoridae	100
HF4.SSU622464	unassigned	–	unassigned	–
HF4.SSU624085	unassigned	–	unassigned	–
HF4.SSU625424	unassigned	–	Comesomatidae	95
HF4.SSU628562	unassigned	–	unassigned	–
HF4.SSU631524	Leptolaimidae	94	Leptolaimidae	100
HF4.SSU632264	Diplopeltidae	100	Diplopeltidae	100
HF4.SSU635045	Chromadoridae	91	Chromadoridae	100
HF5.SSU991188	unassigned	–	Oncholaimidae	100
HF5.SSU995414	unassigned	–	unassigned	–
HF6.SSU329881	Desmodoridae	77	Desmodoridae	85
HF6.SSU338435	unassigned	–	Chromadoridae	97
HF6.SSU338739	unassigned	–	unassigned	–
HF7.SSU385021	unassigned	–	Chromadoridae	97
HF7.SSU390110	unassigned	–	unassigned	–
HF7.SSU398053	unassigned	–	unassigned	–
HF7.SSU407024	unassigned	–	unassigned	–
HF7.SSU407761	unassigned	–	unassigned	–
HF7.SSU409331	unassigned	–	unassigned	–
HF8.SSU795426	unassigned	–	unassigned	–
HF9.SSU14048	unassigned	–	unassigned	–
HF9.SSU14296	unassigned	–	unassigned	–
HF9.SSU17250	unassigned	–	Thoracostomopsidae	89

OTU ID	Family identification (complete)	bootstrap %	Family identification (partitioned)	bootstrap %
HF9.SSU17844	unassigned	–	unassigned	–
HF9.SSU18227	unassigned	–	unassigned	–
HF9.SSU19963	unassigned	–	unassigned	–
HF9.SSU20251	Microlaimidae	99	Microlaimidae	97
HF9.SSU22538	unassigned	–	unassigned	–
TF1.SSU676746	Ceramonematidae	79	Ceramonematidae	80
TF1.SSU677162	unassigned	–	unassigned	–
TF1.SSU681557	Oxystominidae	90	Oxystominidae	89
TF1.SSU688192	Linhomoeidae	96	Linhomoeidae	89
TF1.SSU692690	Selachinematidae	86	Selachinematidae	95
TF1.SSU694267	unassigned	–	unassigned	–
TF1.SSU694751	unassigned	–	Chromadoridae	98
TF1.SSU698227	unassigned	–	unassigned	–
TF1.SSU700188	Cyartonematidae	94	Cyartonematidae	89
TF1.SSU703579	unassigned	–	unassigned	–
TF1.SSU710679	unassigned	–	Cyatholaimidae	95
TF1.SSU734804	unassigned	–	unassigned	–
TF3.SSU956521	unassigned	–	unassigned	–
TF3.SSU960449	unassigned	–	unassigned	–
TF3.SSU966338	Xyalidae	70	Xyalidae	78
TF4.SSU144249	unassigned	–	Cyatholaimidae	74
TF4.SSU150234	unassigned	–	unassigned	–
TF5.SSU410031	unassigned	–	unassigned	–
TF5.SSU419519	unassigned	–	unassigned	–
TF5.SSU430294	Xyalidae	70	Xyalidae	78
TF5.SSU437076	Comesomatidae	93	unassigned	–
TF5.SSU444034	Rhabdodemaniidae	100	Rhabdodemaniidae	98
TF5.SSU446087	unassigned	–	Tarvaiidae	91
TF5.SSU453472	Oxystominidae	85	Oxystominidae	90
TF5.SSU457543	Oxystominidae	85	Oxystominidae	94
TF5.SSU459305	Oncholaimidae	75	Oncholaimidae	74
TF5.SSU466315	Xyalidae	70	Xyalidae	100
TF6.SSU33463	Oxystominidae	99	Oxystominidae	99
TF6.SSU33935	unassigned	–	unassigned	–
TF6.SSU36442	unassigned	–	unassigned	–
TF6.SSU37421	unassigned	–	unassigned	–
TF6.SSU41803	unassigned	–	Xyalidae	93
TF6.SSU47996	Enchelidiidae	75	Enchelidiidae	91
TF6.SSU48167	unassigned	–	Comesomatidae	90
TF6.SSU53456	Oncholaimidae	71	Oncholaimidae	100
TF6.SSU54250	Microlaimidae	99	Microlaimidae	100

OTU ID	Family identification (complete)	bootstrap %	Family identification (partitioned)	bootstrap %
TF6.SSU58877	unassigned	–	Tarvaiidae	91
TF6.SSU74955	unassigned	–	unassigned	–
TF6.SSU82210	unassigned	–	unassigned	–
TF6.SSU84268	unassigned	–	unassigned	–
TF6.SSU98667	unassigned	–	unassigned	–
TS1.SSU270885	unassigned	–	unassigned	–
TS1.SSU284163	unassigned	–	unassigned	–
TS2.SSU821962	Tripyloididae	100	Tripyloididae	100
TS2.SSU823349	Tripyloididae	100	Tripyloididae	100
TS3.SSU475561	unassigned	–	unassigned	–
TS3.SSU489684	Desmoscolecidae	84	unassigned	–
TS3.SSU503133	Tripyloididae	100	Tripyloididae	100
TS3.SSU508400	unassigned	–	unassigned	–
TS4.SSU543236	Oxystominidae	90	Oxystominidae	93
TS4.SSU544032	unassigned	–	unassigned	–
TS5.SSU874117	Oxystominidae	100	Oxystominidae	100
TS5.SSU875407	Comesomatidae	93	unassigned	–
TS5.SSU881546	Xyalidae	70	unassigned	–
TS5.SSU900338	Leptolaimidae	94	Leptolaimidae	99
TS5.SSU901243	Ironidae	81	Ironidae	100
TS6.SSU559765	Tripyloididae	100	Tripyloididae	100
TS6.SSU570763	unassigned	–	unassigned	–
TS6.SSU587229	Oncholaimidae	75	Oncholaimidae	100
HE6.SSU372021	Monhysteridae	82	Monhysteridae	92
