# Supplementary material for: Taxonomy assignment approach determines the efficiency of identification of OTUs in marine nematodes
Source: R Soc Open Sci. 2017 Aug 16;4(8):170315. doi: 10.1098/rsos.170315 (PMC5579096; doi:10.1098/rsos.170315)
Supplement: Supplementary Table 7 [file rsos170315supp10.pdf]

Supplementary file for the article:

Holovachov O, Haenel Q, Bourlat SJ, Jondelius U. Taxonomy assignment approach determines the efficiency of identification of OTUs in marine nematodes. *Royal Society Open Science.*

Supplementary Table 7. Results of phylogeny-based taxonomy assignment using mothur and PaPaRa-based alignments.

OTU ID	Family identification (mothur)	likelihood %	Family identification (PaPaRa)	likelihood %
HE1.SSU848264	Cyatholaimidae	100	Cyatholaimidae	100
HE1.SSU850987	unassigned	–	unassigned	–
HE1.SSU856624	Oxystominidae	100	Oxystominidae	100
HE1.SSU856738	Microlaimidae	100	Microlaimidae	100
HE1.SSU858060	Chromadoridae	100	Chromadoridae	100
HE1.SSU867071	Selachinematidae	100	Selachinematidae	100
HE2.SSU637072	unassigned	–	unassigned	–
HE2.SSU637135	Chromadoridae	100	Chromadoridae	100
HE2.SSU644966	Cyatholaimidae	100	Cyatholaimidae	100
HE2.SSU654005	Rhabdodemaniidae	NA	Rhabdodemaniidae	NA
HE2.SSU655107	Xyalidae	97	Xyalidae	97
HE2.SSU659506	Chromadoridae	100	Chromadoridae	100
HE3.SSU110275	Enoplidae	100	Enoplidae	100
HE3.SSU117415	Cyatholaimidae	100	Cyatholaimidae	100
HE3.SSU118424	Oxystominidae	100	Oxystominidae	100
HE3.SSU124287	Thoracostomopsidae	100	Thoracostomopsidae	100
HE3.SSU124998	unassigned	–	unassigned	–
HE4.SSU913283	unassigned	–	unassigned	–
HE5.SSU181724	unassigned	–	unassigned	–
HE5.SSU188855	Enchelidiidae	94	Enchelidiidae	94
HE6.SSU355777	unassigned	–	unassigned	–
HE6.SSU358048	Chromadoridae	100	Chromadoridae	100
HE6.SSU360897	unassigned	–	unassigned	–
HE6.SSU361449	Ironidae	100	Ironidae	100
HE6.SSU365256	Desmodoridae	72	Desmodoridae	72
HE6.SSU368318	unassigned	–	unassigned	–
HE6.SSU370544	Xyalidae	97	Xyalidae	97
HE6.SSU378839	Microlaimidae	100	Microlaimidae	100
HE6.SSU383414	Comesomatidae	99	Comesomatidae	99
HE6.SSU383888	Chromadoridae	100	Chromadoridae	100
HE7.SSU232624	Leptolaimidae	99	Leptolaimidae	99
HE7.SSU256492	Chromadoridae	100	Chromadoridae	100
HE8.SSU829972	Anticomidae	100	Anticomidae	100

OTU ID	Family identification (mothur)	likelihood %	Family identification (PaPaRa)	likelihood %
HE8.SSU843570	Chromadoridae	100	Chromadoridae	100
HE9.SSU305678	Xyalidae	97	Xyalidae	97
HF1.SSU759758	Camacolaimidae	100	Camacolaimidae	100
HF1.SSU763392	Cyatholaimidae	100	Cyatholaimidae	100
HF1.SSU764346	Cyatholaimidae	100	Cyatholaimidae	100
HF1.SSU774294	Mermithidae	100	Mermithidae	100
HF1.SSU779114	Axonolaimidae	100	Axonolaimidae	100
HF1.SSU780927	unassigned	–	unassigned	–
HF2.SSU192072	Chromadoridae	100	Chromadoridae	100
HF2.SSU204352	unassigned	–	unassigned	–
HF2.SSU205129	Chromadoridae	100	Chromadoridae	100
HF2.SSU208147	Selachinematidae	100	Selachinematidae	100
HF2.SSU210357	Enchelidiidae	94	Enchelidiidae	94
HF3.SSU989895	Camacolaimidae	100	Camacolaimidae	100
HF3.SSU990962	Chromadoridae	100	Chromadoridae	100
HF4.SSU606153	Chromadoridae	100	Chromadoridae	100
HF4.SSU614317	unassigned	–	unassigned	–
HF4.SSU619471	Microlaimidae	100	Microlaimidae	100
HF4.SSU620879	Chromadoridae	100	Chromadoridae	100
HF4.SSU622464	unassigned	–	unassigned	–
HF4.SSU624085	unassigned	–	unassigned	–
HF4.SSU625424	Comesomatidae	99	Comesomatidae	99
HF4.SSU628562	Desmodoridae	72	Desmodoridae	72
HF4.SSU631524	Leptolaimidae	99	Leptolaimidae	99
HF4.SSU632264	Diplopeltidae	100	Diplopeltidae	100
HF4.SSU635045	Chromadoridae	100	Chromadoridae	100
HF5.SSU991188	Oncholaimidae	100	Oncholaimidae	100
HF5.SSU995414	Ironidae	100	Ironidae	100
HF6.SSU329881	Desmodoridae	72	Desmodoridae	72
HF6.SSU338435	Chromadoridae	100	Chromadoridae	100
HF6.SSU338739	unassigned	–	unassigned	–
HF7.SSU385021	Chromadoridae	100	Chromadoridae	100
HF7.SSU390110	unassigned	–	unassigned	–
HF7.SSU398053	Camacolaimidae	100	Camacolaimidae	100
HF7.SSU407024	Achromadoridae	97	Achromadoridae	97
HF7.SSU407761	Linhomoeidae	99	Linhomoeidae	99
HF7.SSU409331	unassigned	–	unassigned	–
HF8.SSU795426	unassigned	–	unassigned	–
HF9.SSU14048	Microlaimidae	100	Microlaimidae	100
HF9.SSU14296	Selachinematidae	100	Selachinematidae	100
HF9.SSU17250	Thoracostomopsidae	100	Thoracostomopsidae	100

OTU ID	Family identification (mothur)	likelihood %	Family identification (PaPaRa)	likelihood %
HF9.SSU17844	unassigned	–	unassigned	–
HF9.SSU18227	Chromadoridae	100	Chromadoridae	100
HF9.SSU19963	unassigned	–	unassigned	–
HF9.SSU20251	Microlaimidae	100	Microlaimidae	100
HF9.SSU22538	unassigned	–	unassigned	–
TF1.SSU676746	Ceramonematidae	100	Ceramonematidae	100
TF1.SSU677162	unassigned	–	unassigned	–
TF1.SSU681557	Oxystominidae	100	Oxystominidae	100
TF1.SSU688192	unassigned	–	unassigned	–
TF1.SSU692690	Selachinematidae	100	Selachinematidae	100
TF1.SSU694267	Desmoscolecidae	99	Desmoscolecidae	99
TF1.SSU694751	Chromadoridae	100	Chromadoridae	100
TF1.SSU698227	Benthimermithidae	NA	Benthimermithidae	NA
TF1.SSU700188	Cyartonematidae	NA	Cyartonematidae	NA
TF1.SSU703579	unassigned	–	unassigned	–
TF1.SSU710679	Cyatholaimidae	100	Cyatholaimidae	100
TF1.SSU734804	Siphonolaimidae	99	Siphonolaimidae	99
TF3.SSU956521	unassigned	–	unassigned	–
TF3.SSU960449	Desmoscolecidae	99	Desmoscolecidae	99
TF3.SSU966338	Xyalidae	97	Xyalidae	97
TF4.SSU144249	Cyatholaimidae	100	Cyatholaimidae	100
TF4.SSU150234	Desmodoridae	99	Desmodoridae	99
TF5.SSU410031	unassigned	–	unassigned	–
TF5.SSU419519	Desmoscolecidae	99	Desmoscolecidae	99
TF5.SSU430294	Xyalidae	97	Xyalidae	97
TF5.SSU437076	Comesomatidae	99	Comesomatidae	99
TF5.SSU444034	Rhabdodemaniidae	NA	Rhabdodemaniidae	NA
TF5.SSU446087	Tarvaiidae	NA	Tarvaiidae	NA
TF5.SSU453472	Oxystominidae	100	Oxystominidae	100
TF5.SSU457543	Oxystominidae	100	Oxystominidae	100
TF5.SSU459305	Oncholaimidae	100	Oncholaimidae	100
TF5.SSU466315	Xyalidae	100	Xyalidae	100
TF6.SSU33463	Oxystominidae	100	Oxystominidae	100
TF6.SSU33935	unassigned	–	unassigned	–
TF6.SSU36442	Desmoscolecidae	99	Desmoscolecidae	99
TF6.SSU37421	unassigned	–	unassigned	–
TF6.SSU41803	Xyalidae	97	Xyalidae	97
TF6.SSU47996	Enchelidiidae	90	Enchelidiidae	90
TF6.SSU48167	Comesomatidae	99	Comesomatidae	99
TF6.SSU53456	Oncholaimidae	81	Oncholaimidae	81
TF6.SSU54250	Microlaimidae	100	Microlaimidae	100

OTU ID	Family identification (mothur)	likelihood %	Family identification (PaPaRa)	likelihood %
TF6.SSU58877	Tarvaiidae	100	Tarvaiidae	100
TF6.SSU74955	Cyatholaimidae	100	Cyatholaimidae	100
TF6.SSU82210	unassigned	–	unassigned	–
TF6.SSU84268	unassigned	–	unassigned	–
TF6.SSU98667	unassigned	–	unassigned	–
TS1.SSU270885	Desmoscolecidae	99	Desmoscolecidae	99
TS1.SSU284163	Desmoscolecidae	99	Desmoscolecidae	99
TS2.SSU821962	Tripyloididae	100	Tripyloididae	100
TS2.SSU823349	Tripyloididae	100	Tripyloididae	100
TS3.SSU475561	unassigned	–	unassigned	–
TS3.SSU489684	Desmoscolecidae	99	Desmoscolecidae	99
TS3.SSU503133	Tripyloididae	100	Tripyloididae	100
TS3.SSU508400	unassigned	–	unassigned	–
TS4.SSU543236	Oxystominidae	100	Oxystominidae	100
TS4.SSU544032	Desmoscolecidae	99	Desmoscolecidae	99
TS5.SSU874117	Oxystominidae	100	Oxystominidae	100
TS5.SSU875407	Comesomatidae	99	Comesomatidae	99
TS5.SSU881546	Xyalidae	97	Xyalidae	97
TS5.SSU900338	Leptolaimidae	99	Leptolaimidae	99
TS5.SSU901243	Ironidae	100	Ironidae	100
TS6.SSU559765	Tripyloididae	100	Tripyloididae	100
TS6.SSU570763	unassigned	–	unassigned	–
TS6.SSU587229	Oncholaimidae	100	Oncholaimidae	100
HE6.SSU372021	Monhysteridae	100	Monhysteridae	100
