# Supplementary material for: Taxonomy assignment approach determines the efficiency of identification of OTUs in marine nematodes
Source: R Soc Open Sci. 2017 Aug 16;4(8):170315. doi: 10.1098/rsos.170315 (PMC5579096; doi:10.1098/rsos.170315)
Supplement: Supplementary Table 8 [file rsos170315supp11.pdf]

Supplementary file for the article:

Holovachov O, Haenel Q, Bourlat SJ, Jondelius U. Taxonomy assignment approach determines the efficiency of identification of OTUs in marine nematodes. *Royal Society Open Science.*

Supplementary Table 8. Comparison of the results produced by different taxonomy assignment approaches and distribution (presence vs. absence) of different OTUs among study sites (HE – Hållö, flotation with $MgCl_2$; HF – Hållö, flotation with H_2O; TS – Telekabeln, syphoning; TF – Telekabeln, flotation with H_2O).

	Distribution				BLASTN	CREST	TREE-C	TREE-P	EPA (BOTH)
OTU ID	HE	HF	TS	TF	52/139	26/139	56/139	67/139	105/139
HE1.SSU848264	*	*	–	–	Cyatholaimidae				Cyatholaimidae
HE1.SSU850987	*	*	–	–					
HE1.SSU856624	*	–	–	*			Oxystominidae	Oxystominidae	Oxystominidae
HE1.SSU856738	*	*	–	–	Microlaimidae	Microlaimidae			Microlaimidae
HE1.SSU858060	*	–	–	–				Chromadoridae	Chromadoridae
HE1.SSU867071	*	–	–	–			Selachinematidae		Selachinematidae
HE2.SSU637072	*	*	–	–					
HE2.SSU637135	*	*	–	–		Chromadoridae			Chromadoridae
HE2.SSU644966	*	–	–	–	Cyatholaimidae				Cyatholaimidae
HE2.SSU654005	*	*	–	–			Rhabdodemaniidae	Rhabdodemaniidae	Rhabdodemaniidae
HE2.SSU655107	*	*	–	–			Xyalidae	Xyalidae	Xyalidae
HE2.SSU659506	*	*	*	*	Chromadoridae			Chromadoridae	Chromadoridae
HE3.SSU110275	*	*	–	–	Enoplidae	Enoplidae	Enoplidae	Enoplidae	Enoplidae
HE3.SSU117415	*	–	–	*	Cyatholaimidae				Cyatholaimidae
HE3.SSU118424	*	–	–	–				Oxystominidae	Oxystominidae

	Distribution				BLASTN	CREST	TREE-C	TREE-P	EPA (BOTH)
OTU ID	HE	HF	TS	TF	92/139	26/139	54/139	67/139	105/139
HE3.SSU124287	*	*	–	–	Thoracostomopsidae	Thoracostomopsidae	Thoracostomopsidae	Thoracostomopsidae	Thoracostomopsidae
HE3.SSU124998	*	–	–	–					
HE4.SSU913283	*	–	–	–					
HE5.SSU181724	*	*	–	–					
HE5.SSU188855	*	*	*	–			Enchelidiidae	Enchelidiidae	Enchelidiidae
HE6.SSU355777	*	*	–	–	Achromadoridae				
HE6.SSU358048	*	–	–	–	Chromadoridae				Chromadoridae
HE6.SSU360897	*	–	–	–			Desmodoridae		
HE6.SSU361449	*	*	–	–	Ironidae		Ironidae	Ironidae	Ironidae
HE6.SSU365256	*	–	–	–					Desmodoridae
HE6.SSU368318	*	*	–	–		Desmodoridae			
HE6.SSU370544	*	–	–	–		Xyalidae	Xyalidae	Xyalidae	Xyalidae
HE6.SSU378839	*	–	–	–			Microlaimidae	Microlaimidae	Microlaimidae
HE6.SSU383414	*	–	–	–	Comesomatidae			Comesomatidae	Comesomatidae
HE6.SSU383888	*	*	–	*	Chromadoridae			Chromadoridae	Chromadoridae
HE7.SSU232624	*	*	–	*			Leptolaimidae	Leptolaimidae	Leptolaimidae
HE7.SSU256492	*	*	*	*	Chromadoridae				Chromadoridae
HE8.SSU829972	*	*	*	*			Anticomidae	Anticomidae	Anticomidae
HE8.SSU843570	*	*	–	–	Chromadoridae				Chromadoridae
HE9.SSU305678	*	*	–	*			Xyalidae		Xyalidae
HF1.SSU759758	–	*	–	–		Leptolaimidae	Camacolaimidae	Camacolaimidae	Camacolaimidae
HF1.SSU763392	*	*	–	–	Cyatholaimidae				Cyatholaimidae
HF1.SSU764346	*	*	*	*	Cyatholaimidae				Cyatholaimidae
HF1.SSU774294	*	*	–	–	Mermithidae				Mermithidae
HF1.SSU779114	*	*	*	*		Axonolaimidae	Axonolaimidae	Axonolaimidae	Axonolaimidae

	Distribution				BLASTN	CREST	TREE-C	TREE-P	EPA (BOTH)
OTU ID	HE	HF	TS	TF	92/139	26/139	54/139	67/139	105/139
HF1.SSU780927	–	*	*	–					
HF2.SSU192072	–	*	–	–		Chromadoridae	Chromadoridae	Chromadoridae	Chromadoridae
HF2.SSU204352	–	*	–	–			Leptolaimidae		
HF2.SSU205129	*	*	–	–					Chromadoridae
HF2.SSU208147	*	*	–	*			Selachinematidae	Selachinematidae	Selachinematidae
HF2.SSU210357	–	*	–	–	Enchelidiidae		Enchelidiidae	Enchelidiidae	Enchelidiidae
HF3.SSU989895	*	*	–	–				Camacolaimidae	Camacolaimidae
HF3.SSU990962	–	*	–	–	Chromadoridae				Chromadoridae
HF4.SSU606153	*	*	*	*	Chromadoridae	Chromadoridae			Chromadoridae
HF4.SSU614317	*	*	–	*	Comesomatidae	Comesomatidae			
HF4.SSU619471	*	*	–	–	Microlaimidae		Microlaimidae	Microlaimidae	Microlaimidae
HF4.SSU620879	–	*	–	–	Chromadoridae			Chromadoridae	Chromadoridae
HF4.SSU622464	–	*	–	–	Plectidae				
HF4.SSU624085	*	*	–	–					
HF4.SSU625424	*	*	–	–				Comesomatidae	Comesomatidae
HF4.SSU628562	–	*	–	–					Desmodoridae
HF4.SSU631524	–	*	–	–		Leptolaimidae	Leptolaimidae	Leptolaimidae	Leptolaimidae
HF4.SSU632264	–	*	–	–			Diplopeltidae	Diplopeltidae	Diplopeltidae
HF4.SSU635045	*	*	–	–			Chromadoridae	Chromadoridae	Chromadoridae
HF5.SSU991188	*	*	–	–				Oncholaimidae	Oncholaimidae
HF5.SSU995414	–	*	–	–	Rhabdolaimidae	Ironidae			Ironidae
HF6.SSU329881	*	*	*	*		Desmodoridae	Desmodoridae	Desmodoridae	Desmodoridae
HF6.SSU338435	–	*	–	–				Chromadoridae	Chromadoridae
HF6.SSU338739	*	*	–	–					

	Distribution				BLASTN	CREST	TREE-C	TREE-P	EPA (BOTH)
OTU ID	HE	HF	TS	TF	92/139	26/139	54/139	67/139	105/139
HF7.SSU385021	–	*	–	–	Chromadoridae			Chromadoridae	Chromadoridae
HF7.SSU390110	*	*	*	*					
HF7.SSU398053	–	*	–	–					Camacolaimidae
HF7.SSU407024	*	*	–	*					Achromadoridae
HF7.SSU407761	*	*	–	–					Linhomoeidae
HF7.SSU409331	–	*	–	–					
HF8.SSU795426	–	*	–	–	Cyatholaimidae				
HF9.SSU14048	–	*	–	–					Microlaimidae
HF9.SSU14296	–	*	–	–					Selachinematidae
HF9.SSU17250	–	*	–	–	Thoracostomopsidae	Thoracostomopsidae		Thoracostomopsidae	Thoracostomopsidae
HF9.SSU17844	*	*	–	–					
HF9.SSU18227	–	*	–	–	Chromadoridae				Chromadoridae
HF9.SSU19963	–	*	–	–					
HF9.SSU20251	*	*	*	*	Microlaimidae	Microlaimidae	Microlaimidae	Microlaimidae	Microlaimidae
HF9.SSU22538	–	*	–	–	Mermithidae				
TF1.SSU676746	–	–	–	*			Ceramonematidae	Ceramonematidae	Ceramonematidae
TF1.SSU677162	–	–	–	*					
TF1.SSU681557	–	–	*	*			Oxystominidae	Oxystominidae	Oxystominidae
TF1.SSU688192	–	–	–	*	Linhomoeidae		Linhomoeidae	Linhomoeidae	
TF1.SSU692690	–	–	–	*	Selachinematidae		Selachinematidae	Selachinematidae	Selachinematidae
TF1.SSU694267	–	–	–	*					Desmoscolecidae
TF1.SSU694751	–	–	–	*	Chromadoridae			Chromadoridae	Chromadoridae
TF1.SSU698227	–	–	–	*	Teratocephalidae				Benthimermithidae
TF1.SSU700188	–	–	–	*	Linhomoeidae		Cyartonematidae	Cyartonematidae	Cyartonematidae

	Distribution				BLASTN	CREST	TREE-C	TREE-P	EPA (BOTH)
OTU ID	HE	HF	TS	TF	92/139	26/139	54/139	67/139	105/139
TF1.SSU703579	–	–	–	*	Siphonolaimidae				
TF1.SSU710679	*	*	*	*	Cyatholaimidae	Cyatholaimidae		Cyatholaimidae	Cyatholaimidae
TF1.SSU734804	–	–	–	*	Siphonolaimidae				Siphonolaimidae
TF3.SSU956521	–	–	–	*	Comesomatidae				
TF3.SSU960449	–	–	–	*					Desmoscolecidae
TF3.SSU966338	*	*	*	*			Xyalidae	Xyalidae	Xyalidae
TF4.SSU144249	*	*	*	*				Cyatholaimidae	Cyatholaimidae
TF4.SSU150234	–	–	*	*	Desmodoridae				Desmodoridae
TF5.SSU410031	–	–	–	*					
TF5.SSU419519	*	*	*	*					Desmoscolecidae
TF5.SSU430294	*	*	*	*			Xyalidae	Xyalidae	Xyalidae
TF5.SSU437076	–	–	*	*	Comesomatidae	Comesomatidae	Comesomatidae		Comesomatidae
TF5.SSU444034	*	*	*	*			Rhabdodemaniidae	Rhabdodemaniidae	Rhabdodemaniidae
TF5.SSU446087	–	–	–	*				Tarvaiidae	Tarvaiidae
TF5.SSU453472	–	–	–	*			Oxystominidae	Oxystominidae	Oxystominidae
TF5.SSU457543	–	–	*	*			Oxystominidae	Oxystominidae	Oxystominidae
TF5.SSU459305	–	–	–	*	Oncholaimidae	Oncholaimidae	Oncholaimidae	Oncholaimidae	Oncholaimidae
TF5.SSU466315	–	–	*	*			Xyalidae	Xyalidae	Xyalidae
TF6.SSU33463	*	*	*	*			Oxystominidae	Oxystominidae	Oxystominidae
TF6.SSU33935	*	–	–	*					
TF6.SSU36442	–	–	–	*					Desmoscolecidae
TF6.SSU37421	–	–	*	*		Desmodoridae			
TF6.SSU41803	–	–	–	*		Xyalidae		Xyalidae	Xyalidae
TF6.SSU47996	–	–	*	*	Oncholaimidae	Enchelidiidae	Enchelidiidae	Enchelidiidae	Enchelidiidae

	Distribution				BLASTN	CREST	TREE-C	TREE-P	EPA (BOTH)
OTU ID	HE	HF	TS	TF	92/139	26/139	54/139	67/139	105/139
TF6.SSU48167	*	*	*	*	Comesomatidae	Comesomatidae		Comesomatidae	Comesomatidae
TF6.SSU53456	*	*	*	*		Oncholaimidae	Oncholaimidae	Oncholaimidae	Oncholaimidae
TF6.SSU54250	–	–	*	*	Microlaimidae		Microlaimidae	Microlaimidae	Microlaimidae
TF6.SSU58877	–	–	–	*				Tarvaiidae	Tarvaiidae
TF6.SSU74955	–	–	–	*	Cyatholaimidae				Cyatholaimidae
TF6.SSU82210	–	–	*	*					
TF6.SSU84268	–	–	–	*					
TF6.SSU98667	–	–	–	*					
TS1.SSU270885	–	–	*	–					Desmoscolecidae
TS1.SSU284163	–	–	*	–					Desmoscolecidae
TS2.SSU821962	*	*	*	*	Tripyloididae		Tripyloididae	Tripyloididae	Tripyloididae
TS2.SSU823349	*	–	*	*			Tripyloididae	Tripyloididae	Tripyloididae
TS3.SSU475561	–	–	*	–	Siphonolaimidae				
TS3.SSU489684	*	*	*	*			Desmoscolecidae		Desmoscolecidae
TS3.SSU503133	–	–	*	–	Tripyloididae		Tripyloididae	Tripyloididae	Tripyloididae
TS3.SSU508400	–	–	*	*					
TS4.SSU543236	–	–	*	–			Oxystominidae	Oxystominidae	Oxystominidae
TS4.SSU544032	–	*	*	*					Desmoscolecidae
TS5.SSU874117	–	–	*	*			Oxystominidae	Oxystominidae	Oxystominidae
TS5.SSU875407	–	–	*	*	Comesomatidae	Comesomatidae	Comesomatidae		Comesomatidae
TS5.SSU881546	–	–	*	–			Xyalidae		Xyalidae
TS5.SSU900338	–	–	*	–			Leptolaimidae	Leptolaimidae	Leptolaimidae
TS5.SSU901243	–	–	*	*			Ironidae	Ironidae	Ironidae

	Distribution				BLASTN	CREST	TREE-C	TREE-P	EPA (BOTH)
OTU ID	HE	HF	TS	TF	92/139	26/139	54/139	67/139	105/139
TS6.SSU559765	–	–	*	–			Tripyloididae	Tripyloididae	Tripyloididae
TS6.SSU570763	–	–	*	–					
TS6.SSU587229	*	*	*	*	Oncholaimidae	Oncholaimidae	Oncholaimidae	Oncholaimidae	Oncholaimidae
HE6.SSU372021	*	–	–	–			Monhysteridae	Monhysteridae	Monhysteridae
