# Supplementary material for: Taxonomy assignment approach determines the efficiency of identification of OTUs in marine nematodes
Source: R Soc Open Sci. 2017 Aug 16;4(8):170315. doi: 10.1098/rsos.170315 (PMC5579096; doi:10.1098/rsos.170315)
Supplement: Supplementary Table 9 [file rsos170315supp12.pdf]

Supplementary file for the article:

Holovachov O, Haenel Q, Bourlat SJ, Jondelius U. Taxonomy assignment approach determines the efficiency of identification of OTUs in marine nematodes. *Royal Society Open Science.*

Supplementary Table 9. Number of taxa (species/OTUs) per family recovered in morphology-based identification and best scoring taxonomy assignment approach (EPA) grouped per each site and extraction method.

	Hållö				Telekabeln			
	Flotation (MgCl2)		Flotation (H_2O)		Siphoning		Flotation (H_20)	
	morphology based	barcoding	morphology based	barcoding	morphology based	barcoding	morphology based	barcoding
**ORDER ENOPLIDA**								
Enoplidae	1	1	1	1	0	0	0	0
Thoracostomopsidae	2	1	2	2	1	0	1	0
Phanodermatidae	0	0	1	0	0	0	0	0
Anticomidae	1	1	1	1	1	1	1	1
Oncholaimidae	3	3	3	3	4	2	3	3
Enchelidiidae	2	1	2	2	2	2	2	1
Leptosomatidae	0	0	1	0	0	0	0	0
Ironidae	0	1	0	2	1	1	1	1
Oxystominidae	4	3	5	1	15	5	15	6
Tripyloididae	0	2	1	1	2	4	2	2
Xennellidae	1	NA	1	NA	0	NA	0	NA
Trefusiidae	0	0	0	0	0	0	1	0
**ORDER MERMITHIDA**								
Mermithidae	0	1	0	1	0	0	0	0
**ORDER TRIPLONCHIDA**								
Pandolaimidae	0	NA	0	NA	1	NA	1	NA
Rhabdodemaniidae	1	2	1	2	1	1	1	1
**ORDER DESMOSCOLECIDA**								
Desmoscolecidae	3	2	3	3	4	5	4	6
Meyliidae	0	NA	2	NA	0	NA	0	NA
Cyartonematidae	0	0	0	0	0	0	1	1
**ORDER CHROMADORIDA**								
Achromadoridae	0	1	0	1	0	0	0	1
Chromadoridae	14	10	13	14	7	3	6	5
Cyatholaimidae	8	7	9	5	4	3	3	5
Selachinematidae	4	2	4	2	2	0	4	2

	Hållö				Telekabeln			
	Flotation (MgCl2)		Flotation (H_2O)		Siphoning		Flotation (H_20)	
	morphology based	barcoding	morphology based	barcoding	morphology based	barcoding	morphology based	barcoding
**ORDER DESMODORIDA**								
Desmodoridae	10	2	11	2	4	2	4	2
Epsilonematidae	2	0	2	0	0	0	0	0
Draconematidae	0	0	1	0	0	0	0	0
Microlaimidae	6	4	7	4	1	2	4	2
Monoposthiidae	1	0	1	0	1	0	0	0
Richtersiidae	0	NA	0	NA	1	NA	1	NA
**ORDER MONHYSTERIDA**								
Xyalidae	7	5	9	4	3	4	4	5
Sphaerolaimidae	0	0	0	0	3	0	3	0
Monhysteridae	1	1	1	0	0	0	0	0
Siphonolaimidae	1	0	1	0	0	0	1	1
Linhomoeidae	2	1	2	1	5	0	6	0
**ORDER ARAEOLAIMIDA**								
Comesomatidae	2	3	1	2	3	3	4	3
Axonolaimidae	3	1	2	1	2	1	2	1
Diplopeltidae	2	0	2	1	6	0	6	0
**ORDER PLECTIDA**								
Leptolaimidae	4	1	4	2	2	1	6	1
Camacolaimidae	2	1	4	3	2	0	4	0
Rhadinematidae	0	NA	0	NA	1	NA	1	NA
Ceramonematidae	0	0	0	0	1	0	2	1
Diplopeltoididae	1	NA	1	NA	1	NA	4	NA
Tarvaiidae	0	0	1	0	0	0	0	2
Tubolaimoididae	0	NA	1	NA	0	NA	0	NA
Paramicrolaimidae	0	NA	0	NA	1	NA	1	NA
Aegialoalaimidae	0	NA	0	NA	0	NA	1	NA
**ORDER BENTHIMERMITHIDA**								
Benthimermithidae	0	0	0	0	0	0	0	1
Unidentified taxa	1	14	11	17	8	7	21	13
