# Supplementary material for: Taxonomy assignment approach determines the efficiency of identification of OTUs in marine nematodes
Source: R Soc Open Sci. 2017 Aug 16;4(8):170315. doi: 10.1098/rsos.170315 (PMC5579096; doi:10.1098/rsos.170315)
Supplement: Supplementary Data 1 [file rsos170315supp13.pdf]

Supplementary file for the article:

Holovachov O, Haenel Q, Bourlat SJ, Jondelius U. Taxonomy assignment approach determines the efficiency of identification of OTUs in marine nematodes. *Royal Society Open Science.*

Supplementary Data 1. Nematode OTU sequences used in this study.

```
>HE1_SSU848264
ATGCATGTCTAAGCAAGAGCCTAGAAATGGTGAAGCCGCGAACAGCTCATTACAACAGCCATAGTTTATTGAATCGTCTCTCATATACT
TGGATACCTTTGGTAATTCTAGAGCTAATACACGCACCAAACTCTGAGCTGAGGCAAGGAGTGCATTTATTAGAACAAAACCAATGGGC
TTCGGCCCTGTTTTGGTGAATCTGAATAACTCAGTTGATCGCACAGTCTCGCACTGGCGACGTATCTTTCAAGTGTCTGCCTTATCAAC
TGTCGATGGTAGTTTATATGACTACCATGGTTGTAACGGGTAACGGAGAATAAGGGTTCGACTCCGGAGAGGGAGCCTGAGAAATGGCT
ACCACA
>HE1_SSU850987
ATGCGTGTCTAAGCACAAACTGATTTAAGGTGAAGCCGCGAATGGCTCATTACAACAGCCATAGTTTCTTGGATCTTACTTCTACTTGG
ATAACTGTAGTAATTCTAGAGCTAATACATGCGTCAAGCTTCAACCTCATGGAAGAAACACATTTATTAGATCAAAACCAATCGGGCTT
TGCTTGTTTGTTTGATGAATCTGAATAACTCTGCCAATTGCATGGTCTTTGCACCGGTGACATATCTTTCAAGTGTCTGCCTTATCAAC
TGTTGATGGTAGTTTATATGACTACCATGGTTGTAACGGGTAACGGAGAATCAGGGTTCGACTCCGGAGAGGGAGCCCGAGAAACGGCT
ACCACA
>HE1_SSU856624
ATGCATGTCTCAGCACACGCCAATGTATGGTAAAGCCGCGAATGGCTCATTACAACAGCCACCGTTTATTAGATCATCCTTCTTACTTG
GATAACTGTGGAAAAGCTAGAGCTAATACATGCTACAAGCTCTGACCTTACGGAAAGAGCGCATTTATTAGAACAAAACCAATCGGACT
TTGTCCGTCGTTTGGTGAATCTGAATAACTAAGCAGATCGCACGGTCTTAGAACCGGCGACATATCGTTCAAATGTCTGCCTTATCAAC
TTTCGATGGTAGTTTATGCGCCTACCATGGTTGTAACGGGTAACGAAGAATCAGGGTTTGATTTCGGAGAGGGAGCCTGAGAAACGGCT
ACCACA
>HE1_SSU856738
ATGCATGTCTAAGTACAAGCCTCTTTAAGGTGAAACCGCGAATGGCTCATTAAATCACACCTAATATACTGGATAGTATACAGTTACTT
GGATAACTGTGGTAATTCTAGAGCTAATACACGCATTCAAGTTCCGACGCAAGAAGGAACGCATTTATTAGAACTAAACCAATCGGGCT
TGCCCGTTGTTTGGTGAATCTGAATAACTCCGCAGATCGCATGGTCTAGCACCGGCGACATATCTTTCAAGTGTCTGCCTTATCAACTT
TCGATGGTAGTTTATGTGACTACCATGGTTGTTACGGGTAACGGAGAATTAGGGTTCGACTCCGGAGAGGGAGCCTGAGAAACGGCTAC
CACA
>HE1_SSU858060
ATGCATGTCTAAGCATAAACCGAATATGGTAAAGCCGCGAATGGCTCATTACAACAGCCATAGTTTATTGGATCTTAGAGTCCTACTTG
GATACCTGTGGTAATTCTAGAGCTAATACACGCAATCAAGCCCCAACCTTACGGGCGGGGCGCATTTATTAGAACAAAACCAATTGGCT
TCGGCCATAGATTGGTGAATCTGAATAACTACGCTGATCGCACGGGCTTGTCCCGGCGACGTATCCTTCCAAGGTGTGCCCTATCAACT
GTCGACTGTGGCATAGACGCCCACAGTGGTTTTGACGGGTAACGGGGAATCAGGGTTCGATTCCGGAGAGGGAGCCTGAGAAACGGCTA
CCACA
>HE1_SSU867071
ATGCATGTCTAAGTACAGGCCTCACTAAGGTGAAACCGCGAATGGCTCATTAAATCACACATGATTTATTCGATCATAAAATCCTACTT
GGATAACTGTGGTAATTCTAGAGCTAATACACGCAACTAAACTCCAACCTTGCGGAAGGAGTGCATTTATTAGAACAAAACCAATCGGC
TTCGGCCGTTACTTGGTGAATCTGAATAACTCAGTTGATCGCACGGTCTTTGTACTGGCGACATATCTTTCAAGTGTCTGCCTTATCAA
CTTTCGATGGTAGTTTATACGACTACCATGGTTGTTACGGGTAACGGAGAATTAGGGTTCGACTCCGGAGAGGGAGCCTGAGAAATGGC
TACCACA
>HE2_SSU637072
ATGCATGTCCAAGTACAAGCCTCATTAAGGTGAAACCGCGAATGGCTCATTAAATCACACCTAATGCACTGGACGTTGCCAGTTACTTG
GATAACTGTGGTAATTCTAGAGCTAATACACGCATCAAAGCTTCGACCTTGCGGAAGGAGCGCATTTATTAGAACAAAACCAATCGGAC
TTCGGTCCGTCATTTGGTGAATCTGAATAACTTTGCTGATCGCACGGTCCTCGTACCGGCGACGTATCTTTCAAATGTCTGCCTTATCA
ACTTTCGATGGTAGTTTATGCGCCTACCATGGTTGTAACGGGTAACGGAGAATCAGGGTTTGATTCCGGAGAGGGAGCCTGAGAAACGG
CTACCACT
>HE2_SSU637135
ATGCATGTCCAAGTACAAGCCTCATTAAGGTGAAACCGCGAATGGCTCATTAAATCACACCTAATGTCCTGGATAGTGTCAGTTACTTG
GATAACTGCGGTAATTCTGGAGCTAATACACGCAATCAAGCCCCAACCTGACGGACGGGGCGCATTTATTAGAACAAAACCAATTGGCT
TCGGCCATAAATTGGTGAATCTGAATAACTACGCTGATCGCATGGTCTCGTACCGGCGACGTATCCTTCAAGTGTCTGCCTTATCAACT
TTCGATGGTAGTCTATAAGCCTACCATGGTTGTAACGGGTAACGGAGAATAAGGGTTCGACTCCGGAGAGGGAGCCTGAGAAACGGCTA
CCACT
>HE2_SSU644966
ATGCATGTCTAAACAAAAGCCCCAATATGGTGAAGCCGCGAACAGCTCATTACAACAGCCATAGTTTATTGGATCTTATCTTATACTTG
GATACCTGTACTAATTGTAGAGCTAATACATGCAAAAAAGCCACACTCTCTGGGTGTGGTGCATTTATTAGAACAAAACCAATCGGCTT
CGGCCGTTGTTTGGTGAATCTGAATAACTCAGCTGATCGCACAGTCTAGCACTGGCGATGTATCTTTCAAGTGTCTGCCTTATCAACTT
TCGATGGTAGTTTACATGACTACCATGGTTGTAACGGGTAACGGAGAATAAGGGTTCGACTCCGGAGAGGGAGCCTGAGAAATGGCTAC
CACA
>HE2_SSU654005
ATGCATTGTCTAAGCAGAAGCCGATTAATGGTAAAGCCGCGAATGGCTCATTACAACAGCTATTGTTTATTGGACACTATCATCCTACT
TGGATAACTGTGGCAATTCTAGAGCTAATACACGCAGCAAAACGGGGACCTTACGGAACCTGTGCATTTATTAGAACAAAACCAATCGG
GTTTTACCCGTCGTTTGGTGAATCTAGGTAACTCTGCTAATCGCATGGTCTAAGAACCGGCGATATATCTTTCAAATGTCTGCCTTATC
AACTTTCGATGGTAGTTTATGCGCCTACCATGGTTGTAACGGGTAACGGAGAATCAGGGTTCGACTCCGGAGAGGGAGCCTGAGAAACG
GCTACCACA
```

>HE2_SSU655107
ATGCATGTCTAAGCACAAACTTTATGAGTGAAGCCGCGAAAAGCTCATTACAACAGCCGTCGTTTCTTGGATCTCCGAATTTACTTGGA
TAACTGTGGTAATTCTAGAGCTAATACATGCAATCGAGCCCTGAACGTAAGTGATGGGCGCACTTATTAGTAACAAAACCGATCGGTGT
TATGCACCGTACGTTTGGTGAATCTGAATAACTGAGCAGATCGCTTTGGTCTTTGCACCGGCGACGTATCTTTCAAATGTCTGCCCTAT
CAACTTTCGATGGTACGTGATATGCCTACCATGGTTGTAACGGGTAACGGGGAATCAGGGTTCGATTCCGGAGAGGGAGCATGAGAAAC
GGCTACCACA
>HE2_SSU659506
ATGCATGTCTAAGCATAAGCCGAATATGGTAAAGCCGCGAATGGCTCATTACAACAGCCATAGTTTATTGGATCTGAATATCCTACTTG
GATACCTGTGGTAATTCTAGAGCTAATACACGCAAAAAAGCCCTGACTTTACGGAAAGGGCGCATTTATTATAACAAGACCAATTGGCT
TCGGCCATCCATTGGTGACTCTGAATAACTACGCAGATCGCACGGTCTCGTACCGGCGACATATCCTTCAAGTGTCTGCCTTATCAACT
GTCGATGGTAGTTTACATGACTACCATGGTTGTAACGGGTAACGGAGAATTAGGGTTCGACTCCGGAGAGGGAGCCTGAGAAACGGCTA
CCACA
>HE3_SSU110275
ATGCATGTCTAAGTACATACTGATAAATAGTGAAGCTGTGAATGGCTCATTACAACAGCCGTAGTTTATTTGATTTATAGAGTTACATG
GATACCTGTGGTAACCTAAGAGCTAATACGCGCAATTAAGTCCAGACCTTAGGGGACGGACGCGGTTATTAGACCAAAACCAATCGGGC
TTGTCCCGGTATTTTGGTGAATCTGAATAACTCTGCAGATCGCACGGTCCTCGCACCGGCGACATGTCATTCAAATGTCTGCCTTATCA
ACTTTCGATGGTAGTTTATGCGCCTACCATGGTTGTAACGGGTAACGGAGAATTAGGGTTCGACTCCGGAGAGGGAGCCTGAGAAACGG
CTACCACA
>HE3_SSU117415
ATGCATGTCTAAGCATAAGCCGATTAATGGTGAAGCCGCGAATAGCTCATTACAACAGCCATAGTTTATTGGATCTTCTCTCATACTTG
GATACCTGTGGTAATTCTAGAGCTAATACACGCAACAAAACCCTGACTTCGGAAGGGGTGCATTTATTAGAACAAAACCAATTGGCTTC
GGCCATTCATTGGTGACTCTGGATAACTTTGGGCTGATCGCACGGTCTAGCACCGGCGACGTATCTTTCAAATGTCTGCCCTATCAAAT
GTCGAAGGGAGGTGATATGCCTCCCTTGTTTTTAACGGGTAACGGGGAATCAGGGTTCGATTCCGGAGAGGGAGCATGAGAAACGGCTA
CCACA
>HE3_SSU118424
ATGCATGTGTAAGCATGAATCACTTAATGGTGAAGCCGCGAATGGCTCATTATAACAGCCATAGTTTATTAGATTTTTTTTTACTTGGA
TAACTGTGGTAATTCTAGAGCTAATACACGCAGCAAAGCTTCGACCTTACGGAAGGAGCGCATTTATTAGATCAAAACCAATCAGCTCC
GGCTGTATCCTTGACGAATCTGAATAACTTTCCGCTGATCGCATGGCTTCACGCCGGCGACGTATCTTTCTAAGGTGTGCCCTATCAAC
TGTCGACTGTGGCATAGACGCCCACAGTGGTTTTGACGGGTAACGGGGAATCAGGGTTCGATTCCGGAGAGGGAGCATGAGAAACGGCT
ACCACA
>HE3_SSU124287
ATGCATGTCTCAGTACATACTGATTAATAGTGAAACCGCAAATGGCTCATTACAACAGCTATAGTTTATTAGATCTTACTCTACATGGA
TACCTGTGGTAACCTAAGAGCTAATACATGCAACAAAGCCCTATTGCAGGGCGCATTTATTAGGACAAAACCAATCGGGCTTGCCCGCT
TATTGGTGGATCTGAATAACTTTGCTAATCGCACAGTCCTCGTACTGGCGATGTATCTTTCAAATATCTGCCTTATCAACTGTCGATGG
TAGCTTACACGACTACCATGGTTGTTACGGGTAACGGAGAATTAGGGTTCGACTCCGGAGAGGGAGCCTGAGAAACGGCTACCACA
>HE3_SSU124998
ATGCATGTCCAAGTACAAGCCTCATTAAGGTGAAACCGCGAATGGCTCATTAAATCACACCTAATGCACTGGACGTTGCCAGTTACTTG
GATAACTGTGGTAATTCTAGAGCTAATACATGCTGCAAGCTCTGACAAGTTCTTGGACTTGAAGGAGTGCATTTATTAGTACAAAACCA
ATCGGGCTTCTGCCTGCAAATTGGTGAATCTGAATAACTCAGCTGATCGCACAGTCTTGAACTGGCGACGTATCTTTCAAGTGTCTGCC
GTATCAACTGTTGATGGTAGTTTATGTGACTACCATGGTTGTAACGGGTAACGGAGAATAAGGGTTCGACTCCGGAGAGGGAGCCTGAG
AAACGGCTACCACA
>HE4_SSU913283
ATGCATGTCTAAGCACAAACCGAATATGGTAAAGCCGCGCATTGCTCATTACAACAGCCATTGTTTACTGGATCTTGAAAAGTTACTTG
GATAACTGTGGTAATTCTAGAGCTAATACACGCACAAAACTCTAGCCGTCTGGCAAGAGTGCATTTATTAGAACAAAACCAATCAGGCT
TCGGTCTGCTGTTTGGTGAATCTGAATAACTGAGCTGATCGTACCGGTCTTCGCACCGGCGACGTATCTTTCAAGTGTCTGCCTTATCA
ACTTTCGATGGTAGCTTATGTGGCTACCATGGTTGTAACGGGTAACGGAGAATCAGGGTTCGACTCCGGAGAGGGAGCCTGAGAAATGG
CTACCACA
>HE5_SSU181724
ATGCATGTCCAAGTACAAGCCTCATTAAGGTGAAACCGCGAATGGCTCATTAAATCACACCTAATGCACTGGACGTTGCCAGTTACTTG
GATAACTGCGGTAATTCTAGAGCTAATACACGCAATTCAGCCCTGACCTCACGGGAGGGGAGCATTTATTAGAACAAAACCAATTGGCC
TCGGCCATCTGTTGGTGAATCTGAATAACTACGCAGATCGCATGGTCTCGTACCGGCGACGTATCCTTCAAGGGTCTGCCTTATCAACT
TTCGATGGTAGTGTATTTGCCTACCATGGTTGTAACGGGTAACGGAGAATTAGGGTTTGACTCCGGAGAGGGAGCCTGAGAAACGGCTA
CCACA
>HE5_SSU188855
ATGCATGTCTAAGCACAAGCTATTAATTGTGAAGCCGCGAATGGCTCATTATAACAGCCATTGTTTACAGGATATATTATTACTACATG
GATAACTGTGGTAATTCTACAGCTAATACACGCATCAAAACCCCGACTTCGTGAAGGGGTGCGTTTGTTACTTCAAATCAATCGGACTT
CGGTCCGCACTCAAGTGAGATTGAACAATTCAGCTGATCGAACGGTCTAAGAACCGACGACATATCCTTCAAACGTCTGCCTTATCAAC
TTTCGACGTGTGTCTATGCGACAAACGTGGTCGTGACGGGTAACGGAGAATCAGGGTTTGATTCCGGAGAGGGAGCCTGAGAAACGGCT
ACCACA
>HE6_SSU355777
ATGCATGTCTAAGCATAAGCCAAAAAATGGTGAAGCCGCGAATAGCTCATTACAACAGCCATAGTTTATTAGATCTACCAATCCTACTT
GGATAACTTTAGTAATTCTAGAGCTAATACACGCAATCAAGCTCCAACCTCTGGGCGGAGCGCATTTATTAGAACAAAACCAATCGGGT
CCGCCCGTCATTTGGTGAATCTGAATAACTCAGCCGATCGCATGGTCCTCGTACCGGCGACGTATCTTTCAAGTGTCTGCCTTATCAAC
TTTCGATGGTAGTTTACACGCCTACCATGGTTGTAACGGGTAACGGAGAATAAGGGTTCGACTCCGGAGAGGGAGCCTGAGAAACGGCT
ACCACA
>HE6_SSU358048
ATGCATGTGTAAGTACAGACTGTACAACGGTGAAACTGCGAATGGCTCATTAAATCAGTTATGGTTCCTTAGATATTAAAAATCTACAT
GGATAACTGTGGTAATTCTAGAGCTAATACACGCAATCAAGCCCCAACCTGACGGGCGGGGCGCATTTATTAGAACAAAACCAATTGGC
TTCGGCCATTCATTGGTGAATCTGAATAACTACGCTGATCGCACGGTCTCGCACCGGCGACGTATCCTTCAAGTGTCTGCCTTATCAAC
TTTCGATGGTAGTTTACATGACTACCATGGTTGTAACGGGTAACGGAGAATAAGGGTTCGACTCCGGAGAGGGAGCCTGAGAAACGGCT
ACCACA

```
>HE6_SSU360897
ATGCATGTCTAAGCATGAACCGAATATGGTGAAGCCGCGAATGGCTCATTACAACAGCCGTTGTTTCTTGGAGCTTGATTTACTTGGAT
AACTGTGGTAATTCTAGAGCTAATACATGCAACCAAGCTCTGACCTTTGGAAGGAGCGCGTTTATTAGACCAAGACCAATCAGACTTTG
TCTGGAATCTGGTGACTCTGAATAACTTTGCTGATCACACAGTCCTCTCACTGGTGACATATCTTTCAAGTGTCTGCCCTATCAACTGT
CGACTGTGGCATAGACGCCCACAGTGGTTTTGACGGGTAACGGGGAATCAGGGTTCGATTCCGGAGAGGGAGCATGAGAAACGGCTACC
TCA
>HE6_SSU361449
ATGCATGTGTAAGCACAAGCCTTATATGGTGAAGCCGCGAATGGCTCATTACAACAGCCATAGTTTATTAGATCTTATCCTATTACATG
GATAACTGTGGTAATTCTAGAGCTAATACAAGCATTCAAGCTCAGACCTTACGGAATGAGCGCATTTATTAGAACAAAACCAATCGGAC
TTCGGTCCGCTCTTTGGTGAATCTGAATACCTTAGCAGATCGCACGGTCTTTGAACCGGCGACATATCTTTCAAATGTCTGCCTTATCA
ACTTTCGTTGGTAGTTTATGCGCCTACCATGGTTGTAACGGGTAACGGAGAATAAGGGTTCGACTCCGGAGAGGGAGCCTGAGAAACGG
CTACCACA
>HE6_SSU365256
ATGCATGTCCAAGTACAAGCTCGTCCCGAGCGAAACTGCGGATGGCTCATTAAATCAGTTATGGTTCATTGGATCGAGTACCCCCCGAC
ATGGATAACTGTGGTAATTCTAGAGCTAATACATGCAACCAAGGTCTGACCTTTGGAAGGAGCGCGTTTATTAGACCAAGACCAATCAG
ACTTTGTCTGGAATCTGGTGACTGACTAACTTTGCTGATCACACAGTCCTCGCACTGGTGACATATCTTTCAAGTGTCTGCCCTATCAA
CTTTCGATGGTAGTTTATGTGCCTACCATGGTGGTAACAGGTAACAGAGAATAAGGGTTCGACTCCGGAGAGGGAGCCTGAGAAATGGT
TACCACA
>HE6_SSU368318
ATGCATGTGTAAGTACAGACTGTACAACGGTGAAACTGCGAATGGCTCATTAGATCAGTTATGGTTCCTTAGATCGTACAATCCTACTT
GGATAACTGTGGTAATTCTAGAGCTAATACATGCAACCGAGCTCCGACCTCAGGGAAGGAGCGCATTTATTAGACCAAGACCAATCAGG
CTCTGCCTGTCATCTGGTGACTCTGAATAACTTTGCTGATCACATGGTCCTAGTACCGGTGACATATCTTTCAAGTGTCTGCCCTATCA
ACTTTCGATGGTAGTTTATGTGCCTACCATGGTTGTAACGGGTAACGGAGAATAAGGGTTCGACTCCGGAGAGGGAGCCTGAGAAATGG
CTACCACA
>HE6_SSU370544
ATGCATGTCTAAGCATAAACCGAACTAAAGTGAAGCCGCGAATAGCTCATTACAACAGCCGTTGTTTCTTGGATCTCCGCAATACTTGG
ATAACTGAGGTAATTCTTGAGCTAATACACGCAATCGAGCTCCGACCTTCGGGACGAGCGCATTTATTAGAACAAAACCAATCGGTGCT
TGCACTGTGGTTTGGTGAATCTGAATAACTGAGCAGATCGCTTCGGTCTCGTACCGGCGATGTATCCTTCAAGTGTCTGCCTTATCAAC
TTTCGATGGTAGTTTATGTGCCTACCATGGTTGTAACGGGTAACGGGGAATCAGGGTTCGATTCCTGAGAGGGAGCATGAGAAACGGCT
GCCACA
>HE6_SSU378839
ATGCACGTTTAAATATAGGCCGCTTTAAGGTGAAATCGCGAATAGCTCATTACAACAGCCATTGTTTCTTGGATCTTGCTTTCCTACTT
GGATAACTGTGGTAATTTAGGAGCTAATACATGCAACAAAAAACCGATGCAAGAGGAACACATGTATTAGAGTTAAACCAGTCGGTGAA
TCTGAATAACTCAGCAGAGCACATGGGCTAGTCCTGGTGCCATATCTTTCAACTGTTTGTCTTACCCACTTTCTAAGGTTATTTGTGTG
CCTACCATGGTTGTAACGGGTAACGGAGACTAAGGGTTCGACTCCGGAGAGGGAGCCTGAGAAACGGCTACCACT
>HE6_SSU383414
ATGCATGTCTAAGCAGAAGCCGCACAACGGTAAAGCCGCGAATGGCTCATTACAACAGCCGTCGTTTCTTGGATCTCTAATTTTACTTG
GATAACTGTGGTAATTCTAGAGCTAATACACGCACTAAAGCTCCGACCTTACGGGACGAGCGCATTTATTAGAACAAAACTAATCGCGT
TTCGGCCCGTTCGTTGGTGACTCTGAATAACTAAGCCGATCGCACGGTCTCGTACCGGCGACGTATCTTTCAAGTGTCTGCCCTATCAA
ATGTCGAAGGGAGGTGATATGCCTCCCTTGTTTTTAACGGGTAACGGGGAATCAGGGTTCGATTCCGGAGAGGGAGCATGAGAAACGGC
TACCACA
>HE6_SSU383888
ATGCATGTCTAAGAATAAACCGAAAATGGTAACTCTGTGTACGGCTCATTATATCAGCTCAAATTTATTGGATCATATCATCCTACTTG
GATACCTGTGGTAATTCTAGAGCTAATACACGCAATTCAGCCCTGACCTCACGGGAGGGGAGCATTTATTAGAACAAAACCAATTGGCC
TCGGCCATCTGTTGGTGAATCTGAATAACTACGCAGATCGCATGGTCTCGTACCGGCGACGTATCCTTCAAGGTCTGCCTTATCAACTT
TCGATGGTAGTGTATTTGCCTACCATGGTTGTAACGGGTAACGGAGAATTAGGGTTTGACTCCGGAGAGGGAGCCTGAGAAACGGCTAC
CACA
>HE7_SSU232624
ATGCATGTCTAAGCACAAGCCGATATATGGCAAACCCGCGAATGGCTCATTACAACAGCCACTGTTCACTTGATCTGTACCATATCTAC
TTGGATAACTGTGGTAATTCTAGAGCTAATACACGCACCCATGCTCCGTCCGTGAGGAACAAGCGCATTTATTAGAACAAAACCAATCG
ACTTCGGTCGTTCGTTTGTGACTCTGAATAACTTTGCTGATCGCACAGTCATTGTACTGGCGACGCATCTTTCAAGTGTCTGCCTTATC
AACTTTCGACAGTAGTTTCTGTGCCTACCGTAGTTGCAACGGGTAACGGAGAATAAGGGTTCGACTCCGGAGAGGGAGCCTGAGAAACG
GCTACCACA
>HE7_SSU256492
ATGCATGTCTAAGCATAAACCGAATATGGTAAAGCCGCGAATGGCTCATTACAACAGCCATAGTTTATTGGATCTTACTATCCTACTTA
GATAACTGTGGTAATTCTAGAGCTAATACACGCACTCAAGCCCCAACCTGACGGTAGGGGCGCATTTATTAGAACAAGACCAATTGGCC
TCGGCCATCTATTGGTGAATCTGAATAACTACGCTGATCGCACACTCTCGCAGTGGCGACGTATCCTTCAAGTGTCTGCCTTATCAACT
TTCGATGGTAGTTTATATGACTACCATGGTTGTAACGGGTAACGGAGAATAAGGGTTCGACTCCGGAGAGGGAGCCTGAGAAACGGCTA
CCACA
>HE8_SSU829972
ATGCATGTGTAAGCACAAGCCAATGAATGGTAAAGCTGCGAATGGCTCATTACAACAGCCACTGTTCATTTGATCTTAATCCATTACTT
GGATACCTGTTCTAATTGAAGAGCTAATACATGCAACTAAGTCCCAACCGCAAGGGCGGGATGCACTTATTAGACCAAAACCAATCGGG
CTTGCCCGAGGTTTGGTGACTCTGAATAATTTCGCTGACCGCACGGTCTCGCACCGGCGGCGCATCTTTCAAATGTCTGCCTTATCAAC
TTTCGATGGTAGTTTATGCGCCTACCATGGTTGTAACGGGTAACGGAGAATAAGGGTTCGACTCCGGAGAGGGAGCCTGAGAAACGGCT
ACCACA
>HE8_SSU843570
ATGCATGTCCAAGTACAAGCCTCATTAAGGTGAAACCGCGAATGGCTCATTAAATCACACCTAATGCACTGGACGTTGCCAGTTACTTG
GATACCTGTGGTAATTCTAGAGCTAATACACGCAATCAAGCCCTGACCTTACGGGAAGGGCGCATTTATTAGAACAAGACCAATTGGCT
TCGGCCATTTATTGTTGAATCTGAATAACTACGCAGATCGCACGGTCTCGCACCGGCGACATATCCTTCAAGTGTCTGCCTTATCAACT
GTCGATGGTAGTTTACATGACTACCATGGTTTTAACGGGTAACGGAGAATTAGGGTTCGACTCCGGAGAGGGAGCCTGAGAAACGGCTA
CCACA
```

```
>HE9_SSU305678
ATGCATGTCTAAGCACAAACTTTATGAGTGAAGCCGCGAAAAGCTCATTACAACAGCCGTCGTTTCTTGGATATCCGAATTTACTTGGA
TAACTGTGGTAATTCTAGAGCTAATACATGCAATCGAGCCCTGAACGTAAGTGATGGGCGCATTTATTAGTAACAAAACCGATCGGTGT
TATGCACCGTACGTTTGGTGAATCTGAATAATTGAGCAGATCGCTTTGGTCTTGTACCGGCGACGTATCTTTCAAGTGTCTGTTTTATC
AACTTTAGATGTTAGTTTATATGACTAACATGGTTGTCACGGATAACGGAGAATAAGGGTTCGACTCCGGAGAGGGAGCCTGAGAAACG
GCTACCACT
>HF1_SSU759758
ATGCAAGTGTCAGCTCAAGCCATATTATGGTTAAGCCGCGGAAAGCTCATTACAACAGCCATTGTTCACTTGATCTTGACTATCCTACT
TGGATAACTGTGGTAATTCTAGAGCTAATACGTGCAACAATGCTCAGGTAGTCCTTCGGGGCGACGAGCGCAGTTATTAGAACAAAACC
AATCGGGCTTCGGTCCGTCGGTTTGGTGGATCTGAATAACTACAGCTGATCGCACAGTCTTCGTACTGGCGACGAATCTTTCAAGTGTC
TGCCTTATCAGCTGTCGATGGTAGTCTACGTGGCTACCATGGCTGTAACGGGTAACGGAGAATAAGGGTTCGACTCCGGAGAGGGAGCC
TGAGAAACGGCTACCACA
>HF1_SSU763392
ATGCATGTCTAAGTACAAGCCGAGTTAAGGTGAAACCGCGAATGGCTCATTAAATCACACCTAATATACTGGATAGTGTCAGCTACTTG
GATACCTGTGGTAATTCTAGAGCTAATACACGCACGAAAGCCCTGACTTCGGGAGGGGCGCATTTATTAGAACAAAACCAATCGGGCTT
GCCCGTCATTTGGTGAATCTGAATAACTCAGTTGATCGCACAGTCCTCGCACTGGCGACGTATCTTTCAAGTGTCTGCCTTATCAACTT
TCGATGGTAGTTTACATGACTACCATGGTTGTAACGGGTAACGGAGAATAAGGGTTCGACTCCGGAGAGGGAGCCTGAGAAATGGCTAC
CACA
>HF1_SSU764346
ATGCATGTCTAAGCAAAAGCCTCAAAATGGTGAAGCCGCGAATAGCTCATTACAACAGCCATAGTTTATTGGATCTTCTCTCATACTTG
GATACCTGTGGTAATTCTAGAGCTAATACACGCAAACAAACTCTGAGCTCTGGCGAGGAGTGCATTTATTAGAACAAAACCAATGGACC
CCGGTCCTTGTTTGGTGAATCTGAATAACTCAGTTGATCGCACAGTCTAGCACTGGCGACGTATCTTTCAAGTGTCTGCCTTATCAACT
GTCGATGGTAGTTTATATGACTACCATGGTTGTAACGGGTAACGGAGAATAAGGGTTCGACTCCGGAGAGGGAGCCTGAGAAATGGCTA
CCACA
>HF1_SSU774294
ATGCATGTCTAAGCACACGCCTTAAAATGGTAAAGCCGCGAATGGCTCGGTATAACAGCTACGGTTTATTAGATATTAGTTGTTTACTT
GGATAACTGTGGTAATTCTAGAGCTAATACATGCACTTTAGCTCGGACCTCACGGAAAGAGCGCATTTATTAGATCAAAACCAATCGGG
CCTCGGTCCGTGTTTTGGTGACTCTGAATAACTCAGTTGATCGCACAGTCTTGTACTGGCGACGTATCTTTCAAATGTCTGCCTTATCA
ACTTTCGATGGTAGGTTATACGCCTACCATGGTTATAACGGGTAACGGAGAATCAGGGTTCGACTCCGGAGAGGGAGCCTGAGAAATGG
CTACCACA
>HF1_SSU779114
ATGCATGTCTATGCATAAGCCTAAATAAGGTGAAGTCGCGAATTGCTCATTACAACAGCCATTGTTTACTGGATCTTAATATCCTACTT
GGATAACTGTGGTAATTCTAGAGCTAATACACGCACCAAGCTCTGACCGCAAGGGATGAGCGCATTTATTAGAACAAAACCAATCGGGT
TCGTCCCGTCTTTGGTGGATCTGAATAACTCAGCTGATCGCATGGTCTCGCACCGGCGACGTATCTTCCAAGTGTCTGCCTTATCAACT
TTTGATGGTAGTTTATGCGACTACCATGGTTGTAACGGGTAACGGAGAATAAGGGTTCGACTCCGGAGAGGGAGCCTGAGAAACGGCTA
CCACA
>HF1_SSU780927
ATGCATGTGTAAGTACAAACCTGTACATGGTGAAACTACGAATGGCTCATTAAATCAGTTGTGGTTCCTTAGATCGTTTTACAGTTTGG
ATAACTGTAGTAATTCTAGAGCTAATACACGCAACAAGCTCTGACCTCTCGGGGAAAGAGTGCATTTATTAGAACAAAACCAATCGGGC
TTCGGTCTGTCAATTGGTGAATCTGAATAACTCAGCTGATCGCACGGTCTTGTACCGGTGACGCATCTTTCAAGTGTCTGCCTTATCAA
CTGTTGATGGTAGTTTATGTGACTACCATGGTTGTAACGGGTAACGGAGAATAAGGGTTCGACTCCGGAGAGGGAGCCTGAGAAACGGC
TACCACA
>HF2_SSU192072
ATGCATGTGTAAGAATAAACCGAATATGGTAAATCCGCGAATGGCTCATTATTCAGCCACAAATCATTGGATCTAATCAGTTACTTGGA
TAACTGTTCAAAAGGAAGAGCTAAGACATGCCTCGAAGGCCAAGCGCAAGCTTGGTCGCACTTCTTAGAAAAGACCAATTGGCCTCGGC
CATCCATTGGTGAATCTTCCGAAGAAAGCAGATCGCACGGTCTAGTACCGGCGACATATCCTTCATGTGTCTGCCTTATCAACTGTCGA
TGGTAGTTTATTGGACTACCATGGTTGTAACGGGTAACGGAGAATTAGGGTTCGACTCCGGAGAGGGAGCCTGAGATACGGCTACCACA
>HF2_SSU204352
ATGCATGTCTAAGTACAGGCCTCACTAAGGTGAAACCGCGAATGGCTCATTAAATCACACCTAATATACTGGATAGTATCAGTTACTTG
GATAACTGCGGTAATTCTAGAGCTAATACACGCACCCATGCTCCGTCCGTGAGGAACGAGTGCATTTATTAGAACAAAACCAATCGACT
TCGGTCGTTCGTTTGTGACTCTGAATAACTTTGCTGATCGCACAGTCATTGTACTGGCGACGCATCTTTCAAGTGTCTGCCTTATCAAC
TTTCGACGGTAGTTTCTGTGCCTACCGTGGTTGCAACGGGTAACGGAGAATAAGGGTTCGACTCCGGAGAGGGAGCCTGAGAAACGGCT
ACCACA
>HF2_SSU205129
ATGCATGTCTAAGTACAGGCCTCACTAAGGTGAAACCGCGAATGGCTCATTAAATCACACCTAATATACTGGATAGTATCAGTTACTTG
GATAACTGCGGTAATTCTAGAGCTAATACACGCACTCAAGCCCCAACCTGACGGTAGGGGCGCATTTATTAGAACAAGACCAATTGGCC
TCGGCCATCTATTGGTGAATCTGAATAACTACGCTGATCGCACACTCTCGCAGTGGCGACGTATCCTTCAAGTGTCTGCCTTATCAACT
TTCGATGGTAGTTTATATGACTACCATGGTTGTAACGGGTAACGGAGAATAAGGGTTCGACTCCGGAGAGGGAGCCTGAGAAACGGCTA
CCACA
>HF2_SSU208147
ATGCATGTCTATGCACGAGCCGAAAATGGTGAAGCCGCGAATGGCTCATTACAACAGCCTTGGTTTATTGGATCTATTATATCCACTTG
GATAACTGTGGTAATTCTAGAGCTAATACACGCAACTAAACTCCAACCTTGCGGAAGGAGTGCATTTATTAGAACAAAACCAATCGGCT
TCGGCCGTTACTTGGTGAATCTGAATAACTCAGTTGATCGCACGGTCTTTGTACTGGCGACATATCTTTCAAGTGTCTGCCTTATCAAC
TTTCGATGGTAGTTTAGACGACTACCATGGTTGTTACGGGTAACGGAGAATTAGGGTTCGACTCCGGAGAGGGAGCCTGAGAAATGGCT
ACCACA
>HF2_SSU210357
ATGCATGTCTAAGCACAAACTATTTAATTGTGAAGCCGCGAATGGCTCATTATAACAGCCATTGTTTACTGGATATATTTTTACTACAT
GGATAACTGTGGTAATTCTACAGCTAATACACGCAGCAGAACCCCGACTTAATGAAGGGGTGCGTTTGTTACTTCAAACCAATCAGGCT
TCGGTCTGAATTCAAGTGATATTGAACAATTTAGCTGATCGAACGGTCTATGAACCGACGACATATCCTTCAAACGTCTGCCTTATCAA
CTTTCGATGGTAGCCTACACGTCTACCATGGTTGTAACGGGTAACGGAGAATCAGGGTTTGATTCCGGAGAGGGAGCCTGAGAAATGGC
TACCACA
```

>HF3_SSU989895
ATGCAAGTGTCAGCTCAAGCCTATGTATGGTTAAGCCGCGAATGGCTCATTACAACAGCCACTGTTTACTTGATCTTGATAATCCTACT
TGGATAACTGTGGTAATTCTAGAGCTAATACATGCAACTATGCTCCGACCTTACGGGACGAGCGCAACTATTAGAACCAAACCAATCGG
GTTTCGGCCCGTTCGGTGGTGAATCTGAATAACTGTTTGCTGATCGCACGGTCTTTGCACCGGCGACGCATCTTTCAAGTGTCTGCCTT
ATCAACTTTCGATGGTAGTTTATGTGCCTACCATGGTTGTAACGGGTAACGGAGAATAAGGGTTCGACTCCGGAGAGGGAGCCTGAGAA
ACGGCTACCACA
>HF3_SSU990962
ATGCATGTCTATGCATAAACCGAATATGGTAAAGCCGCGCATGGCTCATTACAACAGCCATAGTTTATAGGATCTTACTATCCTACTTT
GATAACTGTGGTAATTCTAGAGCTAATACACGCACTCAAGCCCCAACCTGACGGTAGGGGCGCATTTATTAGAACAAGACCAATTGGCT
TCGGCCATCTATTGGTGAATCTGAATAACTACGCTGATCGCACACTCTCGAAGTGGCGACGTATCCTTCTAAGGTGTGCCCTATCAACT
GTCGACTGTGGCATAGACGCCCACAGTGGTTTTGACGGGTAACGGGGAATCAGGGTTCGATTCCGGAGAGGGAGCCAGAGAAACGGCTA
CCACA
>HF4_SSU606153
ATGCATGTCTAAGCATAAGCCGAATATGGTAAAGCCGCGAATGGCTCATTACAACAGCCATAGTTTATTGGATCTTAGAGTCCTACTTG
GATACCTGTGGTAATTCTAGAGCTAATACACGCATTCAAGCCCCAACCTGACGGGCGGGGCGCATTTATTAGAACAAAACCAATTGGCT
TCGGCCATTCATTGGTGAATCTGAATAACTACGCTGATCGCACGGTCTCGCACCGGCGACGTATCCTTCAAGTGTCTGCCTTATCAACT
TTCGATGGTAGTTTACATGACTACCATGGTTGTAACGGGTAACGGAGAATAAGGGTTCGACTCCGGAGAGGGAGCCTGAGAAACGGCTA
CCACA
>HF4_SSU614317
ATGCATGTCTAAGTACATACCTTCACACGGTGAAACTGCGAATGGCTCATTAAATCAGTTATGGTTCCTTAGATCGATACACTCCTACT
TGGATAACTGTGGCAATTCTAGAGCTAATACACGCACTAAAGCTCCGACCTTACGGGACGAGCGCATTTATTAGAACAAAACCAATCGG
GTTTCGGCCCGTTCGTTGGTGACTCTGAATAACTAAGCCGATCGCACGGTCTCGTACCGGCGACGTATCTTTCAAGTGTCTGCCTTATC
AACTTTCGATGGTAGTTTATGTGCCTACCATGGTTGTAACGGGTAACGGAGAATAAGGGTTCGACTCCGGAGAGGGAGCCTGAGAAACG
GCTACCACA
>HF4_SSU619471
ATGCACGTTCTAATATGAGCATTAAAAATGTGAAATCGCGAATAGCTCATTACAACAGCCATTGTTTCTTGGATCTTATATTCCTACTT
GGATAACTGTGGTAATTCTAGAGCTAATACATGCAATGAAGTTCCAACGCAAGAGGAATGCATTTATTAGAGCTAAACCAATCAGGGGC
AACCATGTTTGTTTGGTGAATCTGAATAACTTAGCAGAGCACATGGGCTAGTCCTGGTGCCATATCTTTCAAGTGTCTGCCTTATCAAC
TTTCGATGGTAGTTTATGTGCCTACCATGGTTGTAACGGGTAACGGAGAATAAGGGTTCGACTCCGGAGAGGGAGCCTGAGAAACGGCT
ACCACA
>HF4_SSU620879
ATGCATGTGTAAGTACAGACTGTACAACGGTGAAACTGCGAATGGCTCATTAGATCAGTTATGGTTCCTTAGATCGTACAATCCTACTT
GGATAACTGTGGCAATTCTAGAGCTAATACACGCACTCAATCCCTGACTTCGGAAAGGGAGCATTTATTAGAACAAGACCAATTGGCTT
CGGCCATCTATTGGTGAATCTGAATAACTACGCAGATCGCACAGGCTCGTCCTGGCGACATATCCTTCAAGTGTCTGCCTTATCAACTG
TCGATGGTAGTTTATTGGACTACCATGGTTGTAACGGGTAACGGAGAATTAGGGTTCGACTCCGGAGAGGGAGCCTGAGAAACGGCTAC
CACA
>HF4_SSU622464
ATGCATGTCTATGCACAAGCCGAAAATGGTGAAGCCGCGAATGGCTCATTACAACAGCCACTGTTTACTTGATCTTGATTATCCTACTT
GGATAACTGTGGTAATTCTAGAGCTAATACATGCCAAGATGCTCCGACCTTACGGGACGAGCGCACTTATTAGACCAAGACCAATCGGG
CTTCGGCTCGTAGTCTGGTGACTCTGAATAACTCTGCCGATCGCACGGTCTTTGTACCGGCGACGCATCTTTCAAGTGTCTGCCTTATC
AACTTTCGATGGTAGTTTCTGTGCCTACCATGGTTGTAACGGGTAACGGAGAATAAGGGTTCGACTCCGGAGAGGGAGCCTGAGAAACG
GCTACCACA
>HF4_SSU624085
ATGCATGTCTAAGTACAAGCCTCATTAAGGTGAAACCGCGAATGGCTCATTAAATCACACCTAATATACTGGATAGTATCAGTTACTTG
GATAACTGCGGTAATTCTGGAGCTAATACATGCGTTCAAGCCCCAAACTTGCGTGCGGGGCGCTTTTATTAGACCAAGACCAATCAGGC
ATTGCCTGGAATCTGGTGACTCTGAATAACTTTGCTGATCACATGGTCCTAGCACCGGTGACATATCTTTCAAGTGTCTGCCCTATCAA
CTTTCGATGGTAGTTTATGTGCCTACCATGGTGGTAACGGGTAACGGAGAATAAGGGTTCGACTCCGGAGAGGGAGCCTGAGAAATGGC
TACCACA
>HF4_SSU625424
ATGCATGTCTATGCAGAAGCCGAACTATCGCAAAGCTGCAAATGGCTCATTACAACAACTTTTGTTTCTTGGATCTCTCTGATCTACTT
GGATACCTGTGGTAATTCTAGAGCTAATACACGCACCAAATCTCTGACCTTTGGGGATGAGTGCATTTATTAGAACAAAATCAATCGGG
CTCTGCTCGTATGATGGTGACTCTGAATAACTACGCTGATCGCATGGTCTCATACTGGCGACGTATCTTCCAAGTATCTGCCTTATCAA
CTGTTGATGGTAGTTTATTTGCCTACCATGGTTGTAATGGGTAATGGAGAATAAGGGTTCAACTCCAGAGAGGGAGCCCGGGAAACGGC
TACCACA
>HF4_SSU628562
ATGCATGTCTAAGCATGAGCCGTACTATGGTGAAGCCGCGAACAGCTCATTACAATAGCCGTTGTTTCTTGGATCTCCAAACACTACAT
GGATAACTGTGGTAATTCTAGAGCTAATACATGCGTACATGCGTCAACTGGTGCAAGCCGGGAGACGTGCATTTATTGGATCAGAACCA
TCCGGCCTTCGGGCCGTACTCTGGTGAATCTAAATAACTGAGCGGAGCACACGCTCTTGCAGCGGTGCCAGTTCGTTCAAGTGTCTACC
CTATCAACTTGCGATGGTAAATTACAAGCTTACCATGGTGGTAACGGGTAACGGAGAATCAGGGTTCGATACCGGAGAGGGAGCCTGAG
AAATTGCTACCACA
>HF4_SSU631524
ATGCATGTCTAAGTACACTTCCTTGTATGGAGAAACTGCGAATGGCTCATTACAACAGCCACTGTTCACTTGATCTGTATTATCCTACT
TGGATAACTGTGGTAATTCTAGAGCTAATACACGCACCCATGCTCCGACCTGAGGGGACGAGCGCATTTATTAGAACAAAACCAATCGG
GCCTCGGCCTGTTTCGTTTGTGACTCTGAATAACTCTGCTGATCGTACGGTCCCGTACCGACGACGCATCTTTCAAGTGTCTGCCTTAT
CAACTTTCGATGGTAAGTTCTGTGCTTACCATGGTTGTAACGGGTAACGGAGAATAAGGGTTCGACTCCGGAGAGGGAGCCTGAGAAAC
GGCTACCACA
>HF4_SSU632264
ATGCATGTCTATGCACAAGCCGATTCGGCGAAGCCGCGAATGGCTCATTACAACAGCTGTGGTTTCTTGGATCTTTCAATCCTACTTGG
ATAACTGTGGCAATTCTAGAGCTAATACACGCACTGAAACCGCGGTCCTTGGGCTGCGGTGCATTTATTAGAACAAAGCCAACCGGGCC
TTGGCCTGACTGCTTGGCGAATCTGAATAACCTGGCTGATCGCACGGTCTCGCACCGGCGACGCATCTTTCAAGTGTCTGCCTTATCAA
CTGTCGATGGTAGGTTACGTGCCTACCATGGTTGTAACGGGTAACGGAGAATAAGGGTTCGACTCCGGAGAGGGAGCCTGAGAAATGGC

TACCACA
>HF4_SSU635045
ATGCATGTGTAAGAATAAACCGAATATGGTAAATCCGCGAATGGCTCATTATTCAGCCTCAATTTATTGGATCTAATCAGTTACTTGGA
TAACTGTTCAAAATGAAGAGCTAAGACATGCCTCGAAAATCCAGCGCAAGCCGGATTGCACTTCTTAGAAAAGACCAATTGGCTTCGGC
CATCCATTGGTGAATCTTCTGAAATTCGCAGATCGCACGGTCTAGTACCGGCGACATACCCTTCAAATGTCTTCCTTATCAACTGTCGA
TGGTAGTTTATTGGACTACCATGGTTGTAACGGGTAACGGAGAATTAGGGTTCGACTCCGGAGAGGGAGCCTGAGATACGGCTACCACA
>HF5_SSU991188
ATGCATGTCTAAGCACAACTATTTTATTTGTGAAGCCGCGAATGGCTCATTACAACAGCCATAGTTCACTGGATATATTCCTTTTACAT
GGATAACTGAGGTAATTCTTCAGCTAATACACGCTTCAAAACCCCGACTTTTTGGAGGGGTGCGTTTGTTACTTCAAATCAATCGGGTT
TCGGCCCGTTTATAAGTGATATTGAACAATTTAGCTGATCGCACGGTCTGAGCACCGGCGACATATCCTTCAAATGTCTGCCTTATCAA
CTTTCGATGGTAGATTATGCGCCTACCATGGTTGTTACGGGTAACGGAGAATCAGGGTTTGATTCCGGAGAGGGCGCCTGAGAGACGGC
GGCCACA
>HF5_SSU995414
ATGCATGTCTAAGTACAAGCCGAGTTAAGGTGAAACCGCGAATGGCTCATTAAATCACACCTAATATACTGGATAGTGTCAGTTACTTG
GATAACTGTGGTAATTCTAGAGCTAATACAAGCATTCAAGCTCAGACCTTACGGAATGAGCGCATTTATTAGAACAAAACCAATCGGAC
TTCGGTCCGCTCTTTGGTGAATCTGAATACCTTAGCAGATCGCACGGTCTTTGAACCGGCGACATATCTTTCAAATGTCTGCCTTATCA
ACTTTCGTTGGTAGTTTATGCGCCTACCATGGTTGTAACGGGTAACGGAGAATAAGGGTTCGACTCCGGAGAGGGAGCCTGAGAAACGG
CTACCACA
>HF6_SSU329881
ATGCATGTCTAAGCATGAACCGAATATGGTGAAGCCGCGAATGGCTCATTACAACAGCCGTTGTTTCTTGGATCTTGATTTACTTGGAT
AACTGTGGTAATTCTAGAGCTAATACATGCAACCAAGCTCTGACCTTGGAAGGAGCGCGTTTATTAGACCAAGACCAATCAGACTTTGT
CTGGAATCTGGTGACTCTGAATAACTTTGCTGATCACACAGTCCTCGCACTGGTGACATATCTTTCAAGTGTCTGCCCTATCAACTTTC
GATGGTAGTTTATGTGCCTACCATGGTGGTAACGGGTAACGGAGAATAAGGGTTCGACTCCGGAGAGGGAGCCTGAGAAATGGCTACCA
CA
>HF6_SSU338435
ATGCATGTCTACGTACAGAGATTTTTCTCGAAACCGCGAACGGCTCATTACAACAGCCATAGTTTATTGGATCTACAAATCCTACTTGG
ATACCTGTGGTAATTCTAGAGCTAATACACGCAACAGATCCCTGACCTTGCGGAAGGGGAGCATTTATTAGAACAAAACCAATTGGCTT
CGGCCATTAGTTGGTGAATCTGAATAACTACGCAGAGCATACGAGCTCGTCTCGATGCCATATCCTTCAAGTGTCTGCCCTATCAACTG
TCGATGGTAGGTGATATGCCTACCATGGTTGCAACGGGTAACGGGGAATCAGGGTTCGATTCCGGAGAGGGAGCCTGAGAAACGGCTAC
CACG
>HF6_SSU338739
ATGCATGTCTAAGCACAAGCTGATTTAAAGTGAAGCCGCGAATAGCTCATTACAACAGTCATAGTTTACTTGATTTTGTTTTACTTGGA
TAACTGTGGTAATTCTAGAGCTAATACATGCTGCAAGCTCCAACAAGTTCTTTGGACTTGAAGGAGTGCATTTATTAGAACAAAACCAA
TCGGGTCTTTGACCTGTCCATTGGTGAATCTGAATAACTCAGCTGATCGCACAGTCCTGAACTGGCGACGTATCTTTCAAGTGTCTGCC
TTATCAACTGTTGATGGTAGTTTATGTGACTACCATGGTTGTAACGGGTAACGGAGAATAAGGGTTCGACTCCGGAGAGGGAGCCTGAG
AAACGGCTACCACA
>HF7_SSU385021
ATGCATGTCTAAGAATAGGGATTATTCCCAAATCCGCGAATGGCTCATTACAACAGCCTTAGTTTATTGGATCTACAAATCCTACATGG
ATACCTGTGGTAATTCTAGAGCTAATACACGCAAGAAAGCCCCGACCTTACGGGAGGGGTGCATTTATTAGAACAAGACCAATTGGCTT
CGGCCATCCATTGGTGAATCTGAATAACCTAGCAGAGCATACGAGCTCGTCTCGATGCCATATCCTTCAAGTGTCTGCCCTATCAACTG
TTGACGGTAGGTTACATGCCTACCGTGGTTGTAACGGGTAACGGAGAATTAGGGTTCGACTCCGGAGAGGGAGCCTGAGAAACGGCTAC
CACA
>HF7_SSU390110
ATGCATGTCTAAGCACAAGCTTAAACAAAGTGAAGCCGCGAATAGCTCATTACAACAGCCATTGTTTACTTGATCTTGAAATCCTACTT
GGATAACTGTGGTAATTCTAGAGCTAATACACGCAACAAGCTCTGACCCCTCGGGGAAAGAGTGCATTTATTAGAACAAAACCAATCGG
ACTTCGGTCTGTCAATTGGTGAATCTGAATAACTCAGCTGATCGCACGGTCTTGTACCGGTGACGCATCTTTCAAGTGTCTGCCTTATC
AACTGTTGATGGTAGTTTATGTGACTACCATGGTTGTAACGGGTAACGGAGAATAAGGGTTCGACTCCGGAGAGGGAGCCTGAGAAACG
GCTACCACA
>HF7_SSU398053
ATGCATGTCTAAGTACAAGCCGAGTTAAGGTGAAACCGCGAATGGCTCATTAAATCACACCTAATATACTGGATAGTGTCAGTTACTTG
GATAACTGCGGTAATTCTAGAGCTAATACATGCAACAATGCTCAGGTAGCCCTTCGGGGTGACGAGCGCAATTATTAGAACAAAACCAA
TCGGGCTTCGGTCCGTTCGGTTTGGTGGATCTGAATAACTATAGCTGATCGCACAGTCTTTGCACTGGCGACGCATCTTTCAAGTGTCT
GTCCTATCAGCTGTCGATGGTATTCTATACGATTACCATGGCTGTAACGGGTAACGGAGAATAAGGGTTCGACTCCGGAGAGGGAGCCT
GAGAAACGGCTACCACA
>HF7_SSU407024
ATGCATGTCTAAGCAAAATGGTGAAGCCGCGAATAGCTCATTACAACAGCCATAGTTTATTGGATCTTGACTTCCTACTTGGAATACCT
GTGGTAATTCTAGAGCTAATACATGCAAATAAGCTCCAACCTCTGGGCGGAGCGCATTTATTAGAACAAAACCAATCGGCTTCGGCCGT
AGTTTGGTGAATCTGAATAACTCAGCTGATCGCATGGTCCTCGCACTGGCGACGTATCTTTCAAGTGTCTGCCTTATCAACTTTCGATG
GTAGTTTACACGCCTACCATGGTTGTAACGGGTAACGGAGAATAAGGGTTCGACTCCGGAGAGGGAGCCTGAGAAACGGCTACCACA
>HF7_SSU407761
ATGCATGTCTAAGCACAAGCTTAAATAAAGTGAAGCCGCGAATAGCTCATTACAACAGCCATTGTTCACTTGATCTTGAAATCCTACTT
GGATAACTGTGGTAATTCTAGAGCTAATACACGCAAAAAGCTCTGACCTTACAGGGAAGAGTGCATTTATTAGAACAAAACCAATCGGG
CTTCGGCCTGTCAATTGGTGAATCTGAATAACTCAGCCGATTGCACGGTCTTGAACCGGCAACATATCTTTCAAGTGTCTGCCTTATCA
ACTGTTGATGGTAGTTTTTGTGACTACCATGGTTGTAACGGGTAACGGAGAATAAGGGTTCGACTCCGGAGAGGCAGCCTGAGAAACGG
CTACCACA
>HF7_SSU409331
ATGCATGTCTAAGTACAAGCCCCATTAAGGTGAAACCGCGAATGGCTCATTAAATCACACCTAATATAATGGATAGTGTCCGTTACTTT
GATAACTGTGGTAATTCTAGAGCTAATACACGCACATAAGCTCTAACCGTAAGGAAAGAGCGCATTTATTAGAACAAAAGCATCCGGCA
TTTAGCCGTTACTTGGTGAATCTGAATAACTCTGCTAATCGCACAGTCAGAGTACTGGAGATGCATCTTTCAAGTGTCTGCCTTATCAA
CTTTCGATGGTAGTTTATATGCCTACCATGGTTATAACGGGTAACGGAGAATAAGGGTTCGACTCCGGAGAGGGAGCCTGAGAAATGGC
TACCACA

>HF8_SSU795426
ATGCATGTCTAAGCAGAAGCCGAAAATGGTGAAGCCGCGAATAGCTCATTACAACAGCCTTAGTTTCTTGGATCTTCAGTTCCTACTTG
GATAACTGTGGTAATTCTAGAGCTAATACACGCTAACAAGTCCCGGCCTCTGGCTGGGCTGCATTTATTAGACCAAAACCAATCGGACT
CTGGTCCGTGTATGGTGAATCTGAATAACTTTGCTGATCGCACGGTCCTCGTACCGGCGACGCGTCTTTCAAGTGTCTGGCTTATCAAC
TTTCGATGGTAGTTTATACGACTACCATGGTTGTAACGGGTAACGGAGAATAAGGGTTCGACTCCGGAGAGGGAGCCCGAGAAATGGCT
ACCACA
>HF9_SSU14048
ATGCATGTCCAAGTACAAGCCTCATTAAGGTGAAACCGCGAATGGCTCATTAAATCACACCTAATGCACTGGACGTTGCCAGTTACTTG
GATAACTGCGGTAATTCTGGAGCTAATACACGCATACAAGTTCCGACGTAAGAAGGAATGCATTTATTAGAACTAAACCAATCGGGCTT
GCCCGTTGTTTGGTGAATCTGAATAACTCCGCAGATCGCATGGTCTAGCACCGGCGACATATCTTTCAAGTGTCTGCCTTATCAAATGT
CGAAGGGACGTGATATGCCTCCCTTGTTTGTAACGGGTAACGGGGAATCAGGGTTCGATTCCGGAGAGGGAGCATGAGAAACGGCTACC
ACA
>HF9_SSU14296
ATGCACGTGCAAGCACAAGCCAAATTATGGTGAAGCCGCGAATGGCTCATTACAACAGCTATTGTTTATTGGATTTGATGTTCACTTGG
ATAACTGTGGTAATTCTAGAGCTAATACATGAAACCTAGCTTTTGCCGCAAGGTTAGAGCGCAATTATTAGACTAAAACCAACCAGCTT
TAGCTGATACCTGGTGAATCTGAATAAACCTGCTTATTGCGTAATCAAAGCACTGGCGAAGCATCTTTCAAGTGTCTGCCTTATCAACT
GTTGAAGGTAGTTTACATGCCTACCATGGTTATAACGGGTAACGGAGAATAAGGGTTTTACTCCGGAGAGGGAGCCTGAGAAATGGCTA
CCACA
>HF9_SSU17250
ATGCATGTCTAAGTACAGGCCTCACTAAGGTGAAACCGCGAATGGCTCATTAAATCACACCTAATATACTGGATAGTATCAGTTACTTG
GATAACTGCGGTAATTCTGGAGCTAATACATGCAACAAAGCCCTATTGCAGGGCGCATTTATTAGGACAAAACCAATCGGGCTTGCCCG
CTTATTGGTGGATCTGAATAACTTTGCTAATCGCACAGTCCTCGTACTGGCGATGTATCTTTCAAATATCTGCCTTATCAACTGTCGAT
GGTAGCTTACACGACTACCATGGTTGTTACGGGTAACGGAGAATTAGGGTTCGACTCCGGAGAGGGAGCCTGAGAAACGGCTACCACA
>HF9_SSU17844
ATGCACGTCTAAGCACAAGCCAAATTGAAGGTAAAGCCGCGAATGGCTCATTACAACAGCCATTGTTTATTGGATCTGTTTAACTTGGA
TAACTGTGGTAATTCTAGAGCTAATACACGCACATAAGCTCTAACCGTAAGGAAAGAGCGCATTTATTAGAACAAAACCATCCGGCATT
TAGCCGTTACTTGGTGAATCTGAATAACTCTGCTAATCGCACAGTCAGAGTACTGGCGATGCATCTTTCAAGTGTCTGCCTTATCAACT
TTCGATGGTAGTTTATATGCCTACCATGGTTATAACGGGTAACGGAGAATAAGGGTTCGACTCCGGAGAGGGAGCCTGAGAAATGGCTA
CCACA
>HF9_SSU18227
ATGCATGTCTAAGTACAAGCCTCATTAAGGTGAAACCGCGAATGGCTCATTAAATCACACCTAATGTACTGGACAGTCTCAGTTACTTG
GATACCTGTGGTAATTCTAGAGCTAATACACGCAAAAAAGCCCTGACTTTACGGAAAGGGCGCATTTATTAGAACAAGACCAATTGGCT
TCGGCCATCCATTGGTGACTCTGAATAACTACGCAGATCGCACGGTCTCGTACCGGCGACATATCCTTCAAGTGTCTGCCTTATCAACT
TTCGATGGTAGTTTACATGACTACCATGGTTGTAACGGGTAACGGAGAATAAGGGTTCGATTCCGGAGAGGGAGCCTGAGAAACGGCTA
CCACA
>HF9_SSU19963
ATGCATGTCTAAGCACAAGCCTAATACGGTGAAGCCGCGAATGGCTCATTACAACAGCCTTTGTCTATTTGATGTTGAAAATCTACTTG
GATAACTGTGGAAAAGCCAGAGCTAATACATGCTTACAAACTCCGACCTTGCGGAAGGGGTGCATTTATTAGTGCAAAACCAATCGGGC
GCTTCGCGTCCCGTCGTATGGTGAATCTGAATAACTAAGCCGATCGTACGGTCCTCGCACCGGCGACGAATCATTCGAGGTTCTGCCAT
ATCAACTTTGACGGTAGTTTACGTGACTACCATGGTTATAACGGGTAACGGAGAATAAGGGTTCGACTCCGGAGAAGCAGCCTGAGAAA
CGGCTACTACA
>HF9_SSU20251
ATGCACGTTTAAATACAAGCCTTAAAATGGTGAAATCGCGAATAGCTCATTACAACAGCCATTGTTTCTTGGATCTTACTTTCTACTTG
GATAACTGTGGTAATTCTAGAGCTAATACACGCATACAAGTTCCGACGCAAGAAGGAATGCATTTATTAGAACTAAACCAATCGGGCTT
GCCCGTTGTTTGGTGAATCTGAATAACTCCGCAGATCGCATGGTCTAGCACCGGCGACATATCTTTCAAGTGTCTGCCTTATCAACTTT
CGATGGTAGTTTATGTGACTACCATGGTTGTAACGGGTAACGGAGAATTAGGGTTCGACTCCGGAGAGGGAGCCTGAGAAACGGCTACC
ACA
>HF9_SSU22538
ATGCATGTGTAAGTACAGACTGTACAACGGTGAAACTGCGAATGGCTCATTAGATCAGTTATGGTTCCTTAGATCGTACAATCCTACTT
GGATAACTGTGGTAATTCTAGAGCTAATACATGCACTTTAGCTCGGACCTCACGGAAAGAGCGCATTTATTAGATCAAAACCAATCGGG
CCTCGGTCCGTGTTTTGGTGACTCTGAATAACTCAGTTGATCGCACAGTCTTGTACTGGCGACGTATCTTTCAAATGTCTGCCTTATCA
ACTTTCGATGGTAGGTTATACGCCTACCATGGTTATAACGGGTAACGGAGAATCAGGGTTCGACTCCGGAGAGGGAGCCTGAGAAATGG
CTACCACA
>TF1_SSU676746
ATGCATGTCTAAGCACAAGCCAAAAATGGTGAAGCCGCGAATGGCTCATTACAACAGCCGTAGTTTCTTGGATCTTTCTAATCTACTTG
GATAACTGTGGTAATTCTAGAGCTAATACACGCACAAAAGCTCTATCCGAATGGAAGAGCGCATTTATTAGAACTAAACCGACCGGGTG
TTCGCACCTGTCGTTTGGCGAATCTGAATAACTGAGCTGATCGCACGGTCCTAGTACCGGCGACACATCTTTCAAGTGTCTGCCTTATC
AACTGTCGATGGTAGTTTATGTGCCTACCATGGTTGTAACGGGTAACGGAGAATCAGGGTTCGACTCCGGAGAGGGAGCCTGAGAAATG
GCTACCACA
>TF1_SSU677162
ATGCATGTCTAAGTACAGCTCTCGTATAGTGAAACCGCGAATGGCTCATTAAATCAGTTACTATTTCTTAGATCTTACTTTTGTTACTT
GGATAACTGTGGTAATTCTAGAGCTAATACACGCACCAAAGCTCTGACCTTACGGGAAGAGTGCATTTATTAGAACAAAACCAATCGGG
CTCTGCCTGCTGTTTGGTGAATCTGAATAACTGAGCTGATCGCACTGGTCTAGTACCGGCGACATATCTTTCAAGTGTCTGCCTTATCA
ACTTTCGATGGTAGCTTATGTGGCTACCATGGTTGTAACGGGTAACGGAGAATAAGGGTTCGACTCCGGAGAGGGAGCCTGAGAAACGG
CTACCACA
>TF1_SSU681557
ATGCATGTCTCAGCACATGCCTATGAACGGCTAAGCCGCGAATGGCTCATTACAACAGCTATGTTTATTGGGTCTAATCAGTTACTTGG
ATAACTGTGGAAAATCCAGAGCTAATACGTGCACTAACTCCGACCGTAAGGAAGGAGGCATTTTATTAGAACAAAACCAATCGGACTTG
TCCGTGGTTTGTTGACTCTGAATAACGGCAGCGATCGTACGGTCTTTGAACCGACGACATATCTTTCAAATGTCTGCCTTATCAACTTT
CGATGGTAGTTTATGCGCCTACCACGGTTGTAACGGGTAACGGAGAATCAGGGTTTGATTCCGGAGAGGGAGCCTGAGAAATGGCTACC
ACA

>TF1_SSU688192
ATGCATGTCTAAGTGCAAGCTGAAATAAAGTGAAACCGCGAAGGGCTCATTACAACAGCCGTTGTTTCCTGGAGACTCAAAATACTTGG
ATAACTGTGGTAATTCTAGAGCTAATACATGCAGCCAAGCTCTGACCGTAAGGGAAGAGTGCATTTATTAGAACAAAACCAATCGGACT
TGTCCGTAGTTTGGTGAATCTGAATAACCTAGCTGATCGCACAGTCTTTGAACTGGCGACGCATCTTTCAAGTGTCTGCCTTATCAACT
TTCGATGGTAGTTTATGTGACTACCATGGTTATAACGGGTAATGGAGAATCAGGGTTCGACTCCGGAGAGGGAGCCTGAGAAATGGCTA
CCACA
>TF1_SSU692690
ATGCATGTCTAAGCATAAACCGAATATGGTAAAGCCGCGAATGGCTCATTACAACAGCCATAGTTTATTGGATCAGTATTTTCCACTTG
GATAACTGTGGTAATTCTAGAGCTAATACATGCGATGAAACTCTGACCTTGGTGGAAAGAGTGCATTTATTAGAACAAAACCAATCGGC
TTTTGTCGTTACTTGGTGAATCTGAATAACTCGGTTGATCGCACAGTCTTTGTACTGGCGACATATCTTTCAAGTGTCTGCCTTATCAA
CTTTCGATGGTAGTTTATACGACTACCATGGTTTTGACGGTTAACGGAGGATTAGGGTTTGACTCCGGAGAGGGAGCCTGAGATATGGC
TACCACA
>TF1_SSU694267
ATGCATGTCTAAGCTCAAGCCGAAAATGGTGAAGCCGCGAATGGCTCATTACAACAGCCTTTGTTTATTTGATCTTGAAAATTACTTGG
ATAACTGTGGTAATTCTAGAGCTAATACACGCGAAAAAATTCGGAACTTCTGTTCCGAGTGCATTTATTAGTACAAGACCAACCGGCGC
AAGCCGTAAATTGTCGAATCTGAATAACTGAGCCGATCGCATGGCCTCGTGCCGGCGACGTATCTTTCAAGTGTCTGCCCTATCAACTT
TTGATGGTAGTTTATGTGACTACCATGGTGATCACGGGTAACGGAGAATAAGGGTTCGACTCCGGAGAAGCAGCCTGAGAAACGGCTAC
TACA
>TF1_SSU694751
ATGCATGTGTAAGTATAAGCTTTTAGAACGGTGAAACCGCGAATGGCTCATTAGATCAGTTAATATTTATTAGATCGTAGAAAGTTACT
TGGATAACTGTGGTAATTCTAGAGCTAATACACGCAATCAAGCCCTGACCTGACGGGATGGGCGCATTTATTAGAATAAGACCAATTGG
CTTCGGCCATTTATTGGTGACTCTGAATAACTACGCAGATCGCATGGTCTCGTACCGGCGACATATCCTTCAAGTGTCTGCCTTATCAA
CTTTCGATGGTAGTTTACACGACTACCATGGTTGTAACGGGTAACGGAGAATAAGGGTTCGACTCCGGAGAGGGAGCCTGAGAAACGGC
TACCACA
>TF1_SSU698227
ATGCATGTCTAAGCACAAACCGATTTATGGTAAAGCCGCGAATTGCTCATTACAACAGCCATTGTTTACTGGATCTTGATAAGTTACTT
GGATAACTGTGGTAATTCTAGAGCTAATACACGCACCAAAGCTCTGACTTTATGGAAGAGCGCATTTATTAGATCAAAACCAATCAGGC
TTTGCCTGCTGTTTGGTGAATCTGAATAACTGAGCTGATCGCATTGGTCTAGTACCGGCGACATATCTTTCAAGTGTCTGCCTTATCAA
CTGTCGATGGTAGCTTATGTGGCTACCATGGTTATAACGGGTAACGGAGAATAAGGGTTCGACTCCGGAGAGGGAGCCTGAGAAACGGC
TACCACA
>TF1_SSU700188
ATGCATGTCTCAGTACAAGCTGCATTAAAGTGAAACCGCGAATGGCTCATTACAACAGCCGTTGTTCCCTAGAGACTATCAATACTTGG
ATAACTGTGGCAATTCTAGAGCTAATACACGCAGAGAAACCCCGAACTTGCGTGAGGGGTGCATTTATTAGAACAAAACCAATCGGGGC
AACCCGTAAGTTGGTGAATCTGAATAACCCAGCTGATCGCACGGTCTTTGCACTGGCGACGCATCTTTCAAGTGTCTGCCTTATCAACT
TTCGATGGTAGTTTATGTGACTACCATGGTTATAACGGGTAACGGAGAATAAGGGTTCGACTCCGGAGAGGGAGCCTGAGAAACGGCTA
CCACA
>TF1_SSU703579
ATGCATGTCTCAGTATGAGCTGAAAAAAGTGAAACCGCGAATAGCTCATTACAACAGCCATTGTTCACTGGATCTTTGTATCCTACGTG
GATAACTGTGGCAATTCTAGAGCTAATACATGCAAAAAAGCTCCGACCACTCGTTGGGAGGAGCGCATTTATTAGAACAAAACCAATCG
GGCTTCGGCCTGTCTTTGGTGACTCTGAATAACTCAGTTGATCGCACGGTCTCGTACCGGCGACTCATCTTTCAAGTGTCTGCCTTATC
AACTGTTGATGGTAGTTTACGTGACTACCATGGTTGCAACGGGTAACGGAGAATAAGGGTTCGACTCCGGAGAGGGAGCCTGAGAAACA
GCTACCACA
>TF1_SSU710679
ATGCATGTCTAAGCATAGGCCGATTAATGGTGAAGCCGCGAATAGCTCATTACAACAGCCATAGTTTATTGGATCTTCTCTCATACTTG
GATACCTGTGGTAATTCTAGAGCTAATACACGCAAGAAAACCCTGACTTCGGAAGGGGTGCATTTATTAGAACAAAACCAATCGGACTT
GTCCGTAGTTTGGTGAATCTGAATAACTCAGCTGATCGCACAGTCCTCGCACTGGCGACGTATCTTTCAAGTGTCTGCCTTATCAACTT
TCGATGGTAGTTTATATGACTACCATGGTTGTAACGGGTAACGGAGAATAAGGGTTCGACTCCGGAGAGGGAGCCTGAGAAACGGCTAC
CACA
>TF1_SSU734804
ATGCATGTCTGAGCACAAGCTCAAGAAAAGTGAAGCCGCGAATAGCTCATTACAACAGCCACTTTTCACTTGATCTTGATATCCTACTT
GGATAACTGTGGCAATTCTAGAGCTAATACATGCATTGAAGCTCTGACCAGCTTGCTGGGAAGAGCGCATTTATTAGAACAAAACCAAT
TGGACTTCGGTTCGTAATTGGTGACTCTGAATAACTCAGATGATCGCACGGTCTTGTACCGGTGACAGATCATTCAAGTGTCTGCCTTA
TCAACTGTTGATGGTAGTTTATATGACTACCATGGTTGCAACGGGTAACGGAGAATAAGGGTTCGTCTCCGGAGAGGGAGCCTGAGAAA
CGGCTACCACA
>TF3_SSU956521
ATGCGTGTCTAGGTACAAGCCTAAAAACGGTAAAGCCGCGAATGGCTCATTACAACAGCCATAGTTTATTGGATCTTGACTATCTTACT
TGGATAACTGTAGTAATTCTAGAGCTAATACACGCACCAAAGCCCAGACCTTACGGAACGGGCGCATTTATTAGACCAAAACCAATCGG
GCTTCGGCCCGTCTTTGGTGACTCTGAATAACTACGCTGAGTGCACGGTCCTCGAACCGGCACCGTATCTTTCAAGTGTCTGCCTTATC
AACTGTCGATGGTAGTTTACGTGACTACCATGGTTGTAATGGGTAACGGAGAATAAGGGTTCGACTCCGGAGAGGGAGCCTGAGAAATG
GCTACCACA
>TF3_SSU960449
ATGCATGTGTAAGTACAAGCTTTTAGAACGGTGAAACCGCGAATGGCTCATTAGATCAGTTAATATTTATTAGATCGTAGAAAGTTACT
TGGATAACTGTGGTAATTCTAGAGCTAATACACGCAAAAGAACTTGGAACGTAGGTTCCGGGTGCATTTATTAGTACAAGACCATCAGG
GCTCGTCCCTTCCAATGGTGAATCTGAATAACTGAGCCGATCGCATGGTCTCGCACCGGCGACGTATCTTTCAAGTGTCTGCCCTATCA
ACTTTCGATGGTAGTTTATATGACTACCATGGTTATAACGGGTAACGGAGAATAAGGGTTCGACTCCGGAGAAGCAGCCTGAGAAACGG
CTACTACA
>TF3_SSU966338
ATGCATGTCTAAGCACAAACGAAATTAACGTGAAGCCGCGAAAAGCTCATTACAACAGCCGTCGTTTCTTGGATCTCCGAACTTACTTG
GATAACTGTGGTAATTCTAGAGCTAATACATGCAATCGAGTCCTGAGCGTAAGCGATGGGCGCATTTATTAGTAACAAGACCAATCGGT
GCTTGCACCGTGGTTTGGTGAATCTGAATAACTGAGCAGATCGCTTCGGTCTTTGTACCGGCGACGTATCTTTCAAGTGTCTGTTTTAT
CAACTTTCGATGTTAGTTTATGTGACTAACATGGTTGTCACGGATAACGGAGAATAAGGGTTCGACTCCGGAGAGGGAGCATGAGAAAC

GGCTACCACA
>TF4_SSU144249
GTGCATGTCTAAGCATGAGCCATCAAATGGTGAAGCCGCGAACAGCTCATTACAACAGCCATAGTTCATTGGACTTCTCTCAATACTTG
GATATCTGTAATAATTTTAGAGCTAATACACGCAAGCAAACTCCAATCTCACGAGCGGAGTGCATTTATTAGAACAAAACCAATCGGGC
TTGCCCGTGCGTTTGGTGAATCTGAATAACTCAGCTGATCGCACAGTCTAGCACTGGCGACATATCTTTCAAGTGTCTGCCTTATCAAC
TGTCGATGGTAGTTTATATGACTACCATGGTTGTAACGGGTAACGGAGAATCAGGGTTTGACTCCGGAGAGGGAGCCTGAGAAACGGCT
ACCACA
>TF4_SSU150234
ATGCATGTCTAAGCATGAGCCCTATAATGGTGAAGCCGCGAATGGCTCATTACAACAGCCGTTGTTTCTTGGATCTTGATTCACTTGGA
TAACTGTGGTAATTCTAGAGCTAATACATGCAACTCAGCTCCAACTGAGAAGAAGGAGCGCATTTATTTGACCAAAACTGACTAGGTTT
CGACCTAAAACTTGGTGACTCTGAATAACTCTGCTGATCACACAGTCTTGCACTGGTGACATATCTTTCAAGTGTCTGCCCTATCAACT
TTCGATGGTAGTTTATATGCCTACCGTGGTTGTAACGGGTAACGGAGAATAAGGGTTCGACTCCGGAGAGGGAGCCTGAGAAATGGCTA
CCACA
>TF5_SSU410031
ATGCATGTCTAAGCAGAAGCCGCAATACGGTGAAACCGCGAATAGCTCATTACAACAGCCTTAGTTTCTTGGATCTTCAACAGTTACTT
GGATAACTGTGGTAATTCTAGAGCTAATACACGCATGAAAATCTCGGCTTTGCGGTTGGGATGCATTTATTAGTACAAAACCAATCGAG
CGCGAGCTCGTTGTTGGTGAATCTGAATAACTTTGCTGATCGCACGCTCTTCGTAGTGGCGACGCATCTTTCAAGTGTCTGGCTTATCA
ACTTTCGATGGTAGTTTATACGACTACCATGGTTGTAACGGGTAACGGAGAATAAGGGTTCGACTCCGGAGAGGGAGCCTGAGAAACGG
CTACCACA
>TF5_SSU419519
ATGCATGTCTAAGCACAAGCCGAAAATGGTAAAGCCGCGAATGGCTCATTACAACAGCCTTTGTTTATTTGATCTTGAAATCCTACTTG
GATAACTGTGGTAATTCTAGAGCTAATACACGCATCTAAACTCGAGACGCCCGTCACGAGTGCATTTATTAGTACAAAACCAATCGGGC
TTGCCCGTTCTTTGGTGAATCTGAATAACTGAGCCGATCGCACGGTCATTGTACCGGCGACGTATCTTTCAAGTGTCTGCCCTATCAAC
TTTCGATGGTAGTTTATATGACTACCATGGTTATAACGGGTAACGGAGAATAAGGGTTCGACTCCGGAGAAGCAGCCTGAGAAACGGCT
ACCACA
>TF5_SSU430294
ATGCATGTCTAAGCATAAACTAAACTAAAGTGAAGCCGCGAATAGCTCATTACAACAGCCGTTGTTTCTTGGATCTCCGTATTACTTGG
ATAACTGTGGTAATTCTAGAGCTAATACACGCAATCGAGCTCCGACCTTACGGGACGAGCGCATTTATTAGATCAAAACCAATCAGTGC
TTGCACTGTAGTCTGGTGAATCTGAATAACTGAGCAGATCGCTTCGGTCTTGGTACCGGCGACATATCCTTCAAGTGTCTGCCTTATCA
ACTTTCGATGGTAGTTTATGTGCCTACCATGGTTGTAACGGGTAACGGAGAATTAGGGTTCGACTCCGGAGAGGGAGCCTGAGAAACGG
CTACCACA
>TF5_SSU437076
ATGCATGTGTAAGTACAAGCTTTTAGAACTGTGAAACCGCGAATGGCTCATTAGATCAGTTAATATTTATTAGATCGTAGAAAGTTACT
TGGATAACTGTGGTAATTCTAGAGCTAATACACGCCTTGAAGCTCTGACCTTCGGGGACGAGCGCATTTATTAGAACAAAACCAATGGG
GTTTGCCCCTCGGTTGGTGACTCTGAATAACTACTCCGATCGCACGGTCTCGCACCGGCGACGCATCTTTCAAGTGTCTGCCTTATCAA
CTGTCGATGGTAGTTTATGTGCCTACCATGGTTGTAACGGGTAACGGAGAATAAGGGTTCGACTCCGGAGAGGGAGCCCGAGAAACGGC
TACCACA
>TF5_SSU444034
ATGCATGTCTAAGCACAAGCCGCTTGATGGTAAAGCCGCGAATGGCTCATTACAACAGCTATTGTTTATTAGATCTTACCATCCTACTT
GGATAACTGTTGTAATTCTAGAGCTAATACACGCATCAAAAACGGGACCTAAGGGAACCGTTGCATTTATTAGAACAAAACCAATCGGG
CTTCGGCCCGTCATTTGGTGAATCTGAATAACTCTGCCGATCGCACGGTCCACGAACCGGCGACGCATCTTTCAAATGTCTGCCTTATC
AACTTTCGATGGTAGTTTATGCGCCTACCATGGTTGTAACGGGTAACGGAGAATCAGGGTTTGACTCCGGAGAGGGAGCCTGAGAAACG
GCTACCACA
>TF5_SSU446087
ATGCATGTCTAAGCAGAAGCCGAACAATGGCAAAGCCGCGAATGGCTCATTACAACAGCTGTTGTTTATTTGATCTTGAATTCCTACAT
GGATAACTGTGGTAATTCTAGAGCTAATACATGCTTACAAACTCATTTCCTTGGATCTGAGTGCATTTATTAGAACAAAACCAATCGGG
CTTGCCCGTTCATTGGTGAATCTGAATAACTATGCCGATCGCACGGTCTTCGCACCGGCGACGTATCTTTCAAGTGTCTGCCCTATCAA
CTTTCGATGGTAGTTTATGTGACTACCATGGTTATAACGGGTAACGGAGAATAAGGGTTCGACTCCGGAGAAGCAGCCTGAGAAATGGC
TACTACA
>TF5_SSU453472
ATGCATGTCTCAGCACAAGCCAATATATGGTAAAGCCGCGAATGGCTCATTACAACAGCCACTGTTTATTAGATCATCCTTCTTACTTG
GATAACTGTGGAAAAGCTAGAGCTAATACATGCTACAAGCTCTGACCTTACGGAAGGAGCGCATTTATTAGAACAAAAACAATCGGACT
TTGTCCGTCATTTGGTGAATCTGAATAACTATGCAGATCGCACGGTCTTAGAACCGGCGACATATCTTTCAAATGTCTGCCCTATCAAC
TTTCGACGGTATGTGATATGCTTACCGTGGTTGCAACGGGTAGCGGGGAATCAGGGTTCGATTCCGGAGAGGGAGCATGAGAAACGGCT
ACCACA
>TF5_SSU457543
ATGCATGTGTAAGCATAAGCCGATTAAATGGTGAAGCCGCGAATGGCTCATTACAACAGCCATAGTTTATTATATTTTTTCTTTTACTT
GGATAACTGAGGTAATTCTTGAGCTAATACACGCATCAAAGCTTCGACCTTACGGAAGGAGCGCATTTATTAGAACAAAACCAATCGGA
CTTCGGTCCGTTACTTGGTGACTCAGAATAACTCTGTGGATCGCATGGTCTTAGCACCGGCGACGTATCTTTCAAGTGTCTGCCTTATC
AACTTTCGTTGGTAATTTATGTGATTACCAAGGTTGTAACGGGTAACGGAGAATCAGGGTTTGATTCCGGAGAGGGAGCCTGAGAAATG
GCTACCACA
>TF5_SSU459305
ATGCATGTCTTAGTACAGACTATCTCACAGTGAAACTGCGAATGGCTCATTAAATCAGCTAAGGTTCCTTAGATCGTACAATCCTACAT
GGATACCTGTGGTAATTCTACAGCTAATACACGCATCAAAACCCAACCTTACGGTGGGGTGCGTTTGTTACTTCAAACCAATCGGACTT
CGGTCTGAAATCAAGTGATATTGAACAATTTAGCTGATCGCACGGTCTGAGAACCGGCGACATATCCTTCAAATGTCTGCCTTATCAAC
TTTCGATGGTAGATTACGCGCCTACCATGGTTGTAACGGGTAACGGAGAATCAGGGTTTGATTCCGGAGAGGGAGCCTGAGAAACGGCT
ACCACA
>TF5_SSU466315
ATGCATGTCTAAGCATAAACTAATCTAAAGTGAAGCCGCGAATAGCTCATTACAACAGCCGTTGTTTCTTGGATCTCCGTCTTACTTGG
ATAACTGTGGTAATTCTAGAGCTAATACATGCAGCTGAGATCTGACTTTACAGGAAGATCGCATTTATTAGATCAAAACCAATCGGCTT
CGGCCGTTGCTGGTGAATCTGAATAACTACGCAGATCGCTTAGGTTTTATACCGGCGACGTGTCCTTCAAGTGTCTGCCTTATCAACTT

TCGATGGTAGTTTCTACGCCTACCATGGTTGCGACGGGTAACGGAGAATCAGGGTTCGATTCCGGAGAGGGAGCCTGAGAAACGGCTAC
CACA
>TF6_SSU33463
ATGCATGTGTAAGCATGAACCATTTAATGGTGAAGCCGCGAATGGCTCATTACAACAGCCATAGTTTATTAGATTTCTTTTTTACTTGG
ATAACTGTGGTAATTCTAGAGCTAATACACGCAGCAAAGCTTCGACCTTACGGAAGGAGCGCATTTATTAGAACAAGACCAATCGTACT
TCGGTACGTATTTTGGTGAATCTGAATAACTTAGTCGATCTCATGGTCTTAGCACCGGAGACGCATCTTTCAAGTGTCTGCCTTATCAA
CTTTCGATGGTAGTTTATGCGCCTACCATGGTTGTAACGGGTAACGGAGAATCAGGGTTTGATTCCGGAGAGGGAGCCTGAGAAATGGC
TACCACA
>TF6_SSU33935
ATGCATGTCTAAGCAGAAGCCGAACAATGGCAAAGCCGCGAATAGCTCATTACAACAGCCATTGTTTACTTGATCTTGAAATCCTACTT
GGATAACTGTGGTAATTCTAGAGCTAATACACGCAATAAGCTCCGACCTCAGGGGAGGAGTGCATTTATTAGAACAAAACCAATCAGAC
CTCGGTCTGTCTCTTGGTGAATCAGAATAACTCAGCTGATTGCACAGTCTTGTACTGGCGACGTATCTTTCAAGTGTCTGCCTTATCAA
CTGTTGATGGTAGTTTATGCGACTACCATGGTTGTAACGGGTAACGGAGAATAAGGGTTCGACTCCGGAGAGGGAGCCTGAGAAACGGC
TACCACA
>TF6_SSU36442
ATGCATGTCTAAGCACAAGCCGAATATGGTGAAGCCGCGAATGGCTCATTACAACAGCCTTTGTTTATTTGATCTTGAAATCCTACTTG
GATAACTGTGGTAATTCTAGAGCTAATACACGCAACAAAACTTTGAACGTAAGTTCTTGGTGCATTTATTAGTACAAAACCTTCCGGAC
TTCGGTTCGTAAACTGGTGAATCTGAATAAATTAGCCGATCGCATGGCCTTCGCGCTGGCGACGTATCTTTCAAGTGTCTGCCCTATCA
ACTTTCGATGGTAGTTTATATGACTACCATGGTTATAACGGGTAACGGAGAATAAGGGTTCGACTCCGGAGAAGCAGCCTGAGAAACGG
CTACTACA
>TF6_SSU37421
ATGCATGTGTAAGTACAAGCTTTTAGAACGGTGAAACCGCGAATGGCTCATTAGATCAGTTAATATTTATTAGATCGTAGAAAGTTACT
TGGATAACTGTGGTAATTCTAGAGCTAATACATGCAACCAAGCTCCAACCTTTCGGAAGGAGCGCATTTATTATACCAAGACCAATCGG
GCTTTGCCCGCTATCTGGTGACTCTGAATAACTTTGCTGATCACATGGTCATAGTACCGGTGACATATCTTTCAAGTGTCTGCCCTATC
AACTTTCGATGGTAGTTTATGTGCCTACCATGGTTGTAACGGGTAACGGAGAATAAGGGTTCGACTCCGGAGAGGGAGCCTGAGAAATG
GCTACCACA
>TF6_SSU41803
ATGCATGTCTAAGTACAAGCCTCATTAAGGTGAAACCGCGAATGGCTCATTAAATCACACCTAATATACTGGATAGTATACAGTTACTT
GGATAACTGCGGTAATTCTGGAGCTAATACATGCAGCTGAGATCTGACTTTACAGGAAGATCGCATTTATTAGATCAAAACCAATCGGC
TTCGGCCGTTGCTGGTGAATCTGAATAACTACGCAGATCGCTTAGGTTTTATACCGGCGACGTGTCCTTCAAGTGTCTGCCTTATCAAC
TTTCGATGGTAGTTTCTACGCCTACCATGGTTGCGACGGGTAACGGAGAATTAGGGTTCGACTCCGGAGAGGGAGCCTGAGAAACGGCT
ACAACA
>TF6_SSU47996
ATGCATGTCTAAGCACAAACTGATTAATTGTGAAGCCGCGAATGGCTCATTACAACAGCCATTGTTTACTGGATATCTCATTACTACAT
GGATAACTGTGGTAATTCTACAGCTAATACACGCATCAAAACCCCAACTTTCGAAGGGGTGCGTTTGTTACTTCAAATCAATCGGACTT
CGGTCTGGTTTCAACTGAGATTGAACAATTTAGCTGATCGCACGGTCTAAGAACCGGCGACATATCCTTCAAACGTCTGCCTTATCAAC
TTTCGATGGTAGATTATGCGCCTACCATGGTTGTTACGGGTAACGGAGAATCAGGGTTTGATTCCGGAGAGGGAGCCTGAGAAACGGCT
ACCACA
>TF6_SSU48167
ATGCATGTCTAAGCAGAAGCCGCACAATGGTAAAGCCGCGAATGGCTCATTACAACAGCCGTCGTTTCTTGGATCTCTTGTTTTACTTG
GATAACTGTGGTAATTCTAGAGCTAATACACGCACTAAAGCTCTGACCTTACGGGACGAGCGCATTTATTAGAACAAAACCAATCGGGT
TTCGGCCCGTCCGTTGGTGACTCTGAATAACTACGCCGATCGCACGGTCTCGTACCGGCGACGTATCTTTCAAGTGTCTGCCTTATCAA
CTTTCGATGGTAGTTTATGTGCCTACCATGGTTGTAACGGGTAACGGAGAATAAGGGTTCGACTCCGGAGAGGGAGCCTGAGAAACGGC
TACCACA
>TF6_SSU53456
ATGCATGTCTAAGTATGAACTATCTATTGTGAAACCGCGAATGGCTCATTACAACAGCCATAGTTTACTGGATATATTCCTTTACATGG
ATAACTGTGGTAATTCTACAGCTAATACACGCATCAAAGCCCCGACTTTATGAAGGGGCGCGTTAGTTACTTCAAACCGATCGGTCTTC
GGACTGTATCCAAGTGAGATTGAACTATTTAGCTGAGCGCACGGTCTAAGCACCGGCGCCATATCCTTCAAATGTCTGCCTTATCAACT
TTCGATGGTAGATTATGCGCCTACCATGGTTGTAACGGGTAACGGAGAATCAGGGTTTGATTCCGGAGAGGGGGCCTGAGAAATGGCCA
CCACA
>TF6_SSU54250
ATGCACGTTTCAATACAAGCCTTACTAAGGTGAAATCGCGAATGGCTCATTACAACAGCCATTGTTTCTTGGATCTTATCTTTTACTTG
GATAACTGTGGTAATTCTAGAGCTAATACGTGCCACCAATCCCGACGCAAGAAGGGATGCATTTATTAGAACTAAACCGACCGGGCTCA
GCCCGCTGTTTGGTGAATCTGAATAACTCTGCAGATCGCATGGTCTCGCACCGGCGACATATCCTTCAAGTGTCTGCCTTATCAACTTT
CGATGGTAGTTTATGTGACTACCATGGTTGTCACGGGTAACGGAGAATTAGGGTTCGACTCCGGAGAGGGAGCCTGAGAAACGGCTACC
ACA
>TF6_SSU58877
ATGCATGTCTAAGCACAAGCCGAAAATGGTAAAGCCGCGAATGGCTCATTACAACAGCCTTTGTTTATTTGATCTTGAAATCCTACTTG
GATAACTGTGGTAATTCTAGAGCTAATACAAGCGATTAAACTCCAACCTTTTGGAAGGAGTGCATTTATTAGTACAAAACCAATCGGGG
TAAAACCCGTGGTTTGGTTAATCTGAATAACTCTGTCGATCGCACGGTCTTTGTACCGGCGACATATCTTTCAAGTGTCTGCCCTATCA
ACTGTCGATGGTAGTTTATATGACTACCATGGTTGTAACGGGTAACGGAGAATAAGGGTTCGTCTCCGGAGAAGCAGCCTGAGAAACGG
CTACTACA
>TF6_SSU74955
ATGCATGTCTAAGCATGAGCCATCAAATGGTGAAGCCGCGAACAGCTCATTACAACAGCCATAGTTCATTGGACTTCTCTCAATACTTG
GATATCTGTAATAATTTTAGAGCTAATATACGCAAGCAAACTCCAATCTCACGAGCGGAGTGCATTTATTAGAACAAAACCAATCGGGC
TTGCCTGTGCGTTTGGTGAATCTGAATAACTCAGCTGATCGCACAGTCTAGCACTGGCGACGTATCTTTCAAATGTCTGCCCTATCAAC
TTTCGACGGTATGTGATATGCTTACCGTGGTTGCAACGGGTAGCGGGGAATCAGGGTTCGATTCCGGAGAGGGAGCATGAGAAACGGCT
ACCACA
>TF6_SSU82210
ATGCATGTCTATGCATAAGCCAATTTATGGTGAAGCCGCGAATGGCTCATTACAACAGCTTTCATTTCTTGGGTGTCATTTTACTTGGA
TAACTTTGTCAACCCAGAGCTAATACATGCACAAAAGCCTTGACTTATGGAAAGGCGCAGTTATTTGATCAAAACCAATCAGGCTTGCC

TGAATTTGATGACTCTGAGTAAACTTGCAGATCGCACAGTCCTAGTACTGGCGACAAACTCTTCAAGTGTCTGCCTTATCAACTTTCGA
TGGTAGTTTCAGTGCCTACCATGGTTACAATGGGTAACGGAGAATAAGGGTTCGACTCCGGAGAGGGAGCCTGAGAAACGGCTACCACA
>TF6_SSU84268
ATGCATGTCTCAGTGCAAGTCCATTCAGGACGAAACCGCGAACGGCTCATTACAACAGCTATAATCTACGGGGAGTTTCCATACATGGA
TAACTCTGTCAACCCAGAGCTAATACATGCACAAAAGCCTCACCTCATGGCTGGGCGCATCTATTATACCAAAACCAACCGGGCTCTGC
CCGAGTCTTGGTGACTCTGAATAGAGAGTTAATGACGCAGTCTTAGTACTGGTCATGTTCCCCACGAGTGTCTGCCTTATCAACTTTCG
ATGGTAGTTTATGTGCCTACCATGGTTGCAACGGGTAACGGAGAATAAGGGTTCGACTCCGGAGAGGGAGCCCGAGAAACGGCTACCAC
A
>TF6_SSU98667
ATGCATGTTCCAGCAGAAACTGAATATAGTGAAGCCGCGAATAGCTCATTACAACAGCCATTGTTCATTGGAGTTGAAGACAATTTGGA
TAACCCTGTTAAATCAGAGCTAATACACGCAAAAAAGCTTGTCCTTTGCGGTTTAAGTGCATTTATTAGAACAAAATCATCCGGCCTTT
GGCCGTATTTGGTGAATCTGAATAACTTTGCTGATCGCACGGTCTAGTACTGGCGACGTATCTTTCAAGTGTCTGCCTTATCAACTTTC
GACGGTAGTTTATGTGCCTACCGTGGTTATAACGGGTAACGGAGAATTAGGGTTCGACTCCGGAGAGGGAGCCTGAGAAATGGCTACCA
CA
>TS1_SSU270885
ATGCATGTCTAAGCACAAGCTGAAAATGGTGAAGCTGCGAATGGCTCATTACAACAGCCTTTGTTTATTTGATCTTGAAATCCTACTTG
GATAACTGTGGTAATTCTAGAGCTAATACATGCTATCAAACTGAAGGCTCTGCCTTCAGTGCATTTATTAGTACAAAACCAATCAGGTT
TTACCTGCCAATTGGTGAATCAGAATAACTGTGCAGATCACATAGCCTATGAGCTGGTGACATATCTTTCAAGTGTCTGCCCTATCAAC
TTTCGATGGTAGTTTATATGACTACCATGGTTATAACGGGTAACGGAGAATAAGGGTTCGACTCCGGAGAAGCAGCCTGAGAAATGGCT
ACTACA
>TS1_SSU284163
ATGCATGTCTAAGCACAAGCCGAATATGGTGAAGCCGCGAATGGCTCATTACAACAGCCTTTGTTTATTTGATCTTGAAATCCTACTTG
GATAACTGTGGAAATTCTAGAGCTAATACACGCAAAAGAACTCGGAACGTAGGTTCCGGGTGCATTTATTAGTACAAGACCATCAGGGC
TCGTCCCTTCCAATGGTGAATCTGAATAACTGAGCCGATCGCATGGTCTCGCACCGGCGACGTATCTTTCAAATGTCTGCCCTATCAAC
TTTCGACGGTATGTGATATGCTTACCGTGGTTGCAACGGGTAGCGGGGAATAAGGGTTCGATTCCGGAGAGGGAGCATGAGAAACGGCT
ACCACA
>TS2_SSU821962
ATGCAAGTCTAAGCACAAGCCGTACAATGGTAAAGCCGCGAATAGCTCATTACAACAGCCATAGTTTATTAGATAGTTCTTTACTACAT
GGATAACTGTGGTAATTCTAGAGCTAATACATGCTACAAACGGCGACCTTTAGTTAGGAAGCCGCGCTTTTATTAGAACAAAACCAATC
GCTCTTTCGGGAGCGTCAATTGGTGAGTCTAAATAACATAGCAGATCGCACGGTCTGGGCACCGGCGACGAATCTTTCAAATGTCTGCC
TTATCAACTTTCGATGGTAGTTTACATGCCTACCATGGTGATAACGGGTAACAGAGAATTAGGGTTTGACTCTGGAGAGGCAGCCTGAG
AGACGGCTACCACA
>TS2_SSU823349
ATGCATGTCTAAGTACAGACTATATCACAGTGAAACTGCGAATGGCTCATTAAATCAGCTAAGGTTCCTTAGATCGTACAATCCTACAT
GGATAACTGTGGTAATTCTAGAGCTAATACATGCTACAAACGGCGACCTTTAGTTAGGAAGCCGCGCTTTTATTAGAACAAAACCAATC
GCTCTTTCGGGAGCGTCAATTGGTGAGTCTAAATAACATAGCAGATCGCACGGTCTTGGCACCGGCGACGAATCTTTCAAATGTCTGCC
TTATCAACTTTCGATGGTAGTTTACATGCCTACCATGGTGATAACGGGTAACAGAGAATTAGGGTTTGACTCTGGAGAGGCAGCCTGAG
AGACGGCTACCACA
>TS3_SSU475561
ATGCATGTCTAAGCACAAACTGAAATAAAGTGAAGCCGCGAATAGCTCATTACAACAGCCATTGTTTACTTGATCTTGAAATCCTACTT
GGATAACTGTGGTAATTCTAGAGCTAATACACGCAATTAAGCTCTGATCCTTTTGGTGACGAGTGCATTTATTAGAACAAAACCAATCG
GACTTCGGTCTGTTGTTGGTGAATCTGAATAACTCAGCTGATCGCACGGTCTTGTACCGGTGACGCATCTTTCAAGTGTCTGCCCTATC
AACTTTCGACGGTATGTGATATGCTTACCGTGGTTGCAACGGGTAGCGGGGAATCAGGGTTCGATTCCGGAGAGGGAGCATGAGAAACG
GCTACCACA
>TS3_SSU489684
ATGCATGTCTAAGCACAAGCCGAATATGGTGAAGCCGCGAATGGCTCATTACAACAGCCTTGGTTTATTTGATCTTGAAATCCTACATG
GATAACTGTGGTAATTCTAGAGCTAATACACGCATACAAGCTTCAGCCTTACGGCTGTGAGCGCATTTATTAGTACAAAACCAATCGGG
CCTTGCCCGTTGTTTGGTGAATCTGAATAACTGAGCCGATCGCATGGTCTCTGTACCGGCGACGTATCATTCAAGTGTCTGCCCTATCA
ACTTTCGATGGTAGTTTATGTGACTACCATGGTTACAACGGGTAACGGAGAATAAGGGTTCGACTCCGGAGAAGCAGCCTGAGAAACGG
CTACTACA
>TS3_SSU503133
ATGCAAGTCTAAGCACAAGCCGTTAAATGGTAAAGCCGCGAATAGCTCATTACAACAGCCATAGTTTATTAGATAGTTCCTTACTACAT
GGATAACTGTGGTAATTCTAGAGCTAATACATGCTTAAAAGAACGACCTCGCAAGAGGTTGTTCTGCACTTATTAGAACAAAACCAATC
GCGCTGCGGCAACGTAGTGCGTTATTTGGTGAATCTGAATAACTTGGCGGATCGCACGGTCTAGTACCGGCGACGCATCTTTCAAATGT
CTGCCTTATCAACTGTCGATGGTAGTTTACATGCCTACCATGGTGATAACGGGTAACAGAGAATAAGGGTTTGACTCTGGAGAGGGAGC
CTGAGAGACGGCTACCACA
>TS3_SSU508400
ATGCAGGTCTGAGCACGAGCTCAAGAAAATAATGGATGGATAATTGGATAACTGTGGCAATTCTAGAGCTAATACATGCATTGAAGCTC
CGACCAGCTTGCTGGGAAGAGCGCATTTATTAGAACAAAACCAATCGGACTTCGGTTCGTTATTGGTGACTCTGAATAACTCAGTCGAT
CGCACGGTCTTGTACCGGCGACAGATCATTCAAGTGTCTGCCTTATCAACTGTTGATGGTAGTTTATGTGACTACCATGGTTGCAACGG
GTAACGGAGAATAAGGGTTCGTCTCCGGAGAGGGAGCCTGAGAAATAGCTACCACA
>TS4_SSU543236
ATGCATGTCTCAGCACATACCAATATATGGCAAAGCCGCGAATGGCTCATTACAACAGCCGCATTTTATTAGATAATCCTATTTACTTG
GATAACTGTGGAAAAACTAGAGCTAATACATGTCATAAGCTTCGACCTTACGGAAGAAGTTCATTTATTAGAACAAAACCAATCGGACT
TTGTCCGTTACTTTGTTGACTCTGATTAACTTCATGATCGCACGGTCATAGAACCGGCGACATATCTTTCAAATGTCTGCTTTATCAAC
TTTCGATGGTAGTTTATGCGCCTACCATGGTTGTAACGGGTAACGAAGAATCAGGGTTTGATTTCGGAGAGGGAGCCTGAGAAACGGCT
ACCACA
>TS4_SSU544032
ATGCATTTCTAAGCACAAGCCGAATATGGTGAAGCCGCGAATGGCTCATTACAACAGCCTTTGTTTATTTGATCTTGAAATCCTACTTG
GATAACTGTGGTAATTCTAGAGCTAATACACGCAAAAGAACTCGGAACGTAGGTTCCGGGTGCATTTATTAGTACAAGACCATCAGGGT
TCGTCCCTTCCAATGGTGAATCTGAATAACTGAGCCGATCGCATGGTCTCGCACCGGCGACGTATCTTTCAAGTGTCTGCCCTATCAAC

TTTCGATGGTAGTTTATATGACTACCATGGTTATAACGGGTAACGGAGAATAAGGGTTCGACTCCGGAGAAGCAGCCTGAGAAACGGCT
ACTACA
>TS5_SSU874117
ATGCATGTATCAGCACAAGCCGTAATATGGTGAAGCCGCGAATAGCTCATTATAACAGTCGTAGTTTATTAGAAAGTCTGTACTGGATA
ACTGTGGTAATTCCAGAGCTAATACATGTTCCAAGCCCCAACTAACGAAGGGGTGCATTTATTAGAACAAGGCCGATCAGACTTTGTCT
GTCTCAGGTTGACTCTGAATAACTTTGCTAATCGCACAGTCTTTGCACTGGCGATGTATCTTTCAAATGTCTGCCCTATCAACTTTCGA
CGGTATGTGATATGCTTACCGTGGTTGCAACGGGTAGCGGGGAATCAGGGTTCGATTCCGGAGAGGGAGCATGAGAAACGGCTACCACA
>TS5_SSU875407
ATGCATGTCTAAGTACAGACTATATCACAGTGAAACTGCGAATGGCTCATTAAATCAGCTAAGGTTCCTTAGATCGTACAATCCTACTT
GGATAACTGTGGTAATTCTAGAGCTAATACACGCCTTGAAGCTCTGACCTTCGGGGACGAGCGCATTTATTAGAACAAAACCAATGGGG
TTCGCCCCTCGGTTGGTGACTCTGAATAACTACTCCGATCGCACGGTCTCGCACCGGCGACGCATCTTTCAAGTGTCTGCCTTATCAAC
TGTCGATGGTAGTTTATGTGCCTACCATGGTTGTAACGGGTAACGGAGAATAAGGGTTCGACTCCGGAGAGGGAGCCCGAGAAACGGCT
ACCACA
>TS5_SSU881546
ATGCATGTCTAAGCAGAAACTATTTTAAAGTGAAGCCGCGAAAAGCTCATTACAACAGCCGTCGTTTCTTGGGTCTCCGAATTTACTTG
GATAACTGTGGTAATTCTAGAGCTAATACATGCAATCGAGCTCTGAACGTAAGTGATGGGCGCATTTATTAGTAACAAAACCAATCGGT
TGCTTGCAACCGTGGTTTGGTGAATCTGAATAACTTAGCAGATCGCTTCGGTCTTTGTACCGGCGACATATCTTTCAAATGTCTGCCCT
ATCAACTTTCGATGGTACGTTATGCGCCTACCATGGTCGTAACGGGTAACGGAGAATCAGGGTTCGATTCCGGAGAGGGAGCCTGAGAA
ATGGCTACCACA
>TS5_SSU900338
ATGCATGTCTATGCACAAGCCGATAAATGGCAAAGCCGCGAATGGCTCATTACAACAGCCACTGTTCACTTGATCTGTATCATATCCTA
CTTGGATAACTGTGGTAATTCTAGAGCTAATACACGCACCCATTCTCCGACCGCAAGGGACGAGAGCATTTATTAGAACAAAACCAATC
GGCTTCGGTCGTTCGTTTGTGACTCTGAATAACTTTGCTGATCGTACGGTCTTTGTACCGACGACGCATCTTTCAAGTGTCTGCCTTAT
CAACTTTCGATGGTAAGTTCCTTGCTTACCATGGTTGTAACGGGTAACGGAGAATCAGGGTTCGACTCCGGAGAGGGAGCCTGAGAAAC
GGCTACCACA
>TS5_SSU901243
ATGCATGTCTATGCACACGCCGATTAATGGTAAAGCCGCGAATAGCTCATTACAACAGCCTCTGTTTATTAGATCTTTTTATCCTACTT
GGATAACTGTGGCAATTCTAGAGCTAATACACGCACCAAAACTCCGACCTTGCGGAAGGAGTGCATTTATTAGACCAAAACCAATGCAG
GCTTGTCCTGTTACTTTGGTGAATCTGAATAACCTTTGCCGAGTGAATGGTCTTTGAACCGTCACCATATCTTTCAAATGTCTGCCTTA
TCAACTTTCGACGGTAGTTTATATGACAACCGTGGTTGTAACGGGTAACGGGGAATCAGGGTTCGATTCCGGAGAGGGAGCATGAGAAA
CGGCTACCACA
>TS6_SSU559765
ATGCATGTCTTAGTACAGACTATCTCACAGTGAAACTGCGAATGGCTCATTAAATCAGCTAAGGTTCCTTAGATCGTACAATCCTACTT
GGATAACTGTAGTAATTCTAGAGCTAATACATGCTTAAAAGAACGACCTCGCAAGAGGTTGTTCTGCACTTATTAGAACAAAACCAATC
GCGCTGCGGCAACGTAGTGCGTTATTTGGTGAATCTGAATAACTTGGCGGATCGCACGGTCTAGTACCGGCGACGCATCTTCCAAATGT
CTGCCTTATCAACTGTCGATGGTAGTTTACATGCCTACCATGGTGATAACGGGTAACAGAGAATAAGGGTTTGACTCTGGAGAGGGAGC
CTGAGAGACGGCTACCACA
>TS6_SSU570763
ATGCATGTCTAAGTACAGACTATATCACAGTGAAACTGCGAATGGCTCATTAAATCAGCTAAGGTTCCTTAGATCGTACAATCCTACTT
GGATAACTGTAGTAATTCTAGAGCTAATACACGCATCAAGCTCTGACCTCATGGAATGAGCGCATTTATTAGAACAAAAACCAATCAGG
CTATGCCTGTTTTTGGTGGATCTGAATAACTCAGCTGACCGTATGCTCTCGTAGCGACGGCGATTCCTCCAAGTATCTGCCTTATCAAC
TGTTGATGGTAGTTTATGTGACTACCATGGTTGTAACGGGTAACGGAGAATAAGGGTTCGACTCCGGAGAGGGAGCCTGAGAAACGGCT
ACCACA
>TS6_SSU587229
ATGCATGTCTAAGCATAAATGAATTCATAGTGAAGCCGCGAATAGCTCATTACAACAGCCATCGTTTAATGGATATATTTTTACATGGA
TAACTGTGGTAATTCTACAGCTAATACACGCATCAAAACCCAACCTTACGGTGGGGTGCGTTTGTTACTTCAAACCAATCGGACTTCGG
TCTGAAATCAAGTGATATTGAACAATTTAGCTGATCGCACGGTCTGAGAACCGGCGACATATCCTTCAAATGTCTGCCTATCAACTTTC
GATGGTAGATTACGCGCCTACCATGGTTGTAACGGGTAACGGAGAATCAGGGTTTGATTCCGGAGAGGGAGCCTGAGAAACGGCTACCA
CA
>HE6_SSU372021
ATGCATGTCTAAGCACAAACTTTATGAGTGAAGCCGCGAAAAGCTCATTACAACAGCCGTCGTTTCTTGGATCTCCATTCTCTACTTGG
ATAACTGTGGTAATTCTAGAGCTAATACATGCGATCAAGCTCCGAACTCACGTGACGAGTGCATTTATTAGAACAAGACCATCCGGCTT
CGGCCGTTCTTTGGTGACTCTGAATAACTACGCGAATCACATGGTCCTCGCACCGGTGATGTATCTTTCAAGTGTCTGCCTTATCAACT
TCCGATGGTAGTTTATGTGCCTACCATGGTTGTAACGGGTAACGGGGAATTAGGGTTCGATTCCGGAGAGGGAGCATGAGAAACGGCTA
CCACA
